# Flight of the dragons: a global review of migration in Odonata

**DOI:** 10.1002/brv.70170

**Published:** 2026-05-29

**Authors:** Johanna S.U. Hedlund, Myles Menz, Jason W. Chapman, Alexander Hayward

**Affiliations:** ^1^ Department of Evolutionary Ecology and Infection Biology Lund University Ecology Building, Sölvegatan 37 Lund SE‐223 62 Sweden; ^2^ Centre for Ecology and Conservation, University of Exeter Penryn Campus Penryn TR10 9FE UK; ^3^ College of Science and Engineering, James Cook University Townsville Queensland 4811 Australia; ^4^ Department of Migration Max Planck Institute of Animal Behavior Am Obstberg 1 Radolfzell am Bodensee 78315 Germany; ^5^ Department of Entomology Nanjing Agricultural University Nanjing China

**Keywords:** movement ecology, animal migration, Odonata, dragonflies, damselflies, dragonfly migration, insect migration, entomology

## Abstract

Insects are the most abundant and ecologically important animal migrants. Yet, we know relatively little about the patterns and processes underlying insect migration. Dragonflies (Anisoptera) and damselflies (Zygoptera) comprise the ancient insect order Odonata, whose ancestors were the first organisms to fly on Earth. Several members of Odonata are known to migrate long distances and have considerable impacts on ecosystems through biomass and nutrient transfer, pest control and species interactions. However, most aspects of odonate migration remain unknown and available data have not been fully reviewed from a global perspective in over two decades. This lack of consensus has repercussions on species monitoring, specialised conservation efforts and scientific progress. Here, we review odonate migration ecology, addressing: (*i*) what odonate migration is; (*ii*) why odonates migrate; and (*iii*) which odonate species migrate. We define two types of odonate migration: multi‐generational migration, where back‐and‐forth migratory journeys are completed over several generations, and single‐generational migration, where the same individuals leave and return to the original reproductive habitat. We conclude that within single‐generational migration, altitudinal migration is currently the only known strategy, and we present the first complete list of species observed to perform this type of migration, where refuge is temporarily sought at high‐altitude sites away from the reproductive habitat. In addition, we generate an exhaustive global list of 85 dragonfly and 15 damselfly species for which migration has been confirmed (total = 100 species) and a list of 85 possible migrants (22 damselfly species; 63 dragonfly species). Consideration of phylogeny suggests that migration has evolved multiple times within Odonata, and is present in four extant dragonfly families and two extant damselfly families. Approximately 1.5% of all odonate species are migratory, with the proportion rising to approximately 2.9% if species deemed to be possible migrants are also included. Thus, overall, migration is a relatively uncommon strategy in Odonata. Among dragonflies, the vast majority (73%) of migratory species occur in the Libellulidae with 62 confirmed migratory species, equating to 5.9% of all libellulids, whereas in damselflies, migrants are divided almost equally between Coenagrionidae (*N* = 8) and Lestidae (*N* = 7), with the genus *Ischnura* in Coenagrionidae having the most migrants of all damselfly genera (*N* = 5). Biogeographically, the proportion of migratory species is highest in the Palearctic (10.8%), followed by the Nearctic (6.8%), results that may reflect research bias or indicate that migration is an adaptation favoured at high latitudes. Interestingly, most odonate migrants appear to be species of ‘Least Concern’ according to the IUCN *Red List* and are potentially resilient to environmental change because of adaptations associated with their migratory strategy, such as opportunism, generalism and long‐distance mobility.

## I. INTRODUCTION

Research into insect migration has gained momentum over recent years, propelled by advances in tracking technology, radar science and large‐scale monitoring (Haest *et al*., 2024; Hu *et al*., 2016; Huestis *et al*., 2019; Wotton *et al*., 2019). It has also been fuelled by a growing interest in insects and increased understanding of their diverse and important ecological roles and how devastating a collapse of insect populations would be (Goulson, 2019). Studies focusing on the movement of various insect groups (e.g. butterflies, moths, hoverflies, mosquitoes, locusts) have revealed the magnitude of insect migration, both in terms of the sheer quantities of insect biomass that are translocated, and its ecological significance (Hollland, Wikelski & Wilcove, 2006; Hu *et al*., 2016; Wotton *et al*., 2019). While the implications of insect movement ecology are becoming apparent across applied and theoretical research areas, from conservation to pest management and evolutionary theory, increased attention has also exposed the many gaps in our understanding of the movement of insect migrants. Thus, at present, knowledge of insect migration is highly patchy, and only two groups (Diptera and butterflies) have so far been comprehensively evaluated for the prevalence of migration (Chowdhury *et al*., 2021; Hawkes, Menz & Wotton, 2025).

Dragonflies and damselflies (order Odonata, herein referred to collectively as odonates) are widespread and locally abundant insects worldwide. Odonates have important ecological functions, including predation of vectors of human diseases (e.g. mosquitoes), on which they can exert substantial biocontrol (May, 2019; Priyadarshana & Slade, 2023; Simaika & Samways, 2008), and as prey for other organisms (Clarke, Prince & Clarke, 1996; Corso, 2011; Samraoui *et al*., 2022). The enormous size of some migratory swarms of odonates, reaching millions or even billions of individuals (Dumont & Hinnekint, 1973; Holland *et al*., 2006), is even thought to shape the migration of their predators (Clarke *et al*., 1996; Jaramillo, 1993; Smith, 1985). As odonates are sensitive to water pollution, they are widely used as indicator species for water quality, with the conspicuousness of the adults a particular advantage that facilitates monitoring (May, 2019). The large size, aerial abilities and colourfulness of adult odonates has also granted dragonflies and damselflies a level of cultural significance, inspiring human engineering, poetry and art (May, 2019). Their popularity has enhanced biodiversity monitoring and knowledge (several countries have a dragonfly interest group), and they can act as flagship species for the conservation of insects (Clausnitzer *et al*., 2009) which like most non‐vertebrate taxa are often neglected when establishing protected areas (Chowdhury *et al*., 2023).

Despite their apparent popularity, it is somewhat surprising that so little is known about odonate migratory behaviour. The most recent assessments of odonate migration (Corbet, 2004; May & Matthews, 2022; May, 2013; Russell *et al*., 1998; Schröter, Bokenstein & Jödicke, 2023) refer to causal factors behind migratory behaviours as being ‘mysteries’ or ‘unknown’, signalling a need for further research. Most existing knowledge on odonate migration comes from studies of just two species: *Anax junius* (the green darner) and *Pantala flavescens* (the globe skimmer). However, a variety of migratory syndromes are present in other odonate species, although often with only scattered records. There is a need for an updated synthesis, given that three of the most recent assessments had a North American emphasis (May, 2013; Russell *et al*., 1998) and/or a a focus on the suborder Anisoptera (May & Matthews, 2022).

Herein, we provide a systematic review summarising knowledge of the global diversity of migratory odonates, across the 6,390 recognised species in this order (Bybee *et al*., 2021; Paulson, *et al*., 2026). This includes investigation of the biogeographic and phylogenetic distribution of migratory odonates, and their type of migration. The review is structured around the following three major questions: what is odonate migration, why do odonates migrate, and which species migrate?

## II. WHAT IS ODONATE MIGRATION?

The term ‘migration’ has been applied to different behaviours and behavioural outcomes in biology, and there is ongoing debate on how to define this phenomenon and distinguish it from dispersal. The extensive life‐history diversity among species that perform migratory movements presents challenges in providing a widely applicable definition of migration, but this is necessary to facilitate inter‐taxon comparisons and consensus. In this review, we use the broad definition of migration proposed by Kennedy (1985), and refined and expanded by Dingle (2014). This definition focuses on the behaviour of the individual, as it is on this level that natural selection acts, rather than a population outcome such as dispersal or range expansion. This definition was adopted in other reviews of insect migration (Chapman, Reynolds & Wilson, 2015; Chowdhury *et al*., 2021; Hawkes *et al*., 2025; Menz *et al*., 2019). Dingle (2014, p.14) defined migratory behaviour as: *persistent and straightened‐out movement affected by the animal's own locomotory exertions or by its active embarkation on a vehicle. It depends on some temporary inhibition of station‐keeping responses, but promotes their eventual disinhibition and recurrence*.

This definition allows the focal individual to enter and leave a state of migration. When migrating, the individual is largely unresponsive to cues that would otherwise cause it to settle or otherwise disrupt a journey, but as noted by Gatehouse (1997) the word ‘some’ is key in the quote. For example, in insects, nocturnal migrants may halt migration during the day to take shelter, with the reverse being true for day‐flying migrants, and insects migrating close to the ground may feed *en route* (Corbet, 1984a; Gatehouse, 1997). Noting this complexity, Gatehouse (1997, p. 485) stated that *in spite of these interruptions, the expression of migratory behaviour is not terminated until station‐keeping responses are permanently disinhibited and recur*. The definition of migration used herein places no emphasis on the distance covered by the migrant, and thus incorporates journeys ranging from just a few kilometres to those traversing entire continents. Describing how the migrant achieves translocation as involving its *own locomotory exertions or by its active embarkation on a vehicle* allows the definition to include migration accomplished solely by self‐powered movement (e.g. flapping flight, walking, swimming) but also when assisted by winds or currents, the use of which may involve limited or no self‐powered locomotion or control.

Corbet (2004) provided a thorough review of migration in Odonata, defining migratory behaviour as a non‐trivial inter‐habitat spatial displacement, i.e. leaving the habitat where emergence took place and moving to a new habitat for reproduction. He distinguished migration from trivial spatial displacement (e.g. for predator escape/avoidance, oviposition or foraging) and non‐trivial intra‐habitat spatial displacement, i.e. maiden flights and commuting within the home range. In this review, we follow Corbet (2004) on these categories, with the exception of one type of non‐trivial spatial displacement, categorised by him as not being *true* migration but an intra‐habitat ‘seasonal refuge flight’. This type of spatial movement, where individuals leave their emergence habitat in an immature state to fly to a new habitat where they mature, and then return to the emergence habitat, is a behavioural trait also found in other insects, and is generally an adaptation to escape seasonally adverse conditions at breeding sites. The monarch butterfly (*Danaus plexippus*) and the Bogong moth (*Agrotis infusa*) perform seasonal movements of this type and are widely considered to be migratory (Majewska & Altizer, 2019; Warrant *et al*., 2016). Therefore, we include odonate ‘seasonal refuge flights’ as migratory behaviour when the behaviour corresponds to the above definition (Dingle, 2014).

### (1) Types of migration

The great majority of the ~6390 described species of Odonata (Paulson *et al*., 2026) historically have not been considered migratory (Corbet, 2004; May, 2013). For odonate species where records and suggested instances of migration are available there is considerable variation in the expression of migratory behaviour. As for other migratory insect taxa (Holland *et al*., 2006), the most common form of migration in dragonflies and damselflies takes place across multiple generations, where subsequent generations return to an area or region from which the parent generation originated. However, as will be discussed further below, return migration within the same generation is also present in some odonates. Further, migration in odonates can be facultative or obligate, take place in the immature or mature state, occur as either a collective behaviour (with great numbers of individuals travelling together *en masse*) or in a solitary form, with migrating individuals separated spatiotemporally. In the following subsections this variation will be described.

#### (a) Multi‐generational migration

In insects, migrant individuals usually do not make round‐trip journeys and so do not return to the area that they departed from (Holland *et al*., 2006), instead, the return journey is performed by subsequent generations. This is in contrast to the typical form of migration in vertebrates, where the same individual leaves and returns to the same breeding area, often repeatedly across multiple years. As insect lifespan is short, in many cases their migratory circuits can only be completed over several generations. Multi‐generational migration is likely the most common migration system in Odonata (Corbet, 2004), but there is a lack of information for most species. *A. junius* is one of the few well‐studied odonate taxa in terms of migratory behaviour, and this species performs a complex and multi‐generational migratory circuit of the Americas (Hallworth *et al*., 2018; Hobson *et al*., 2012b). Its migration is described in detail in Section III, but involves migration northwards in spring, migration southwards in summer/autumn and also residence, demonstrating impressive flexibility in migratory syndrome, with individuals able to regulate migratory behaviour depending on latitude or to refrain entirely from migrating (Hallworth *et al*., 2018; Holland *et al*., 2006). It may be assumed that other odonate migrants that experience similar ecological challenges and similar climates also will have evolved flexible, facultative and multi‐generational migration systems. Likely candidate species include *Pantala hymenaea*, *Tramea lacerata*, *Sympetrum corruptum* (May, 2013), *Aeshna mixta* (Knoblauch, Thoma & Menz, 2021; Oelmann *et al*., 2023) and *Sympetrum striolatum* (Knoblauch *et al*., 2021; Schröter *et al*., 2023).

**Fig. 1:**
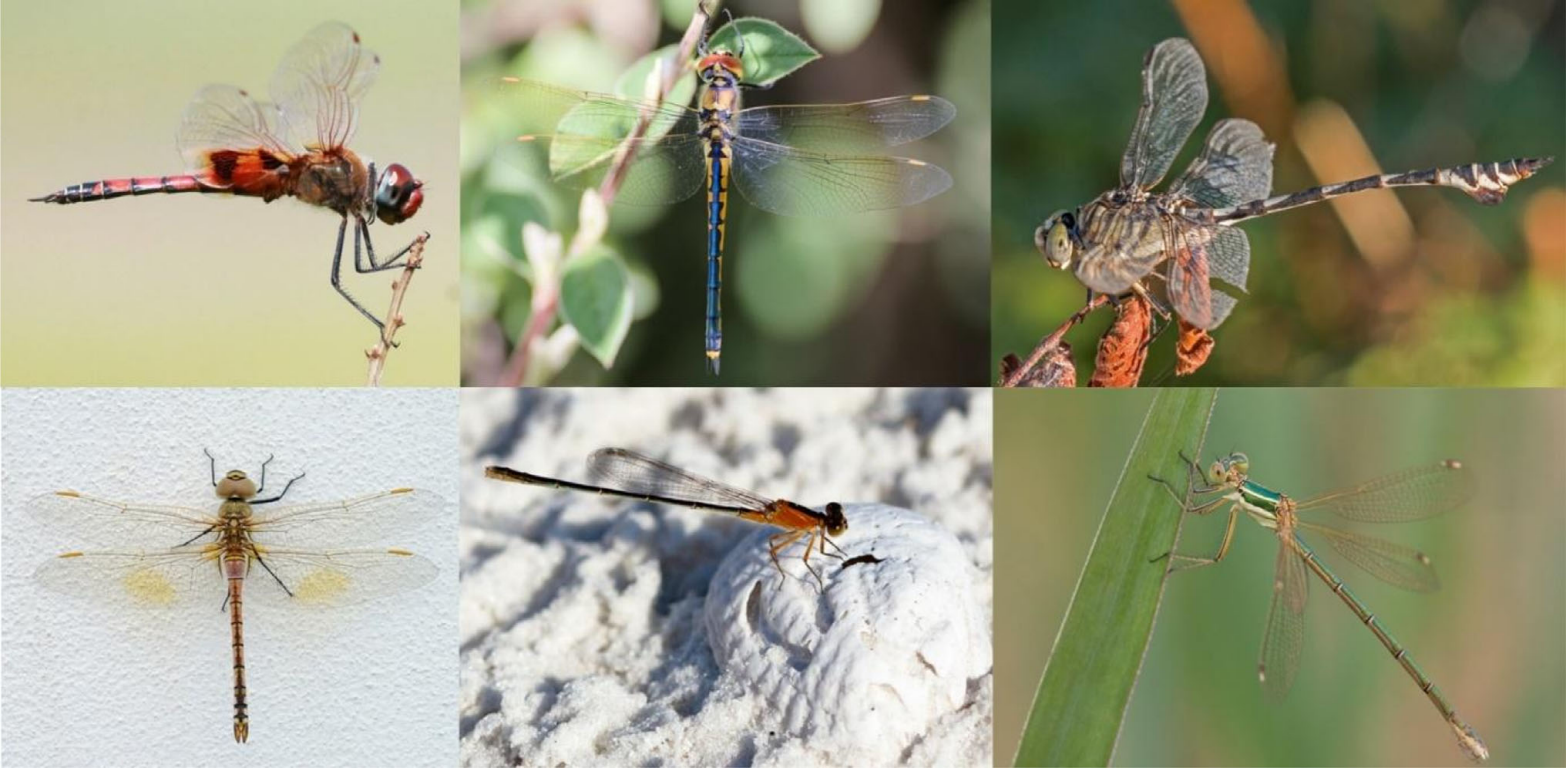
Migratory species from the six odonate families with confirmed migrants: top left Libellulidae: *Tramea basilaris* (photograph by Charles J. Sharp under licence CC BY‐SA 4.0); top middle Cordulidae: *Hemicordulia tau* (photograph by Thomas Jaeger under licence CC BY‐SA 4.0); top right Gomphidae: *Lindenia tetraphylla* (photograph by Charles J. Sharp under licence CC BY‐SA 4.0); bottom left Aeshnidae: *Anax ephippiger* (photograph by Alvesgaspar under licence CC BY‐SA 4.0); bottom middle Coenagrionidae: *Ischnura ramburii* (photograph by Melissa McMasters under licence CC BY 2.0); bottom right Lestidae: *Lestes barbarus* (photograph by Christian Fischer under licence CC BY‐SA 3.0). All photographs have been cropped.

In *A. junius*, multi‐generational migration appears to be an adaptation enabling reproduction in areas that are too cold for year‐round residence (Corbet, 2004; Hallworth *et al*., 2018; see Section III), but migration over several generations also is adopted in areas prone to droughts. In tropical and semi‐tropical areas, several odonate species are known to show seasonal mass‐emergence and utilise wind systems to translocate themselves away from dry areas towards where the next rain will fall. This strategy has been proposed for *P. flavescens*, *Anax ephippiger*, *Diplacodes luminans* and *Tramea basilaris* (Fig. 1) (Corbet, 1984a, 2004; Gambles, 1960; Suhling & Martens, 2007). *P. flavescens*, a globally distributed and locally abundant species, has been proposed to perform the longest body‐length‐specific non‐stop migratory flight in the world, crossing the Indian Ocean between India and East Africa (Anderson, 2009; Hedlund *et al*., 2021) and most likely also other oceans (Ware *et al*., 2022). Truly astounding long‐distance migratory journey are performed by species engaging in multi‐generational migratory circuits: *P. flavescens* is hypothesised to travel ~6000 km within one generation and *A. junius* ~680 km in one generation (Hallworth *et al*., 2018; Hedlund *et al*., 2021; Hobson *et al*., 2012, 2012).

There is great diversity in multi‐generational migration in odonates. Within a single species, both migrants and sedentary individuals may exist, and cohorts/ generations where a majority or minority migrates (Hallworth *et al*., 2018), i.e. migration is facultative, as is common among migratory insects (Menz *et al*., 2019) However, for certain species, for example semi‐tropical odonate species that follow seasonal rains, migration has been suggested to be obligate (Corbet, 2004), i.e. rigidly expressed at a particular life‐history stage. Importantly, it is currently widely accepted that variation in animal migrants has a genetic basis (Doyle *et al*., 2022), thus both facultative and obligate migratory strategies are expected to be regulated genetically (Menz *et al*., 2019). For odonates, molecular studies on migration have mostly focused on outcomes, such as patterns in gene flow (e.g. Ware *et al*., 2022) rather than gene regulation and mechanisms that shape migration itself (Merlin *et al*., 2019). Hence, knowledge of the genetics underlying odonate migration remains limited. *P. flavescens* provides an opportunity to bridge this information gap, as it is both a well‐known odonate migrant and a focus of molecular study (Cao, Fu & Wu, 2015; Troast *et al*., 2016; Ware *et al*., 2022). Interestingly, even in this presumed obligate migrant, there are reports of one population becoming sedentary. On Easter Island, situated in extreme isolation and with perpetually suitable breeding habitats, *P. flavescens* appears to have lost migratory behaviour completely (Dumont & Verschuren, 1991), demonstrating that migratory behaviour may be lost even in obligate migrants.

#### (b) Single‐generational migration and altitudinal migration

Despite multi‐generational migration being the most common form of migration in insects (Holland *et al*., 2006), it is not the only type of migratory behaviour expressed within the class Insecta; some species complete a migratory circuit within a single generation. Single‐generational migration occurs when an individual migrates to take refuge away from its breeding habitat temporarily during adverse conditions, often delaying maturation. Adverse conditions may include high temperatures, other factors threatening adult survival, or temporary degradation of breeding sites in times of drought. When the breeding habitat again becomes suitable, the individual will return, becoming sexually mature either before or after returning. When these types of movements specifically occur on mountains, they are described as ‘vertical migration’ or ‘altitudinal migration’, and have been described in several insect orders (Brown, 1962; Ceryngier, 2015; Chowdhury *et al*., 2021; Dreyer *et al*., 2018), including Odonata (Borisov, 2009; Borisov, Kazenas & Borisov, 2022; Corbet, 2004). Altitudinal migration is a widely accepted concept within movement ecology, referred to as a form of short‐distance migration and is best known in birds (Hsiung *et al*., 2018).

Notably, as well as including a completed round‐trip journey within one generation, for odonates single‐generational migration differs from multi‐generational migration in its purpose. Whereas multi‐generational migration is a spatial displacement between habitats to reach a new area for reproduction, single‐generational migration is a spatial displacement between habitats to reach a new area for survival.

For odonates, we identified only one type of single‐generational migration: altitudinal migration. Thus, until additional studies reveal otherwise, single‐generational migration and altitudinal migration can be regarded as synonyms in odonates, and are referred to herein simply as altitudinal migration. However, we acknowledge that some odonate species delay maturation away from their reproductive sites for long periods without engaging in altitudinal migration (see Tables A8.2–A8.3 in Corbet, 2004). In these taxa, immature or maturing adults can be found in non‐breeding habitats close to the reproductive sites, for example in forests or bushland (Clausnitzer, 1999; Corbet, 2004). Future studies may reveal that some of these species, which leave their reproductive habitats during the dry season, may engage in migration according to the definition of Dingle (2014). However, at present there is little knowledge on the behaviour of many of these species during the dry period (Corbet, 2004; Paulson, de Haseth & Debrot, 2014).

Altitudinal migration has been recorded in both dragonflies [e.g. *A. mixta*, *Anax* spp., *Sympetrum* spp. (Samraoui *et al*., 1998; Samraoui & Corbet, 2000)] and damselflies [e.g. *Sympecma* spp., *Lestes* spp. (Kosterin & Borisov, 2010; Samraoui, 2009)]. One example that has become embedded in human culture in the region where it occurs is the seasonal mass migration of *Sympetrum frequens*. A red dragonfly known as *Aka Tombo* appears widely in Japanese art and poetry and is generally considered to be a *Sympetrum* species, likely *S. frequens*. In spring, newly emerged adults (tenerals) of *S. frequens* migrate to the cool highlands in large numbers; when the summer heat abates in the lowland rice fields, they return to reproduce, triggered by the passage of *Akisame* (the autumn rain) (Miyakawa, 1994; Uéda, 1988).

In general, altitudinal migration occurs over shorter distances than multi‐generational migration, e.g. involving travel to and from a lowland breeding habitat *via* a mountain refuge habitat as seen in *S. frequens* (Uéda, 1988), *A. mixta* and *S. striolatum* (Samraoui *et al*., 1998). In *S. frequens* the distance covered can be up to 60 km (Pritchard, 1992). Both altitudinal and multi‐generational migration can be expressed within a single species, depending on environmental conditions. Typically, altitudinal migration is restricted to climates where heat and drought seasonally threaten odonate survival and reproduction, with cooler and wetter higher altitude habitats offering respite from difficult conditions. For example, *A. mixta*, *Anax parthenope* and *S. striolatum* engage in altitudinal migrations in the Mediterranean (Samraoui *et al*., 1998; Samraoui & Corbet, 2000), but in northern Europe the same species migrate southward in autumn as sexually mature individuals (Oelmann *et al*., 2023; Schröter *et al*., 2023; Shapoval & Buczyński, 2012), presumably performing multi‐generational migrations.

Altitudinal migration is poorly researched in odonates, and it remains unclear whether the behaviour is facultative or obligate; even in *S. frequens*, some individuals do not migrate to mountain refuges (Corbet, 2004). In Section IV and the online Supporting Information (see Table S2, Dataset S1), we provide a global compilation of species for which altitudinal migration is known. Note that the total we identified (18 species) is likely an underestimate that will be extended by future research.

### (2) Mechanisms and adaptations for migration

#### (a) Energetics

Migration in odonates is known to involve behavioural, morphological and physiological adaptations (Corbet, 2004). Similar to birds and other insect migrants, odonates can store fat to fuel their journey (Blem, 1980; Corbet, 1984a; Weber, 2009). Fat storage as migratory fuel has been reported in seven species: *A. junius* (May & Matthews, 2022), *P. flavescens* (Corbet, 1984a; Hobson *et al*., 2021), *A. ephippiger*, *T. basilaris* (Fig. 1), *T. lacerata*, *Sympetrum vicinum* and *D. luminans* (Corbet, 1984a). In *A. junius*, 20–30% of total body mass consisted of fat reserves (May & Matthews, 2022). As most odonate migration occurs across land, migrants can adopt an ‘income migration’ strategy (Evans & Bearhop, 2022), stopping at intervals to refuel and rest (Corbet, 1984a; Wikelski *et al*., 2006). It is unknown whether species that cross open water typically store larger fat deposits (Hedlund *et al*., 2021), thus transitioning from ‘income migration’ to ‘capital migration’ (Evans & Bearhop, 2022). Temporary mass gain for the purpose of fuelling endurance flights is well known in birds (Newton, 2008), but remains to be demonstrated in dragonflies. However, many transoceanic dragonfly migrants such as *A. ephippiger* (Hedlund *et al*., 2020; Lambret & Boudot, 2013) and *P. flavescens* (Anderson, 2009) appear successful in surviving long open ocean flights, suggesting that storage of fat is likely an important adaptation in certain migrant odonate species.

#### (b) Navigation

Navigation is a core ability for migratory animals, and in insects a variety of cues are used to guide long‐distance movement (Freas & Spetch, 2023). Odonates show evidence of navigation, engaging in directed flight, i.e. flying southwards in autumn and northwards in spring [e.g. *A. junius* and *A. mixta* (Freeland *et al*., 2003; Knoblauch *et al*., 2021)], and compensation for cross‐wind drift to maintain a preferred flight direction during migration (e.g. *P. flavescens* and *P. hymenaea*) (Srygley, 2003). Many species are able to return to the same water body several months after leaving (Pritchard, 1992; Utzeri *et al*., 1984), suggesting that a form of visual navigational memory is present (Corbet, 2004). Over shorter distances, odonates may follow landscape features such as roads and coastlines during migration (Dumont & Hinnekint, 1973; Wikelski *et al*., 2006). However, the mechanisms by which odonates navigate over long distances remain unclear. It has been hypothesised that they use a time‐compensated sun compass (Corbet, 1984a; Gkanias & Webb, 2025; Hisada, 1972), as is the case in other diurnal insects (Freas & Spetch, 2023), and specialized areas of the odonate compound eye may allow detection of polarized light, indicating that they could exploit this for orientation (Corbet, 2004; Meyer & Labhart, 1993). While there are no studies providing evidence for polarization‐based celestial orientation in odonates (Cezário *et al*., 2025), they are known to locate aquatic habitats by polarotaxis, i.e. orientation using the plane of polarization of polarized light (Bernáth *et al*., 2002).

#### (c) Winds and temperature

Wind speed, seasonality, direction and altitude can all shape migratory outcome and the distribution of migrants in the air, for both weak‐flying and strong‐flying species (Hedlund *et al*., 2021; Reynolds, Chapman & Drake, 2017). Weak‐flying species reliant on winds for their migration usually ascend to higher altitudes where wind speeds are greater (Reynolds *et al*., 2017). Strong‐flying species may be found at high altitudes when actively migrating, but can also descend and utilise their own locomotion. Both dragonflies and damselflies (e.g. *Libellula vibrans*, *Pachydiplax longipennis*, *Perithemis tenera*, and *Ischnura hastata*) have been captured by high‐altitude air sampling by aircraft at 60–300 m above sea level in North America (Glick, 1939), while in East Africa, sticky panels suspended at 90–160 m above sea level also caught odonates (Atieli *et al*., 2023). Vertical‐oriented radar has recorded migratory movements of *P. flavescens* at 200–1000 m above sea level (Feng *et al*., 2006). Odonate migration becomes conspicuous to human observers when it occurs closer to the ground within the flight boundary layer (FBL). The FBL is defined as the low‐altitude zone (approximately 1–10 m above ground) where wind speeds are weaker and where insects can control their flight direction using self‐powered flight. Strong‐flying species, capable of robust self‐powered flight, are less reliant on wind assistance and can even fly against the wind in the FBL (Srygley, 2003). Migrants flying within the FBL are able to retain control over their direction of flight, and besides dragonflies, include for example large lepidopterans (Gatehouse, 1994).

Certain odonate species of tropical and sub‐tropical distribution (e.g. *P. flavescens*, *A. ephippiger*, *T. basilaris* and *D. luminans*, Fig. 1) appear to rely on the prevailing direction of seasonal wind systems for their migration (Corbet, 2004), with the tradewinds being described as a ‘global conveyor belt’ for *P. flavescens* migration and population genetics (Ware *et al*., 2022). The South American dragonfly *Rhionaeschna bonariensis*, reported as forming the largest migrating odonate swarms reaching 4–6 billion individuals (Jaramillo, 1993; Russell *et al*., 1998), has been reported to migrate ahead of the Pampero, a strong north‐easterly wind that appears seasonally and has been linked to migration assistance in birds (Bravo, Cueto & Gorosito, 2017).

Adaptations to wind usage in migrating odonates have been reported, with observations confirming that: (*i*) *A. mixta* choose to migrate when favourable tailwinds are available (Knoblauch *et al*., 2021); (*ii*) *Pantala* spp. can compensate for crosswind drift to remain flying in a preferred direction (Srygley, 2003) and (*iii*) a combination of wind and temperature cues stimulates migration in *A. junius*, a species that appears to fly with the wind rather than correcting for its direction (Wikelski *et al*., 2006). Utilising winds for energy‐efficient gliding flight, and decreasing the metabolic cost of flapping to stay airborne (Gibo, 1981), appears to be reflected in the wing morphology of dragonflies, with migratory species having larger, longer and smoother wings with a larger anal lobe (Huang *et al*., 2020; Suárez‐Tovar & Sarmiento, 2016). In order to cross the Indian Ocean, which is the hypothesised migratory route of *P. flavescens*, migrants have to match their ocean crossing to a spatiotemporal window of favourable winds, suggesting the ability to assess and choose winds appropriately and to use gliding as their main flight mode (Hedlund *et al*., 2021). Metabolic costs associated with a high‐endurance migration could potentially be mitigated if the migrant can feed during its flight. This has been suggested to be possible during their Indian Ocean crossing by *P. flavescens*, due to the presence of other insects in the oceanic air column (Anderson, 2009). Trophic interactions of this kind, also involving predation on dragonflies by migratory insectivorous birds, suggest a tantalizing presence of ‘ecosystems in the sky’, i.e. seasonal, air‐borne mobile ecosystems.

Temperature can affect odonate migration *via* the minimum ambient temperature at which flight can be sustained (Reynolds *et al*., 2017). Their relatively large body size compared to many insects reduces their surface area to volume ratio, lowering heat loss and thus enabling them to fly at lower ambient temperatures (May, 1976). Large species are able to fly at temperatures as low as ~10 °C (e.g. *A. ephippiger*; Dumont, 1988).

The passage of cold fronts and warm air masses have been linked to the migration of odonates in both temperate and tropical climates (Corbet, 2004; May & Matthews, 2022; Moskowitz *et al*., 2001; Reels, 2010; Russell *et al*., 1998; Williams, 1957). Changes in atmospheric pressure may act as cues for migratory departure in odonates (Dumont & Hinnekint, 1973), as has been demonstrated for other insect migrants, for example the potato leafhopper (*Empoasca fabae*). In this small migrant, entirely dependent on winds for migration, autumn migration is initiated in response to a reduction in air pressure that accompanies the passage of weather fronts and persistent northerly winds, which carry the leafhoppers to their southern destination (Shields & Testa, 1999). Similarly, *A. junius* migration appears to be triggered following two nights of decreasing temperatures, likely an indication of continuous northerly airflows (Wikelski *et al*., 2006).

## III. WHY DO ODONATES MIGRATE?

Migration in vertebrates it is often correlated with high mortality (Klaassen *et al*., 2014). In odonates, ocean‐crossing flights of *P. flavescens* require precise spatiotemporal wind conditions and may result in a high frequency of ocean landings and death (Hedlund *et al*., 2021), and have even been suggested to be maladaptive (Fraser, 1954). Why then do odonates migrate? In fact, many migratory insects produce several generations per year as they are able to move to exploit resources where these are most abundant (Chapman *et al*., 2015; Suhling *et al*., 2015). Thus, migratory insects like *P. flavescens* (Ware *et al*., 2022) often have large geographical ranges and substantial populations.

Migratory tendency often shows variation within populations, with partial migration (part of the population migrates, while some individuals remain resident) being widespread in migratory insects and in odonates (Menz *et al*., 2019; Oelmann *et al*., 2023; Walter, 1996). Migratory and resident populations in *A. junius* can arise due to differences in developmental strategies, with migrants developing rapidly within a season and residents developing more slowly and entering diapause to emerge the following year (Hallworth *et al*., 2018; Trottier, 1971). Interestingly, analysis of mitochondrial data revealed no distinct differences between resident and migratory phenotypes (Freeland *et al*., 2003), suggesting a strong environmental influence on these strategies. The underlying causes of partial migration can be complex, and driven by environmental and genetic factors (Chapman *et al*., 2011). Migrants and residents of the same insect species may differ in behaviour, morphology and physiology (Menz *et al*., 2019). In dragonflies, a population of a migratory species (*P. flavescens*) became sedentary on an isolated island, with accompanying changes in wing and body morphology (Alvial *et al*., 2017). Apart from two separate observations in *A. mixta* (Dyatlova & Kalkman, 2008; Oelmann *et al*., 2023), there are no reports of odonate migration being sexually biased, as is known for some insect migrants such as hoverflies (Doyle *et al*., 2025; Hlaváček, Lučan & Hadrava, 2022).

Departure decisions in migratory odonates have not been experimentally studied, but it has been suggested that a combination of environmental cues (i.e. temperature, atmospheric pressure) and photoperiod may act as triggers. Studies on tagged individuals indicated that persistent reductions in temperature elicit migration in *A. junius* (Wikelski *et al*., 2006). Photoperiod may also influence migration indirectly through its effects on the phenology of emergence and larval development (May & Matthews, 2022). Some species exhibit synchronised emergence [e.g. *Aeshna affinis*, *Sympetrum danae* (Bernard & Samolag, 1997; Michiels & Dhondt, 1989; Schröter, 2011)], and this phenomenon has been linked to swarm migrations in odonates (Corbet, 2004; Schröter, 2011).

In Odonata, migration likely evolved in response to seasonal unsuitability of reproductive habitats (Corbet, 2004). In temperate regions, reproductive habitats commonly become unsuitable during periods of cold temperatures, whereas unsuitability for reproduction in tropical and sub‐tropical regions most often relates to extended drought periods in combination with high temperatures. In temperate regions, migration therefore commonly occurs across latitudes, whereas migration in tropical and sub‐tropical regions is governed by the spatiotemporal distribution of rain. Importantly, it is the interacting effects of seasonal changes in rain and temperature that determine the suitability of reproductive habitats for odonates, and throughout the range occupied by a single species, migration may occur across both latitude and spatiotemporally in response to rainfall, or, as in the case of altitudinal migrants, in response to both temperature and rainfall. In addition to circumventing discontinuous habitat suitability, biotic factors such as overcrowding, parasite load and social cues have also been proposed as factors eliciting migratory behaviour in odonates.

### (1) Seasonal temperature

During winter in temperate regions, air temperatures fall far below the minimum that can support adult activities in dragonflies (Norling, 1984). During these harsh months, species can enter a stage of down‐regulated activity called diapause (often referred to as ‘hibernating’ in odonates), as eggs, larvae, or sometimes, as adults (Corbet, 2004). Alternatively, species may migrate to and from northern breeding grounds, utilising them only when they are suitable.

*A. junius* breeds in shallow lakes and ponds and is widespread from Central America to southern Canada. This species provides a good example of migration in response to seasonal temperature changes. At the northern limit of the species' range (39–47 °N), males and females arrive from the south on strong warm winds in spring and breed well before other local odonates have emerged (Corbet, 2004). The progeny of these migrants emerge at the height of summer, after which they may stay and breed, or accumulate fat reserves to fly south to breed. Further south, adults emerge from overwintering larvae from February (~27° N) to July (~40° N), and may breed locally or migrate north, but at the end of the year, adults emerging in the south will refrain from migrating entirely and only breed locally. Thus, in certain areas, breeding populations may consist of both locally emerged adults and migrants, and there will be temporal and spatial fluxes of this mixture throughout the species' range, recently investigated using stable isotope analysis (Hallworth *et al*., 2018). There appear to be no genetic difference between migratory and sedentary cohorts (Freeland *et al*., 2003; May & Matthews, 2022). Distances flown by a single *A. junius* individual may exceed 380 km (Hallworth *et al*., 2018) and during migration, individuals may aggregate in huge numbers (Russell *et al*., 1998). *A. junius* appears to be sexually active throughout its migration, suggesting that they may breed along the migratory route and not only at a set destination (Holland *et al*., 2006; May & Matthews, 2022).

Several temperate dragonfly species are known to migrate across latitudes, i.e. move southwards in autumn, e.g. *A. mixta*, *Sympetrum vulgatum*, *Libellula quadrimaculata* (Knoblauch *et al*., 2021; Shapoval & Shapoval, 2017) and *Libellula pulchella* (Russell *et al*., 1998), and northwards in spring, e.g. *P. longipennis* (Taber, 2002) and *Epiaeschna heros* (Russell *et al*., 1998). However, their phenological population structure, regularity of their migration, and the distances covered, are not well known.

### (2) Seasonal rain

The Intertropical Convergence Zone (ITCZ) is a front system that moves seasonally back and forth across the equator, directing trade winds and seasonal rains southwards or northwards. Certain dragonfly species have adapted their life cycle to take advantage of the ITCZ, riding the trade winds to where the next rain will fall. *A. ephippiger*, *P. flavescens*, *T. basilaris*, *Tholymis tillarga* and *D. luminans* all show pronounced ITCZ migrations, but several other species breeding in tropical and semi‐tropical areas may adopt a similar strategy (Corbet, 2004). A close look at the lifecycle of *A. ephippiger* exemplifies the rain‐following migration strategy; *A. ephippiger* is widespread in the Paleotropics, and the larvae develop rapidly in ephemeral, brackish water bodies, usually emerging as adults after about 3 months (Dumont, 1977; Dumont & Desmet, 1990). Migration is initiated in the early post‐teneral state and within the species core range (Africa and Asia) they travel on rain‐bearing weather fronts, sometimes aggregating in huge swarms (Corbet, 2004; Dumont, 1977). Adults feed during migration (Corbet, 1984a) and can become sexually mature and commence breeding, but then continue migration after mating/egg‐laying (Corbet, 2004; Dumont, 2014).

Similarly to *A. junius*, the migratory range of *A. ephippiger* incorporates vast distances and the species may complete a migratory circuit that includes Europe, Asia and Africa (Dumont, 1977; Dumont & Desmet, 1990; Lambret & Deschamps, 2013). The tendency of this species to enter seasonal wind systems, results in impressive (one way) trans‐oceanic journeys, including reports of passage over the Indian Ocean from the Asian mainland to the Maldives (Anderson, 2009) and even from Africa to south and central America across the Atlantic (Hedlund *et al*., 2020). The regularity of these ocean crossings remains unclear, and return flights are yet to be documented, but it is presumably the migratory tendencies of these species that resulted in the far‐reaching translocations.

In the Western Sahel, large aggregations of *A. ephippiger* can be seen in January–March, about 3 months after the end of the rainy season, and migrants are subsequently observed along the West African coast, and then in southern Europe (Corso *et al*., 2012; Dumont, 1977; Lambret & Deschamps, 2013; Mediani *et al*., 2012). Following the arrival of African migrants to the Mediterranean, a multi‐generational migration northwards through Europe ensues, ending with rare vagrants being observed as late as November and as far north as the UK (Parr, 2019). Migration south through Europe is seldom observed, but there are reports from Eastern Europe (Günther, 2005; Kürschner, 1977; Peters & Günther, 2000); it is not known whether the ill‐fated individuals that arrived at unsuitable British breeding grounds in winter originated from within or outside Europe. A long‐distance multi‐generational, migratory round‐trip journey connecting the Sahel with Northern Europe is known in the butterfly *Vanessa cardui* (Gorki *et al*., 2024; Talavera *et al*., 2018), but further evidence is needed to confirm this astounding migratory circuit in *A. ephippiger*.

### (3) Entrainment, density and disease

Insect migration is most easily observed when it occurs *en masse*. Historical records of large swarms of odonates are plentiful (Beutenmuller, 1890; Calvert, 1893; Dumont & Hinnekint, 1973; Hudson, 1895; Russell *et al*., 1998; Shannon, 1916) and migrant dragonflies moving in swarms have been reported from all continents where they exist: North America (Russell *et al*., 1998); South America (De Marmels, José & Sharpe, 2008; Hudson, 1895; Rudolph & Fisher, 1993); Europe (Corbet, 2004; Dumont & Hinnekint, 1973); Africa (Corbet, 1984a; Onyango‐Odiy, 1973; Samways, 1992); Asia (Anderson, 2009; Mitra, 1994); and Oceania (Gibson‐Hill, 1950). A swarm can be single species (de Marmels *et al*., 2008; Hudson, 1895), or multi‐species (Corbet, 1984a; Palacino & Millán, 2010; Paulson, 2002) sometimes with one species making up the majority (Haritonov & Popova, 2011). This has raised suggestions of attraction‐directed behaviours (Corbet, 1962; Suhling, Martens & Suhling, 2017), in which the visual cue provided by a large swarm attracts conspecific and non‐conspecific individuals that would not otherwise have made the decision to leave. The word ‘entrainment’ has been used to describe this phenomenon. Whether entrainment alone would stimulate migration in an otherwise sedentary species is unclear, but the concept has been used to explain the formation of large swarms.

Alongside entrainment, over‐crowding has been discussed as a trigger for swarming and migratory behaviour in insects (Gatehouse & Zhang, 1995), but studies investigating causality in odonates are few. Only two species have been studied in this context: *L. quadrimaculata* and *A. junius*. A 30‐year study performed in Siberia showed that high‐density years were correlated with increased migratory activity, specifically when population density reached a critical aggregation of more than 3 individuals per m^2^ in *L. quadrimaculata* (Haritonov & Popova, 2011). *L. quadrimaculata* is well known for appearing in large swarms (Buczyński, Shapoval & Buczyńska, 2014; Dumont & Hinnekint, 1973; Shapoval & Shapoval, 2017) and a previous analysis of long‐term, pan‐European records suggested a cyclic pattern in swarm movements (Dumont & Hinnekint, 1973), supporting a role of density‐dependent factors. Thus, migration, at least partly, density‐dependent in *L. quadrimaculata* and this factor should be considered in other species, for example in *A. affinis* where mass emergence has been observed to be correlated with mass migration (Schröter, 2011). However, for the only other species where density has been considered as a factor in migration, *A. junius*, high early emergence density did not predict early migration (May & Matthews, 2008), indicating that other factors may be involved.

High density is also related to the prevalence of parasites (Majewska *et al*., 2022). In butterflies, individuals may escape seasonal build‐up of parasites by migrating (Bartel *et al*., 2011; Stefanescu *et al*., 2012). Temporal increase in parasite load has been linked to the cyclic mass‐movements of *L. quadrimaculata* (Dumont & Hinnekint, 1973), although this correlation was not investigated further and it is unlikely that it was the sole reason for migration (Loehle, 1995).

Given the broad definition of migration as an individual behaviour used herein (Dingle, 2014), cues triggering migration may act on one individual or simultaneously on an enormous number of individuals, and actively migrating odonates can be solitary (Devaud & Lebouvier, 2019; Norling, 1967) or fly together in swarms (Russell *et al*., 1998). Extraordinary finds of solitary individuals, for example *A. ephippiger* encountered on Iceland (the only odonate ever recorded there) (Norling, 1967) or *P. flavescens* reported from Amsterdam Island in the Indian Ocean (Devaud & Lebouvier, 2019), likely represent instances of migrants translocated by strong winds. Importantly, for both these species, there is ample evidence of migration (Anderson, 2009; Corbet, 2004; Dumont, 1977; Hedlund *et al*., 2020; Ware *et al*., 2022), and these two examples of remote long‐distance translocations were presumably enabled by the individuals' decision to migrate. The population outcome of remotely translocated individuals may ultimately be dispersal/range expansion, as seen in *A. ephippiger*'s colonisation of the Caribbean (Hedlund *et al*., 2020) or *I. hastata*'s establishment on the Azores (Corbet, 2004; Cordero Rivera *et al*., 2005).

## IV. WHICH SPECIES MIGRATE?

Migratory behaviour in odonates has been recorded most commonly in the sub‐order Anisoptera, the true dragonflies, and especially in the families Libellulidae and Aeshnidae (Corbet, 2004; May, 2013; Russell *et al*., 1998). The smaller size and more delicate anatomy of damselflies (sub‐order Zygoptera) has attracted doubts regarding their ability to apply precise migratory control (Corbet, 2004; Góral, 2025), however, there is clear evidence for migration within this taxon. For example, damselflies have been observed to ascend vertically until lost to the naked eye (Cham, 1991), a behaviour that has been interpreted as migratory take‐off (Corbet, 2004; Fox, 1989; Yazicioğlu, 1982). In addition to these perceived departure behaviours, aerial sampling has recorded damselflies 70–915 m above the open ocean (Glick, 1939) and a number of damselfly species have been described as migratory or observed to migrate (Beukema, 2007; Corbet, 2004; Fraser, 1933; Góral, 2025).

Previous estimates of the total number of migrant odonate species vary from 25 to 50 species (0.03–0.07% of odonates) (Corbet, 2004; Kormondy, 1961) to up to 100 species (1.5%) (Russell *et al*., 1998). We reviewed the available literature to investigate the prevalence and distribution of migratory behaviour in Odonata, and here provide an overview of current knowledge, and draw conclusions on the phylogeny and biogeography of odonate migration.

### (1) Literature review

A list of assumed migrant species was collected from four original literature sources (Corbet, 2004; Dumont & Hinnekint, 1973; May, 2013; Russell *et al*., 1998). Targeted searches were performed on all these species in *Google Scholar* during 2019–2020 and 2022–2023, using the species' scientific name coupled with the term ‘migra’. English was the main language of detected publications, but literature in other languages was also included. The list was extended when statements referring to additional species were encountered during the search. All species were investigated thoroughly; the search was only halted if reliable evidence of migratory activity was found or if all sources had been exhausted. Each investigated species was categorised as a migrant, possible migrant or as a species with no evidence of migration, as determined on the level of empirical evidence (Table 1). Importantly, studies providing proof of migration meeting Dingle's (2014) definition are rare for insects (Gatehouse, 1994). For example, extensive laboratory experiments would be necessary to corroborate that an individual is unresponsive to station‐keeping cues, but the types of evidence listed in Table 1 provide a reliable proxy for migration.

**Table 1:** Categories for evidence of migratory activity used in the literature search. Investigated species were placed into one of three categories (confirmed migrant, possible migrant or no evidence of migration) based on the identified evidence.

Category	Description of evidence
Confirmed migrant	The species was designated as a confirmed migrant if it fulfilled any of the following statements: Recent records of directed movements/migration/*en masse* appearances, arrivals or departures Seasonal and repeated collection in Heligoland/Rybachy traps* Stable isotope or genetic analyses indicate migration Phenology indicates migration (e.g. adults appear in regions where no larvae can survive the winter/dry period) Translocation to altitudinal habitat for maturation (i.e. immature/maturing adults appear seasonally at higher altitudes, away from reproductive habitats)
Possible migrant	The species was designated as a possible migrant if it fulfilled the criteria outlined in (*i*), or several of the clauses under (*ii*): (*i*) Unconfirmed historic record of directed movements/migration/*en masse* appearances, arrivals or departures (*ii*) Species has been caught in light traps/aerial nets; encountered at sea; inhabits remote areas (habitats or islands)
No evidence of migration	If a species has been described as migratory without empirical support, or if circumstantial evidence is provided, but no evidence for categorisation as a ‘Possible migrant’ or ‘Confirmed migrant’ was identified by the directed search

* Large, funnel‐shaped net‐traps facing northwards or southwards, installed to trap and ring migratory birds, but that regularly also catch migratory insects (Oelmann *et al*., 2023; Shapoval & Buczyński, 2012).

The presence of altitudinal migration was also noted, as well as the species' geographical range and IUCN *Red List* status. A species was regarded as an altitudinal migrant (Borisov, 2009; Borisov *et al*., 2022; Corbet, 2004) if there was evidence of individuals translocating from reproductive habitats to mature sexually at higher altitude habitats (see Table 1). Migration was confirmed in a species if it fulfilled the requirements of multi‐generational migration or single‐generational (altitudinal) migration. Where several scientific names are in use or where there was taxonomic ambiguity, we followed the classifications of Bybee *et al*. (2021) and Paulson *et al*. (2026).

### (2) Results of the literature review

#### (a) Overview

**Fig. 2:**
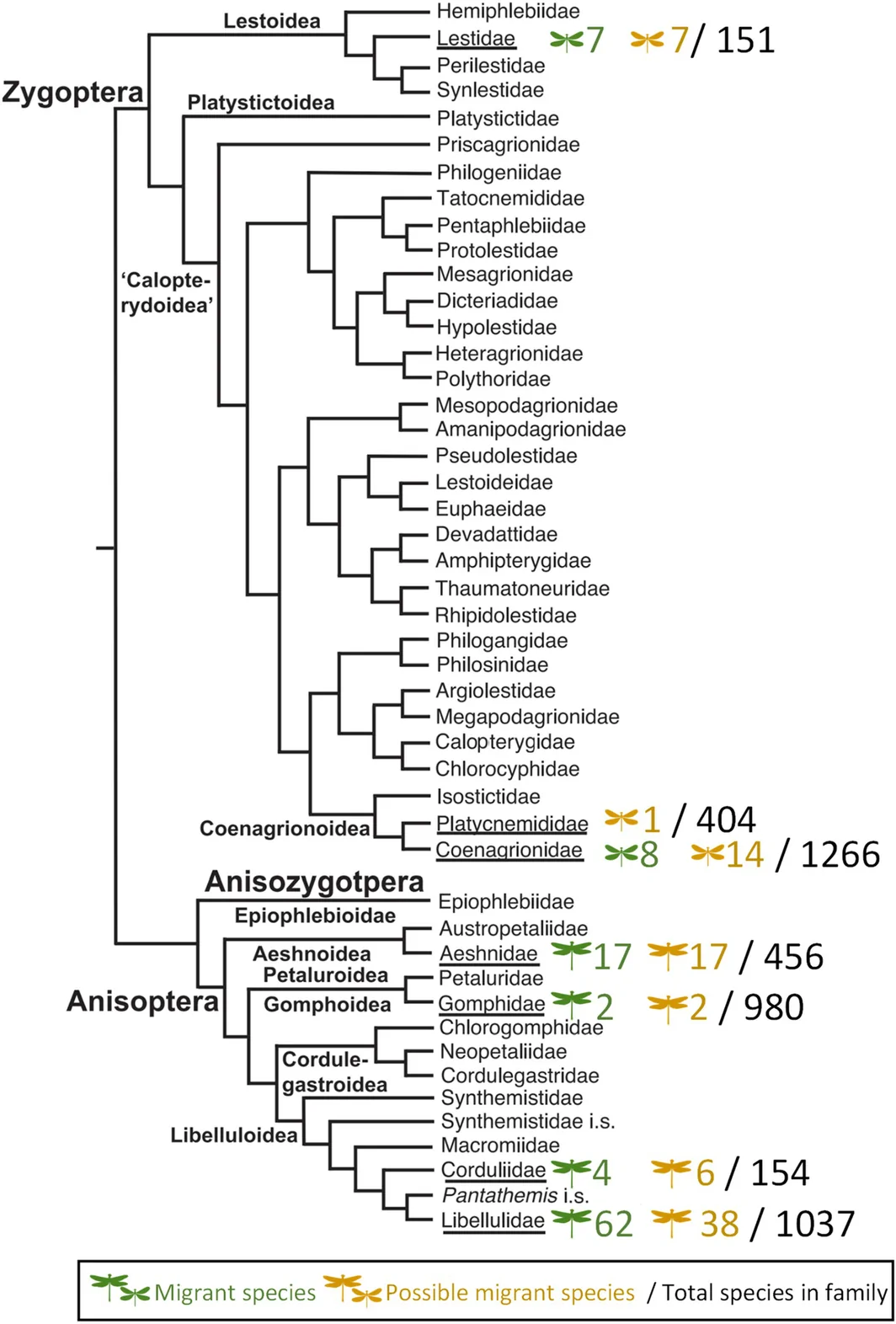
Phylogeny of odonate families within Anisoptera (dragonflies), Zygoptera (damselflies) and Anisozygoptera, with number of confirmed migrant species (green) and possible migrant species (orange) shown. Phylogeny modified from Bybee *et al*. (2021).

In total, 392 publications were consulted to confirm or disprove migratory behaviour, spanning 187 journals (Table S1). The search revealed records confirming migration in 100 odonate species (Table 2; Fig. 2; Dataset S1). This amounts to ~1.5% of all 6,390 described odonate species. There is a clear bias towards migratory behaviour in dragonflies (Anisoptera, 85 confirmed migrant species) *versus* damselflies (Zygoptera, 15 confirmed migrant species). There is confirmed migratory behaviour in four anisopteran families (Aeshnidae, Corduliidae, Gomphidae, Libellulidae) and two zygopteran families (Coenagrionidae, Lestidae) (Table 2; Fig. 2). Among anisopterans, the vast majority (73%) of migratory species occur in the Libellulidae with 62 confirmed migratory species, equating to 5.9% of all libellulids. The Libellulidae is the largest family in Anisoptera, with 1,037 described species (Suhling *et al*., 2015). However, the Gomphidae, a similarly large family with 980 described species (Suhling *et al*., 2015), includes only two confirmed migrants, suggesting that migratory behaviour is particularly concentrated in libellulids. Among the zygopterans, the migratory species are split almost evenly between Lestidae (seven species) and Coenagrionidae (eight species), with the genera *Ischnura* in Coenagrionidae having the most migrants (five species).

**Table 2:** Number of species identified during the literature search classified as confirmed migrants (total 100, of which 18 were also altitudinal refuge migrants) or possible migrants (total 85).

Suborder	Family	Confirmed migrants		Possible migrants
		All	Altitudinal refuge migrants	All
Anisoptera	Libellulidae	62	9	38
Anisoptera	Aeshnidae	17	3	17
Ansioptera	Corduliidae	4	0	6
Ansioptera	Gomphidae	2	1	2
	***Total Anisoptera***	***85***	***13***	***63***
Zygoptera	Coenagrionidae	8	1	14
Zygoptera	Lestidae	7	4	7
Zygoptera	Platycnemididae	0	0	1
	***Total Zygoptera***	***15***	***5***	***22***
**TOTAL**		**100**	**18**	**85**

In addition to the 100 confirmed odonate migrants, we identified possible migratory behaviour in 85 species (63 anisopterans; 22 zygopterans). For these species, evidence for migration is less clear and further research is needed. Taken together, we identified 185 species as possible or confirmed migrants, amounting to 2.9% of odonate species. According to the IUCN *Red List* (IUCN, 2023), a large majority of confirmed (84 of 85 dragonflies, 14 of 15 damselflies) and possible migrants (59/63 dragonflies, 21/22 damselflies) are regarded as species of Least Concern, meaning that they are not designated as currently under threat (Dataset S1). However, a substantial number of these species of Least Concern have population trends that are designated either as ‘unknown’ or ‘decreasing’, meaning that their full population status remains to be investigated or shows a negative trend (Dataset S1).

#### (b) Phylogeny

Odonate families that include species with confirmed migratory behaviour do not constitute a monophyletic clade in either Zygoptera or Anisoptera (Fig. 2; Table 2). Thus, the observed distribution of migratory behaviour across Odonata strongly suggests the repeated evolution of migration in disparate families (rather than the repeated loss of migration in a much larger number of lineages). This pattern is echoed below the family level. For example, with reference to available phylogenies for *Sympetrum* (Pilgrim & Von Dohlen, 2012), Aeshnidae (Clement *et al*., 2022; Von Ellenrieder, 2003), Libellulidae and Corduliidae (Ware, May & Kjer, 2007), confirmed migratory species (Dataset S1) are distributed across the phylogeny within clades composed primarily of species for which there is no evidence of migration.

#### (c) Biogeographical distribution

**Fig. 3:**
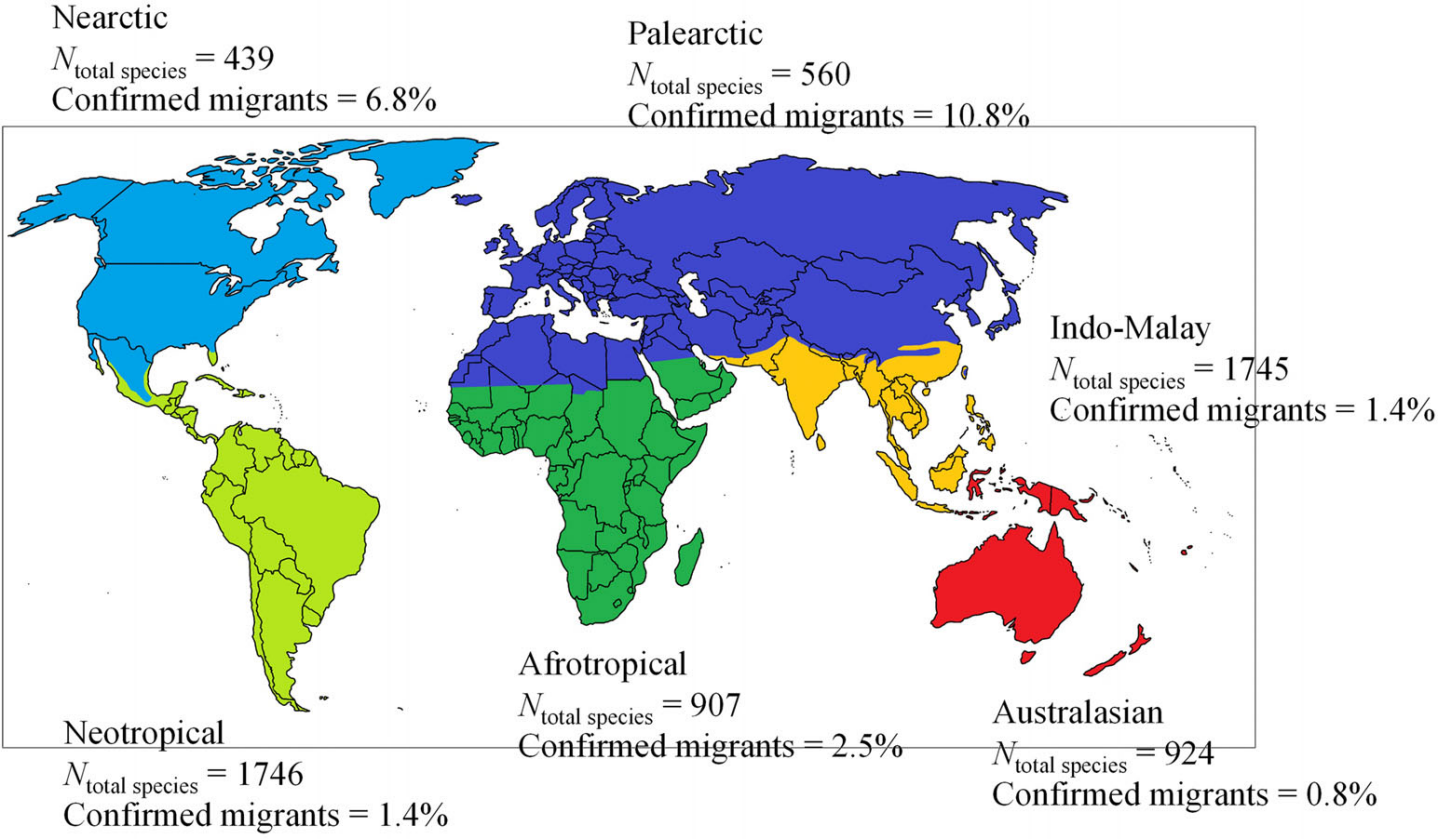
Biogeographic distribution of confirmed migrants (%) in Odonata, where percentage of confirmed migrants is expressed as the proportion of species in each region. Note that several species (Zygoptera *N* = 6, Anisoptera *N* = 47) are present in more than one biogeographic region. See Dataset S1 for additional details of the distribution of individual species.

Of the 6390 known species of Odonata (Paulson *et al*., 2026), 560 species are found in the Palearctic, 439 in the Nearctic, 1746 in the Neotropics (including Galapagos and Hawaii), 1745 in the Indo‐Malay region, 907 in the Afrotropical region, and 924 in the Australasian region (Suhling *et al*., 2015) (Fig. 3). From our compiled studies (see Dataset S1), confirmed migrants make up the largest proportion of species in the Palearctic (10.8%) followed by the Nearctic (6.8%), with the remaining regions all having between 0.8 and 2.5% confirmed migrants (Fig. 3). Notably, several confirmed migrants are present in more than one biogeographical region (Zygoptera *N* = 5; Anisoptera *N* = 47; Dataset S1) and one species (*P. flavescens*), is present in all six regions.

The larger percentage of migrants in the two temperate regions (Nearctic and Palearctic) may indicate that there are more migrant species at high latitudes, a pattern known to exist among birds (Berthold, 2001), but this distribution could also reflect the well‐known bias in research effort favouring the Global North. In particular, numerous odonate species are yet to be described in the Neotropics and Indo‐Malay region (Garrison, Von Ellenrieder & Louton, 2006), and presumably also in the Afrotropics, although this region appears to have comparatively less odonate diversity and is better known taxonomically (Dijkstra, 2007). Alongside taxonomy, improved knowledge of the ecology and behaviour of poorly known species will potentially reveal more instances of migratory behaviour.

#### (d) Altitudinal migration

Altitudinal migration is not often studied in Odonata, and our literature search only found documented evidence for 18 species, 13 dragonflies and five damselflies (Table 2).

A large proportion of these studies were from the Palearctic (76%), and include examples from North Africa, Central Asia and Japan. Less than half of the species (38%) reported to perform altitudinal migrations are also known to engage in multi‐generational migration (Dataset S1) suggesting that the two strategies may share an evolutionary history and/or ecological drivers. In particular, species of the genus *Sympetrum* are well represented amongst the altitudinal migrants (*N* = 7), and three species, *S. meridionale*, *S. sanguineum* and *S. striolatum* are also known to engage in long‐distance, multi‐generational movements (Table S2; Dataset S1).

In most of the species engaging in altitudinal migration (16 out of 18 species), it has been confirmed that sexual maturation is delayed while resident at the high‐altitude site, in the remaining two species it can be assumed that reproduction was paused as no sexual activity was observed (Table S2; *Tholymis tillarga* and *Ceriagrion olivaceum*; Kosterin, 2012).

## V. DISCUSSION

In this review of dragonfly and damselfly migration, we found evidence for two types of migration (multiple‐generational and single‐generational), demonstrated the presence of odonate migrants in six biogeographic regions and confirmed evidence in six of the 46 odonate families, and reviewed current knowledge of odonate migratory adaptations and mechanisms. With *A. junius* and *P. flavescens* as exceptions, a basic understanding of the migration of most odonates is absent. Thus, for the great majority of odonate species, we do not know what elicits migration, how far individuals travel, the phenology of migration, or their migratory routes, origins and destinations. Our lack of knowledge on these fundamental aspects of odonate movement ecology has implications for conservation. For example, the utilisation of multiple breeding sites, or a breeding site and a refuge site, highlights the importance of protecting a range of habitats when designing conservation plans for migratory odonates. We need more detailed information on each species' migratory system and habitat use throughout its whole life history (Chowdhury *et al*., 2023). This review highlights the diversity of migratory behaviours exhibited by odonates, and we hope that it stimulates interest in research on insect migration.

In general, insect migrants are successful species, with wide distributions and are common, likely due to the fact that their life‐history adaptations enable them to follow resource availability (Chapman *et al*., 2015). A propensity to leave the natal site, adaptations to utilise winds and willingness to cross ecological barriers such as oceans, deserts and mountains may also result in a higher probability of establishing metapopulations. For example, in the genus *Ischnura*, four of the five confirmed migrant species (*I. hastata*, *I. pumilio*, *I. ramburii*, *I. senegalensis*) have colonised remote islands (Góral, 2025; Weihrauch, 2011). Only two of the confirmed or possible migrants identified herein are deemed as being near threatened (NT) or endangered (EN) by the IUCN (the St Lucia basker *Urothemis luciana* and the Omani dragonfly *U. thomasi*, Dataset S1), and 107 species are designated as ‘Least Concern’ with a ‘Stable’ or ‘Increasing’ population trend (Dataset S1). Therefore, in Odonata, migration again appears to be a strategy correlated with species success. High mobility of migrant species could be beneficial under climate change; indeed, during recent years range expansion of several tropical migrants into Europe has been recorded (Lohr, 2021; Ott, 2010; Shapoval, Shapoval & Shapoval, 2022). For example, in the 1980s, *A. ephippiger*, a semi‐tropical migrant, was rare even in southern Europe (Silsby, 1993), but since then has become increasingly common in the Mediterranean, and is now a fairly regular breeder in central Europe (Boudot & Kalkman, 2015) and even occasionally in northern Europe (Hoppenbrouwers, 2022). Migrant dragonflies in the Nearctic have also increased in range and prevalence (Ball‐Damerow, M'Gonigle & Resh, 2014). However, global warming represents a threat to many odonates, especially to species not well adapted to higher temperatures (Assandri, 2021; Rosset & Oertli, 2011) and in combination with other drivers, such as land use change, pollution and water abstraction (IUCN, 2021; de Knijf *et al*., 2024). Predicted changes in wind, temperature and rain patterns elicited by climate change may also cause profound disruption to odonate migration, as seen for other insect migrants (Lv *et al*., 2023; Zeng *et al*., 2020), for example by making seasonal wind systems more stochastic or altering their direction and speed (La Sorte *et al*., 2019; Zhang *et al*., 2025).

Insect migration differs from that of vertebrates, particularly because most insect migrants do not complete a round‐trip journey. However, altitudinal migration is an interesting exception and we identified this strategy in 18 species. Considering the short adult lifespan of many odonates, undertaking a considerable geographical relocation, away from and back to the reproductive site, is a remarkable adaptation. By contrast, a recent multi‐order review of altitudinal migration did not identify vertical, seasonal movement in any odonate, and found extremely few insect species in general that exhibit this strategy (Hsiung *et al*., 2018). Clearly, this type of migration is poorly researched in insects. Given that there is evidence that several additional odonate species may seasonally leave their reproductive sites during periods of high temperatures and drought (Corbet, 2004; Paulson *et al*., 2014), more instances of altitudinal migration and single‐generational may yet be discovered. One promising area where such discoveries are likely is the dry forest of Costa Rica, where altitudinal migration in other insect taxa is already known (Hsiung *et al*., 2018; Janzen & Hallwachs, 2021) and where odonate diversity is high (Ramírez, Paulson & Esquivel, 2000). Altitudinal migration in insects is in need of further research, especially as it may have implications for species resilience under environmental change (Inouye *et al*., 2000).

According to our review, only about 1.5–2.9% of odonate species are migratory. Similar percentages have been reported in other insects: ~3% in butterflies (Chowdhury *et al*., 2021), ~3% in European noctuid moths (Alerstam *et al*., 2011), 0.5% in Diptera (but 3% within Syrphidae) (Hawkes *et al*., 2025). A similar percentage was reported for bats (~3%) (Fleming & Eby, 2003). By contrast, the proportion of migrant bird species is ~19% (Somveille *et al*., 2013). In fact, the majority of odonate families lack (confirmed) migratory species: 31 of the 33 families in Zygoptera, and eight of 12 in Anisoptera. Diapause is an alternative to migration in insects, as is hibernation in bats, enabling species to remain in areas that temporarily become unsuitable for reproduction or survival. This strategy is not available to birds, and this could be one reason why migration is a comparatively more common strategy in the avian order.

Considering the distribution of migration across the odonate phylogeny (Fig. 2; Table 2), we consider migration to have evolved independently multiple times. A similar finding was recently reported for the odonate genus *Anax*, where the approximate dates of gains and losses of migration across the phylogeny appeared to correlate with major geological and climatic events, suggesting that migration evolved as a response to a changing environment in this taxon (Clement *et al*., 2022).

Since dragonfly and damselfly migration was last reviewed from a global perspective (Corbet, 2004), several tools have been developed that are promising for future research, particularly wind trajectory models (Cao *et al*., 2018; Hedlund *et al*., 2021; Hobson *et al*., 2021), stable isotope analysis (Hallworth *et al*., 2018; Hobson *et al*., 2012a; Reich *et al*., 2021; Ware *et al*., 2022) and powerful compact radar systems, e.g. the BirdScan‐ MR1 (Haest *et al*., 2024; Nilsson *et al*., 2018; Swiss Birdradar Solution, 2022). In addition, insect movement ecology research would greatly benefit from a large‐scale network of insect observatories, similar to the long‐established bird‐ringing efforts; such programmes could produce a wealth of insights into insect migratory phenology and ecology. Awaiting such initiatives, the increasing popularity of community/citizen science, now enables public data gathering of observations of the natural world that can be used by researchers, for example to track insect movements (Hallworth *et al*., 2018).

Amidst insect declines (Goulson, 2019), the struggle to reduce biodiversity loss (Chowdhury *et al*., 2023), the spread of invasive crop pests (Early *et al*., 2018) and climate change‐induced pest outbreaks (Boulanger *et al*., 2016), understanding the movement ecology of insects is of increasing importance. Odonates play pivotal roles in ecosystems and provide important ecosystem services (Corbet, 2004; May, 2019; Simaika & Samways, 2008), but are subject to anthropogenic threats through habitat degradation (IUCN, 2021) and climate‐induced shifts in phenology and temperature optima (Hassall *et al*., 2007; Novella‐Fernandez *et al*., 2023). Unravelling the biology of odonate migration represents a significant aim for future research efforts.

## VI. CONCLUSIONS

(1) Odonata includes migratory species with diverse ecosystem impacts, some of which perform astonishingly long‐distance and large‐scale migrations, which can comprise thousands or millions of individuals.
(2) Several migratory adaptations have been described in Odonata, relating to wind usage, energy storage and detection of environmental cues. However, the fundamental mechanisms by which odonates achieve their directed migrations remain largely unknown.
(3) Odonate migratory behaviour is of two main types: multi‐generational migration, where a round‐trip migratory journey is completed over several generations, and single‐generational migration, where the same individuals leave and return to the original reproductive habitat. Altitudinal migration was the only form of single‐generational migration we could identify in odonates, and therefore single‐generational migration and altitudinal migration can be regarded as synonymous in odonates. We provide a list of 18 species known to engage in altitudinal migration.
(4) Between 1.5 and 2.9% of odonate species are either confirmed migrants (*N* = 100) or possible migrants (*N* = 85). This low proportion is very similar to that observed in several other animal taxa which are able to diapause or hibernate as an alternative to migration.
(5) The majority of confirmed and possible migrant species are designated as species of Least Concern by the IUCN, indicating that migratory behaviour may buffer odonates against environmental change.
(6) The distribution of migratory behaviour across odonate phylogeny strongly suggests repeated evolution of migration in disparate families.
(7) The Nearctic and Palearctic harbour the highest percentage of migratory odonate species, potentially due to either bias in research efforts, or the ecological benefits of adopting migratory behaviour at higher latitudes.
(8) Migration in Odonata is likely predominantly driven by seasonal unsuitability of reproductive habitats, either due to cold temperatures or a lack of rain. High reproductive density may also elicit migration.
(9) The vast majority of odonate migration biology remains poorly explored. However, technological developments for tracking, simulating, and recording patterns of movement ecology now make it possible to investigate the patterns and processes underlying the remarkable biology involved in insect migration.

## Supporting information


**Dataset S1.** Compilation of odonate species identified as confirmed migrants or possible migrants, the biogeographical region in which they are found, and their IUCN *Red List* status.

**Table S1.** List of 390 sources used to evaluate migratory status (multi‐generational migration and/or altitudinal migration) in odonates.
**Table S2.** List of the 18 odonate species (dragonflies/Anisoptera *N* = 13, damselflies/Zygoptera *N* = 5) designated as altitudinal migrants, and supporting citations.

## Data Availability

The data that supports the findings of this study are available in the supplementary material of this article.
