# Supplementary material for: Flight of the dragons: a global review of migration in Odonata
Source: Biol Rev Camb Philos Soc. 2026 May 29;101(5):2134–57. doi: 10.1002/brv.70170 (PMC13542366; doi:10.1002/brv.70170)
Supplement: Supplementary file 1 [file BRV-101-2134-s001.docx]

## **Table S1.** List of 390 sources used to evaluate migratory status (multi-generational migration and/or altitudinal migration) in odonates. The most common publication language was English (*N* = 330), followed by German (*N* = 22), French (*N* = 15), Japanese (*N* = 4), Russian (*N* = 4), Czech, Dutch, Slovak, and Spanish (*N* = 2 each), and Danish, Finnish, Lithuanian, Polish and Swedish (*N* = 1 each). Books were the most common publication type (*N* = 39), followed by the following five odonate-specialist journals: *Odonatologica* (*N* = 27); *Libellula* and *Libellula Supplement* (*N* = 25); *Argia* (*N* = 18); *Journal of the British Dragonfly Society* (*N* = 15); *International Journal of Odonatology* (*N* = 13). Note that this is not an exhaustive list of all publications available that reference odonate migration, but is the full list of sources used in this study.

| **Species** | **Reference** | **Publication type/journal name** | **Language** |
| --- | --- | --- | --- |
| *Tramea onusta* | Abbott (2007) | Book | English |
| *Tramea binotata* | Abbott (2015) | Book | English |
| *Anax tristis* | Acquah-Lamptey *et al.* (2013) | *Journal of Agriculture* & *Biodiversity Research* | English |
| *Chalcolestes viridis*, *Lestes barbarus* | Agüero-Pelegrin *et al.* (1999) | *Aquatic Insects* | English |
| *Orthetrum trinacria*, *Trithemis annulata*, *Trithemis arteriosa*, *Zygonyx torridus*, *Anax imperator*, *Ischnura senegalensis*, *Pseudagrion glaucescens*, *Lestes pallidus* | Aistleitner *et al.* (2008) | *Mitteilungen Des Internationalen Entomologischen Vereins* | German |
| *Anax amazili* | Alves *et al.* (2019) | *Peer Journal* | English |
| *Lestes virens* | Amari *et al.* (2019) | *Ecological Entomology* | English |
| *Diplacodes trivalis*, *Pantala flavescens*, *Tholymis trivalis*, *Tramea basilaris*, *Tramea limbata*, *Anax guttatus*, *A tristis* | Anderson (2009) | *Journal of Tropical Ecology* | English |
| *Crocothemis erythraea* | Anselin (1984) | *Libellula* | English |
| *Tramea transmarina*, *Hemicordulia australiae*, *Ischnura aurora*, *Aeshna brevistyla*, *Anax papuensis* | Armstrong (1973) | *New Zealand Entomologist* | English |
| *Sympetrum depressiusculum*, *Sympetrum frequens* | Asahina (1984) | *Advances in Odonatology* | English |
| *Tramea virginia* | Asahina & Turuoka (1970) | *Kontyû* | Japanese |
| *Sympetrum depressiusculum*, *Orthetrum albistylum* | Baccetti, Perrotti & Utzeri (1990) | *Notulae Odonatologicae* | English |
| *Brachythemis leucosticta*, *Diplacodes lefebvrii*, *Tholymis tillarga*, *Trithemis arteriosa*, *Trithemis hecate*, *Trithemis werneri*, *Ischnura senegalensis*, *Urothemis assignata*, *Urothemis edwardsii* | Bailey & Litterick (1993) | *Hydrobiologia* | English |
| *Macrodiplax cora* | Balinsky (1967) | *Journal of the Entomological Society of Southern Africa* | English |
| *Macrodiplax cora*, *Tholymis tillarga*, *Agriocnemis pygmaea* | Ball (2014) | *Check List* | English |
| *Leucorrhinia pectoralis* | Barbotte & Ruffoni (2018) | *Revue Scientifi Que Bourgogne-Franche-Comté Nature* | French |
| *Ischnura ramburii* | Bass (2003) | *BioScience* | English |
| *Anax amazali* | Bermúdez & Lopez-Victoria (2009) | *Revista Colombiana de Entomología* | Spanish |
| *Aeshna affinis* | Bernard (2005) | *Notulae odonatologicae* | English |
| *Orthetrum brunneum* | Bernard & Ivinskis (2004) | *Acta Zoologica Lituanica* | English |
| *Sympetrum flaveolum* | Bertram & Haacks (1999) | *Libellula* | English |
| *Somatochlora flavomaculata* | Beukeboom (1988) | *Entomologische berichten Amsterdam* | English |
| *Anax ephippiger*, *Calopteryx virgo* | Beutenmuller (1890) | Book | English |
| *Libellula depressa* | Bitzer (2003) | Report | English |
| *Anax ephippiger* | Boness (2000) | *Atalanta* | English |
| *Miathyria marcella* | Boomsma (1993) | *Occasional Papers Of The Belize Natural History Society* | English |
| *Sympetrum arenicolor*, *Sympecma gobica*, *Sympecma fusca*, *Sympecma paedisca* | Borisov (2006) | *Zoologicheskii Zhurnal* | English |
| *Sympetrum striolatum*, *Sympetrum ariencolor*, *Anax parthenope*, *Sympecma gobica* | Borisov (2009) | *Entomological Review* | English |
| *Sympecma fusca*, *Sympecma paedisca* | Borisov (2010) | *Eurasian Entomological Journal* | Russian |
| *Sympetrum arenicolor* | Borisov (2015*b*) | *Eurasian Entomological Journal* | English |
| *Anax ephippiger* | Borisov (2015*a*) | *Entomological Review* | Russian |
| *Sympetrum fonscolombii* | Borisov *et al.* (2020) | *Ecological Entomology* | English |
| *Sympetrum meridionale*, *Sympetrum striolatum*, *Aeshna mixta*, *Anax parthenope*, *Sympecma gobica*, *Sympecma fusca* | Borisov, Kazenas & Borisov (2022) | *Eurasian Entomological Journal* | English |
| *Tramea lacerate* | Borror (1953) | *Entomological news* | English |
| *Zygonyx torridus*, *Orthetrum chrysostigma* | Boudot (2008) | *Martinia* | French |
| *Sympetrum nigrifemur* | Boudot & Kalkman (2015) | Book | English |
| *Sympetrum flaveolum* | Boudot *et al.* (2009) | *Libellula Supplement* | English |
| *Rhyothemis semihyalina* | Boudot *et al.* (2016) | IUCN Red List report | English |
| *Sympecma paedisca*, *Somatochlora metallica* | Brockhaus (2012) | *Libellula Supplement* | English |
| *Tholymis tillarga* | Brooks & Jackson (2001) | *Odonatologica* | English |
| *Nasiaeschna pentacantha* | Brunelle (1999) | *Northeastern Naturalist* | English |
| *Aeshna affinis*, *Sympetrum meridionale* | Buczyński (1999) | *Acta Hydrobiologica* | English |
| *Aeshna affinis* | Buczyński (2006) | Book section | English |
| *Crocothemis erythraea* | Buczyński & Moroz (2004) | *Notulae odonatologicae* | English |
| *Sympetrum fonscolombii* | Buczyński & Pakulnicka (2000) | *Notulae odonatologicae* | English |
| *Cordulia aenea* | Buczyński, Shapoval & Buczyńska (2014) | *Odonatologica* | English |
| *Erythromma viridulum* | Buczyński *et al.* (2019) | *Odonatrix* | Polish |
| *Macrodiplax cora*, *Diplacodes bipunctata* | Buden (2011) | *Micronesica* | English |
| *Diplacodes bipunctata* | Buden (2018) | *Pacific Science* | English |
| *Anax ephippiger* | Burbach & Winterholler (1997) | *Libellula* | German |
| *Libellula quadrimaculata*, *Aeshna eremita* | Calvert (1893) | *Transactions of the American Entomological Society* | English |
| *Libellula pulchella* | Cannings (2016) | *Argia* | English |
| *Sympetrum depressiusculum*, | Carchini *et al.* (2004) | *Odonatologica* | English |
| *Tramea limbata*, *Rhyothemis variegata*, *Zyxomma petiolatum* | Carr (2020) | *Notulae Odonatologicae* | English |
| *Epitheca cynosura* | Catling (2005) | *Ontatio Odonata* | English |
| *Ischnura hastata* | Catling (2008) | *Argia* | English |
| *Libellula pulchella*, *Pachydiplax longipennis*, *Pantala flavescens*, *Pantala hymenaea*, *Sympetrum corruptum*, *Sympetrum vicinum* | Catling & Brownell (1998) | *Argia* | English |
| *Celithemis eponina*, *Celithemis elisa*, *Libellula semifasciata*, *Libellula vibrans*, *Sympetrum danae*, *Aeshna constricta*, *Aeshna sitchensis*, *Epitheca cynosura* | Catling *et al.* (2017) | *Toronto Entomoloists’ Association, Occasional Publication* | English |
| *Anax ephippiger* | Cedhagen (1988) | *Entomologisk Tidskrift* | English |
| *Ischnura pumilio* | Cham (1991) | *Journal of the British Dragonfly Society* | English |
| *Crocothemis erythraea* | Chari, Moyo & Richoux (2018) | *Ecological Entomology* | English |
| *Tramea lacerate*, *Aeshna verticalis* | Childress (2014) | *Banisteria* | English |
| *Sympetrum striolatum* | Clarke (2005) | Book | English |
| *Diplacodes lefebvrii* | Clausnitzer (2003) | *Odonatologica* | English |
| *Diplacodes lefebvrii*, *Diplacodes lumians*, *Orthetrum trinacria*, *Rhyothemis semihyalina*, *Trithemis annualata*, *Anax tristis*, *Ischnura senegalensis* | Clausnitzer (2005) | *Agrion* | English |
| *Libellula quadrimaculata*, *Pantala flavescens*, *Sympetrum corruptum*, *Tramea basilaris* | Corbet (1962) | PhD thesis | English |
| *Orthemis ferruginea*, *Anax ephippiger* | Corbet (1981) | *Odonatologica* | English |
| *Diplacodes lumians*, *Anax ephippiger*, *Pantala flavescens* | Corbet (1984*a*) | *Odonatologica* | English |
| *Anax papuensis*, *Hemicordulia tau* | Corbet (1984*b*) | *Societas Internationalis Odonatologica Rapid Communications (Supplements]* | English |
| *Hemicordulia australiae*, *Hemicordulia tau*, *Crocothemis erythraea*, *Diplacodes bipunctata*, *Diplacodes lefebvrii*, *Diplacodes lumians*, *D trivalis*, *Miathyria marcella*, *Palpopleura lucia*, *Sympetrum frequens*, *Tramea virginia*, *Zygonyx torridus*, *Anaciaeschna jaspidea*, *Anax amazali*, *Anax gibbosulus*, *Anax guttatus*, *Anax parthenope*, *Anax tristis*, *Anax ephippiger*, *Hemianax papuensis*, *Rhionaeschna bonariensis*, *Agriocnemis exilis*, *Ischnura aurora*, *Ischnura evansi*, *Ischnura hastata*, *Iscnura pumilio*, *Pseudagrion microcephalum*, *Pseudagrion decorum* | Corbet (2004) | Book | English |
| *Orthetrum sabina* | Corbet *et al.* (2006) | *International Journal of Odonatology* | English |
| *Ischnura hastata* | Cordero Rivera *et al.* (2005) | *Odonatologica* | English |
| *Selysiothemis nigra*, *Sympetrum meridionale*, *Trithemis kirbyi*, *Aeshna affinis*, *Anax imperator*, *Anax parthenope*, *Brachythemis impartita*, *Orthetrum brunneum*, *Orthetrum trinacria* | Corso *et al.* (2012) | *Biodiversity Journal* | English |
| *Trithemis kirbyi* | Corso *et al.* (2017) | *Biodiversity Journal* | English |
| *Rhyothemis semihyalina*, *Orthetrum brachimale* | Couteyen & Papazian (2011) | *L'Entomologiste* | French |
| *Diplacodes lefebvrii*, *Paragomphus genei* | Cowan & Cowan (2017) | *Journal of Threatened Taxa* | English |
| *Tramea calverti* | Craves & O’Brien (2011) | *Great Lakes Entomologist* | English |
| *Perithemis tenera* | Crumrine, Switzer & Crowley (2008) | Book | English |
| *Trithemis morrisoni*, *Trithemis palustris* | Damm (2009) | Thesis | English |
| *Trithemis arteriosa*, *Orthetrum chrysostigma* | Damm & Hadrys (2012) | *Organisms Diversity* & *Evolution* | English |
| *Aeshna isoceles* | David & Králiková (2011) | *Životné prostredie* | Slovak |
| *Ischnura senegalensis*, *Sympetrum depressiusculum* | David & Šácha (2019) | *Ochrana prírody, Banská Bystrica* | Slovak |
| *Zyxomma petiolatum* | Dawn (2014) | *Journal of Entomology* & *Zoology Studies* | English |
| *Aeshna isoceles* | D’Aguilar, Dommanget & Prechac (1986) | Book | English |
| *Aeshna affinis* | De Knijf, Anselin & Goffart (2002) | *Bulletin van het Koninklijk Belgisch Instituut voor Natuurwetenschappen. Biologie = Bulletin de l'Institut Royal des Sciences Naturelles de Belgique.* | English |
| *Lestes barbarus* | De Knijf & Anselin (2010) | *BioRisk* | English |
| *Zygonyx torridus*, *Paragomphus genei* | De Knijf & Demolder (2010) | *Libellula* | English |
| *Selysiothemis nigra*, *Chalcolestes viridis*, *Chalcolestes parvidens*, | De Knijf & Demolder (2013) | *Libellula* | English |
| *Gynacantha nervosa* | De Marmels (1994) | *Odonatologica* | English |
| *Tramea rustica* | De Marmels & Rácenis (1982) | *Odonatologica* | English |
| *Anax amazili*, *Pantala hymenaea*, *Tramea calverti* | De Marmels, José & Sharpe (2008) | *Argia* | English |
| *Agriocnemis exilis* | de Santos Loureiro (2013) | *Zoologia Caboverdiana* | English |
| *Ischnura senegalensis* | de Santos Loureiro & Martins (2016) | *African Entomology* | English |
| *Tramea insularis* | Deviche & Jones (2016) | *Argia* | English |
| *Orthetrum brunneum*, *Orthetrum coerulescens* | Devillers *et al.* (2019) | *Les Naturalistes belges* | French |
| *Selysiothemis nigra* | Dia & Dumont (2011) | *Zoology in the Middle East* | English |
| *Brachythemis impartita* | Diaz-Paniagua *et al.* (2014) | *Boleín Rola* | English |
| *Hemicordulia australiae*, *Hemicordulia tau*, *H asiatica* | Dijkstra (2007) | *Zootaxa* | English |
| *Lindenia tretraphylla*, *Sympetrum corruptum* | Dijkstra & Kalkman (2012) | *Organisms Diversity* and *Evolution* | English |
| *Zygonyx torridus*, *Aeshna affinis*, *A mixta*, *Sympetrum sinaiticum* | Dijkstra & Lewington (2006) | Book | English |
| *Diplacodes bipunctata* | Dijkstra (2006) | *International Journal of Odonatology* | English |
| *Anax guttatus*, *Tramea transmarina*, *Ischnura aurora* | Donnelly (2005) | *Argia* | English |
| *Lestes australis* | Dubois & Tenessen (2021) | *The Great Lakes Entomologist* | English |
| *Anax longipes* | DuBois & Van den Broek (2016) | *Argia* | English |
| *Crocothemis erythraea* | Dumont (1967) | *Biologisch Jaarboek* | English |
| *Pseudagrion sublacteum* | Dumont (1973) | *Israel Journal of Zoology* | English |
| *Anax ephippiger*, *Tramea basilaris* | Dumont (1977) | *Odonatologica* | English |
| *Tholymis tillarga*, *Olpogastra lugubris*, *Orthetrum brachimale*, *Agriocnemis exilis*, *Agriocnemis zerafica* | Dumont (2004) | *Bulletin Société royale belge d'Entomologie Koninklijke Belgische Vereniging* | English |
| *Libellula vibrans* | Dunkle (1988) | *Notulae Odonatologicae* | English |
| *Aeshna verticalis* | Dunkle (2000) | Book | English |
| *Erythrodiplax berenice* | Dunson (1980) | *Physiological Zoology* | English |
| *Sympetrum meridionale*, *Aeshna mixta* | Dyatlova & Kalkman (2008) | *Entomologische Berichten* | English |
| *Zygonyx torridus* | Ebrahimi, Mohammadian & Madjdzadeh (2014) | *Journal of the International Dragonfly Fund* | English |
| *Aeshna affinis*, *Ophiogomphus cecilia*, *Lestes barbarus*, *Lestes virens*, *Sympetrum pedemontanum*, *Orthetrum brunneum* | Edelaar, Dijkstra & Dingemanse (1996) | *Entomologische berichten Amsterdam* | English |
| *Sympetrum danae* | Eimer (1882) | *Jahreshefte des Vereins für Vaterländische Naturkunde in Württemberg* | German |
| *Sympetrum sinaiticum* | El Haissoufi *et al.* (2015) | *Odonatologica* | English |
| *Macrodiplax cora*, *Orthetrum brachimale*, *Orthetrum stemmale*, *Palpopleura lucia* | Élouard, Gibon & Gattolliat (2000) | Book | French |
| *Paltothemis lineatipes* | Estrada (2018) | Thesis | Spanish |
| *Tramea loewii*, *Anax papuensis* | Farrow (1984) | *Australian Journal of Ecology* | English |
| *Libellula quadrimaculata*, *Sympetrum danae*, *Sympetrum flaveolum* | Federley (1908) | *Acta Societatis pro Fauna et Flora Fennica* | German |
| *Pantala flavescens*, *Anax parthenope* | Feng *et al.* (2006) | *Ecological Entomology* | English |
| *Anax imperator*, *Orthetrum coerulescens* | Ferreira *et al.* (2006) | *International Journal of Odonatology* | English |
| *Selysiothemis nigra*, *Orthetrum taeniolatum*, *Paragomphus genei* | Feulner, Reimer & Hornby (2007) | *Tribulus* | English |
| *Lestes macrostigma* | Flint (2019) | *Libellula* | English |
| *Lindenia tetraphylla* | Fontana-Bria *et al.* (2017) | *Arxius de Miscel·lània Zoològica* | English |
| *Ischnura pumilo* | Fox (1989) | *The entomologist's record and journal of variation.* | English |
| *Tholymis tillarga*, *Diplacodes trivialis* | Fraser (1918) | *The journal of the Bombay Natural History Society* | English |
| *Lestes elata*, *Lestes praemorsus* | Fraser (1924*a*) | *Records of the Indian Museum* | English |
| *Zyxomma petiolatum* | Fraser (1924*b*) | *Records of the Zoological Survey of India* | English |
| *Lestes malabaricus* | Fraser (1931) | *Records of the Indian Museum* | English |
| *Pseudagrion decorum*, *Pseudagrion microcephalum*, *Aciagrion occidentale*, *Onychargia atrocyana*, *Pantala flavescens*, *Anax ephippiger* | Fraser (1933) | Book | English |
| *Orthetrum trinacria*, *Lindenia tetraphylla*, *Selysiothemis nigra*, *Diplacodes trivalis* | Fraser (1936) | Book | English |
| *Hemicordulia asiatica* | Fraser (1949) | *Proceedings of the Royal Entomological Society London (B)* | English |
| *Crocothemis erythraea*, *Macrodiplax cora*, *Orthetrum brachiale*, *Orthetrum stemmale*, *Orthetrum trinacria*, *Palpopleura lucia*, *Tholymis tillarga*, *Anax imperator* | Fraser (1956) | Book | French |
| *Anax junius* | Freeland *et al.* (2003) | *Ecological Entomology* | English |
| *Anax guttatus* | Futahashi (2017) | *Bulletin of Ibaraki Natural Museum* | Japanese |
| *Lestes pallidus*, *Tramea basilaris*, *Diplacodes lumians*, *Pantala flavescens*, *Anax ephippiger*, *Anax imperator* | Gambles (1960) | *Journal of the West African Science Association* | English |
| *Tramea basilaris*, *Trithemis pallidinervis*, *Anax tristis*, *Selysiothemis nigra*, *Lindenia tetraphylla* | García *et al.* (2015) | IUCN Red List | English |
| *Triacanthagyna trifida* | García-Díaz (1938) | *The journal of agriculture of the University of Puerto Rico* | English |
| *Sympetrum vulgatum*, *Sympetrum flaveolum* | Gatter (1975) | *Atalanta* | German |
| *Anax parthenope*, *Selysiothemis nigra* | Gauci (2014) | *Journal of the British Dragonfly Society* | English |
| *Somatochlora flavomaculata* | Gauci (2019) | *Journal of the British Dragonfly Society* | English |
| *Zyxomma petiolatum* | Global Core Biodata Resource (GBIF) (2023) | *Global Core Biodata Resource (GBIF) online species web page on Zyxomma petiolatum* | English |
| *Anax amazili*, *Erythrodiplax berenice*, *Erythrodiplax umbrata* | Geijskes (1967) | *Entomologische Berichten* | German |
| *Anax guttatus* | Gibson-Hill (1950) | *The Bulletin of the Raffles Museum* | English |
| *Anax ephippiger*, *Lindenia tetraphylla*, *Selysiothemis nigra*, *Urothemis thomasi* | Giles (1998) | *Tribulus* | English |
| *Ischnura hastata*, *Libellula vibrans*, *Perithemis tenera*, *Pachydiplax longipennis* | Glick (1939) | *Technical bulletin of the United States Department of Agriculture* | English |
| *Leucorrhinia pectoralis* | Goffart, Motte & V&evyvre (2012) | *Les Naturalistes belges* | French |
| *Lestes australis* | Góral (2025) | *Odonatologica* | English |
| *Sympetrum corruptum* | Gonzáles-Soriano (2008) | *Notulae odonatologicae* | English |
| *Orthetrum sabina* | Govindan, Ramanibai & Balakrishnan (2015) | *International Journal of Multidisciplinary Research and Development* | English |
| *Aeshna mixta* | Goyaud (2001) | *Revue annuelle de l’association Les Naturalistes Vendéens* | French |
| *Ischnura aurora*, *Anax papuensis*, *Hemicodulia australiae*, *Tramea loewi*, *Tramea transmarina* | Grainger *et al.* (2018) | *New Zealand Threat Classification Series 28* | English |
| *Sympetrum meridionale*, *Aeshna affinis* | Grand (2009) | *Revue Scientifique Bourgogne-Nature* | French |
| *Ischnura aurora*, *Zyxomma petiolatum*, *Anaciaeschna jaspidea*, *Anax paupensis*, *Diplacodes trivialis* | Grand *et al.* (2019) | *Zootaxa* | English |
| *Anax junius* | Hallworth *et al.* (2018) | *Biology Letters* | English |
| *Aeshna mixta* | Hämäläinen, Huldén & Karjalainen (2004) | *Sahlbergia* | Finish |
| *Sympetrum fonscolombii* | Hančíková (2011) | Thesis | Czech |
| *Sympecma gobica* | Harabiš, Dolný & Šipoš (2012) | *Population Ecology* | English |
| *Sympecma fusca*, *Ischnura elegans* | Haring *et al.* (2020) | *Acta ZooBot Austria* | English |
| *Libellula quadrimaculata*, *Leucorrhinia pectoralis*, *Leucorrhinia rubicunda* | Haritonov & Popova (2011) | *International Journal of Odonatology* | English |
| *Tholymis tillarga*, *Tramea transmarina*, *Tramea virginia*, *Pantala flavescens*, *Anax guttatus* | Hashimoto & Asahina (1969) | *Kontyû* | Japanese |
| *Anax paupensis* | Hawking & New (2002) | *Journal of Insect Conservation* | English |
| *Sympetrum striolatum* | Haynes & Hillis (1985) | *Irish Naturalists' Journal* | English |
| *Anax ephippiger* | Hedlund *et al.* (2020) | *International Journal of Odonatology* | English |
| *Pantala flavescens* | Hedlund *et al.* (2021) | *Frontiers in Ecology and Evolution* | English |
| *Ischnura aurora* | Hilfert-Rüppell (2004) | Thesis | German |
| *Pantala flavescens* | Hobson *et al.* (2012*a*) | *PLoS ONE* | English |
| *Anax junius* | Hobson *et al.* (2012*b*) | *Methods in Ecology and Evolution* | English |
| *Pantala flavescens* | Hobson *et al.* (2021) | *Environmental Entomology* | English |
| *Rhionaeschna marchali* | Hoffmann (2010) | *BioRisk* | English |
| *Epiaeschna heros*, *Anax junius* | Holland, Wikelski & Wilcove (2006) | *Science* | English |
| *Somatochlora meridionalis* | Holuša (2009) | *Acta Musei Beskidensis* | English |
| *Selysiothemis nigra* | Holuša (2011) | *Libellula* | English |
| *Orthetrum brunneum* | Holuša (2010) | *Acta Musei Beskidensis* | Czech |
| *Rhionaeschna bonariensis* | Hudson (1895) | Book | English |
| *Orthetrum trinacria*, *Trithemis arteriosa* | Hupalo & Tończyk (2014) | *Acta Zoologica Bulgarica* | English |
| *Leucorrhinia dubia* | Imms (1900) | *The Entomologist's Monthly Magazine* | English |
| *Rhionaeschna bonariensis* | Jaramillo (1993) | *The Condor* | Englis |
| *Sympetrum sinaiticum* | Jödicke (1995) | *Odonatologica* | German |
| *Orthemis ferruginea*, *Sympetrum corruptum*, *Tramea onusta* | Johansson, Söderquist & Bokma (2009) | *Biological Journal of the Linnean Society* | English |
| *Libellula quadrimaculata*, *Libellula depressa*, *Leucorrhinia pectoralis*, *Leucorrhinia rubicunda*, *Aeshna juncea*, *Somatochlora metallic*, *Cordulia aenea* | Johnson (1969) | Book | English |
| *Aeshna affinis*, *Anax parthenope*, *Anax ephippiger*, *Crocothemis erythraea*, *Orthetrum brunneum*, *Sympetrum depressiusculum*, *Sympetrum fonscolombii*, *Sympetrum meridionale*, *Sympetrum pedemontanum* | Kalniņš (2009) | *Latvijas Entomologs* | English |
| *Sympetrum pedemontanum* | Kalkman (2014) | IUCN Red List report | English |
| *Lindenia tetraphylla*, *Selysiothemis nigra* | Katbeh-Bader *et al.* (2004) | *Denisia* |  |
| *Sympecma fusca* | Kathan (2019) | Thesis | German |
| *Libellula quadrimaculata*, *Leucorrhinia pectoralis*, *Leucorrhinia rubicunda*, *Sympetrum danae* | Kharitonov & Popova (2011) | *Entomological Review* | English |
| *Lindenia tetraphylla* | Khelifa & Zebsa (2018) | *Notulae odonatologicae* | English |
| *Erythemis simplicicollis*, *Tramea lacerata* | Kielb (1996) | *Great Lakes Entomologist* | English |
| *Anax longipes* | Kielb & O’Brien (1996) | *Great Lakes Entomologist* | English |
| *Sympetrum corruptum* | King (2011) | *Argia* | English |
| *Erythromma viridulum* | Knight (2007) | *Sawfly Study Group Newsletter* | English |
| *Aeshna mixta*, *Sympetrum sanguineum*, *Sympetrum vulgatum* | Knoblauch, Thoma & Menz (2021) | *Animal Behaviour* | English |
| *Trithemis annulata*, *Trithemis kirbyi* | Koch & Suhling (2005) | *Canadian Journal of Zoology* | English |
| *Somatochlora sahlbergi* | Kohli *et al.* (2018) | *Scientific Reports* | English |
| *Sympetrum nigrifemur* | Kormondy (1961) | *Journal of the New York Entomological Society* | English |
| *Potamarcha congener*, *Rhyothemis variegata* | Kosterin (2012) | *Cambodian Journal of Natural History* | English |
| *Sympecma gobica*, *Sympecma fusca*, *Sympecma paedisca* | Kosterin & Borisov (2010) | *Eurasian Entomological Journal* | Russian |
| *Libellula depressa* | Kosterin & Onishko (2021) | *Eurasian Entomological Journal* | English |
| *Libellula quadrimaculata* | Kosterin, Haritonov & Inoue (2001) | *Sympetrum hyogo* | Japanese/ English |
| *Sympetrum flaveolum* | Kulijer (2015) | *Libellula* | English |
| *Aciagrion occidentale*, *Pseudagrion decorum* | Kulkarni & Prasad (2002) | Book | English |
| *Tramea basilaris*, *Pantala flavescens* | Kumar (1972) | *Odonatologica* | English |
| *Zygonyx torridus* | Kunz, Ober & Jödicke (2006) | *Libellula* | English |
| *Sympetrum striolatum* | Lack & Lack (1951) | *Journal of Animal Ecology* | English |
| *Tholymis tillarga*, *Tramea basilaris*, *Lindenia tetraphylla*, *Macrodiplax cora*, *Agrionemis pygmaea* | Lambret *et al.* (2017) | *Odonatologica* | English |
| *Anax ephippiger* | Lambret & Boudot (2013) | *Martinia* | French |
| *Crocothemis erythraea* | Landmann (1983) | *Berichte des naturwissenschaftlich-medizinischen Vereins in Innsbruck* | German |
| *Orthemis ferruginea* | LeGrand (2022) | Book | English |
| *Aeshna canadensis*, *Anax junius* | Lewis (2020) | *Northeastern Naturalist* | English |
| *Sympetrum meridionale* | Lichtenstein & Grassé (1922) | *Bulletin de la Société entomologique de France* | French |
| *Tholymis tillarga*, *Tramea transmarina*, *Tramea virgina*, *Trithemis pallidinervis*, *Anaciaeschna jaspidea*, *Anax guttatus*, | Lieftnick (1954) | *Treubia* | English |
| *Selysiothemis nigra* | Lohr (2021) | *Libellula* | English |
| *Sympetrum nigrifemur*, *Sympetrum striolatum* | Longfield (1948) | *The Irish Naturalists' Journal* | English |
| *Ischnura hastata* | Lorenzo-Carballa, Cordero-Rivera & Andrés (2010) | Book | English |
| *Leucorrhinia pectoralis* | Lorthiois *et al.* (2012) | *L’Entomologiste Haut-Normand* | French |
| *Libellula pulchella* | Lucas & Brunelle (2016) | *Argia* | English |
| *Sympetrum eroticum*, *Sympetrum frequens*, *Sympetrum speciosum*, *Sympetrum fonscolombii*, *Sympetrum depressiusculum* | Ma *et al.* (2022) | *Taiwanese Journal of Entomological Studies* | English |
| *Aeshna juncea*, *Crocothemis erythraea*, *Lestes barbarus* | Maiolini & Carolli (2009) | *Studi Trentini in Scienze Naturali* | English |
| *Celithemis eponina*, *Ishnura hastata* | Makepeace *et al.* (2017) | *Journal of the Acadian Entomological Society* | English |
| *Sympetrum fonscolombii* | Malkmus & Ruf (2008) | *Libellula* | German |
| *Sympecma paedisca* | Manger & Dingemanse (2009) | *Odonatologica* | English |
| *Sympetrum meridionale*, *Lestes macrostigma* | Marinov (2007) | *Notulae odonatologicae* | English |
| *Hemicordulia australiae* | Marinov (2010) | *The Weta* | English |
| *Anax* spp., *Diplacodes* spp., *Tramea* spp. | Marinov (2015) | *Journal of the International Dragonfly Fund* | English |
| *Orthetrum brachimale*, *Tramea basilaris*, *Pantala flavescens*, *Zygonyx torridus*, *Diplacodes lumians*, *Tholymis tillarga*, *Anax ephippiger* | Marshall & Gambles (1977) | *Journal of Zoology, London* | English |
| *Anax rutherfordi*, *A tristis* | Martens, de Santos Loureio & Hazevoet (2013) | *Zoologia Caboverdiana* | English |
| *Erythrodiplax funerea*, *E umbrata*, *Anax junius*, *Tramea lacerata*, *Tramea calverti*, *Tramea basilaris*, *Pantala flavescens*, *Pantala hymenaea*, *Sympetrum corruptum*, *Epiaeschna heros* | May (2013) | *Journal of Insect Conservation* | English |
| *Anax papuensis*, *Ischnura aurora*, *Hemicodulia australiae* | McGlone (2006) | Book | English |
| *Anax ephippiger*, *Sympetrum fonscolombii* | Mediani *et al.* (2012) | *International Journal of Odonatology* | English |
| *Crocothemis erythraea*, *Sympetrum pedemontanum*, *Sympetrum danae*, *Sympetrum sanguineum*, *Sympetrum striolatum*, *Sympetrum flaveolum*, *Sympetrum vulgatum* | Mendel & Marsh (1996) | *Transactions of the Suffolk Naturalists' Society* | English |
| *Tramea basilaris* | Meurgey (2008) | *Argia* | English |
| *Tramea basilaris* | Meurgey (2012) | *Argia* | English |
| *Tramea binotata*, *T insularis* | Meurgey *et al.* (2012) | Report | French |
| *Sympetrum danae* | Michiels & Dhont (1991) | *Ecological Entomology* | English |
| *Rhionaeschna confusa* | Mielewczyk (1978) | *Notulae odonatologicae* | English |
| *Potamarcha congener* | Miller (1989) | *Odonatologica* | English |
| *Anax imperator* | Minot & Husté (2022) | *Diversity* | English |
| *Pantala flavescens* | Mitra (1974) | *The Entomologist's Record and Journal of Variation* | English |
| *Aciagrion pallidum*, *Ceriagrion olivaceum* | Mitra (1999) | *Records of the Zoological Survey of India* | English |
| *Sympetrum frequens* | Miyakawa (1994) | *Odonatologica* | English |
| *Sympetrum meridionale*, *Sympetrum pedemontanum*, *Sympetrum depressiusculum* | Monnerat (2002) | *Bulletin Romand d'Entemologie* | French |
| *Tramea lacerata*, *Anax junius* | Moskowitz *et al.* (2001) | *Northeastern Naturalist* | English |
| *Epiaeschna heros* | Mundt (1882) | *The Canadian Entomologist* | English |
| *Sympetrum flaveolum* | Murdoch (1998) | *Journal of the British Dragonfly Society* | English |
| *Pseudagrion microcephalum* | Nair (2011) | Report | English |
| *Anax longipes* | Natural Heritage & Endangered Species Program (2015) | Report | English |
| *Anax parthenope*, *Anax imperator* | Nelson, Ronayne & Thompson (2003) | *Irish Naturalists' Journal* | English |
| *Anax imperator* | Nielsen (1994) | *Entomologiske Meddelelser* | Danish |
| *Sympetrum corruptum* | Nikula, Sones & Trimble (2001) | *Northeastern Naturalist* | English |
| *Gomphaeschna antelope* | Nikula, Loose & Burne (2003) | Book | English |
| *Cordulia aenea* | Norman (1957) | *Notulae entomologicae* | Swedish |
| *lschnura pumilio* | Olberg & Lønnve (2012) | *Norwegian Journal of Entomology* | English |
| *Orthetrum sabina* | Olsvik & Hämäläinen (1992) | *Opuscula Zoologica Fluminensia* | English |
| *Sympetrum meridionale* | Onyango-Odiy (1973) | *Entomologists monthly magazine* | English |
| *Crocothemis erythraea* | Ott (1988) | *Libellula* | German |
| *Lestes virgatus*, *Urothemis luciana* | Ott & Samways (2010) | Book section | English |
| *Erythrodiplax umbrata*, *Miathyria marcella*, *Pantala flavescens* | Palacino & Millán (2010) | *Argia* | English |
| *Sympetrum danae*, *Sympetrum flaveolum*, *Sympetrum pedemontanum*, *Anax imperator*, *Leucorrhinia dubia*, *Leucorrhinia pectoralis*, *Leucorrhinia rubicunda*, *Aeshna mixta*, *Aeshna affinis* | Parr (1996) | *Journal of the British Dragonfly Society* | English |
| *Aeshna mixta*, *Anax imperator*, *Libellula depressa*, *Libellula quadrimaculata*, | Parr (1997) | *Journal of the British Dragonfly Society* | English |
| *Lestes barbarus* | Parr (2000) | *NVL-Nieuwsbrief* | English |
| *Erythromma viridulum*, *Cordulia aenea*, *Crocothemis erythraea*, *Sympetrum fonscolombii*, *Anax parthenope* | Parr (2001) | *Journal of the British Dragonfly Society* | English |
| *Lestes barbarus*, *Ischnura pumilio*, *Erythromma viridulum* | Parr (2010) | Conference proceedings | English |
| *Leucorrhinia dubia* | Parr (2013) | *Journal of the British Dragonfly Society* | English |
| *Sympetrum danae*, *Aeshna mixta* | Parr (2014) | *Journal of the British Dragonfly Society* | English |
| *Sympetrum corruptum* | Paulson (1966) | PhD Thesis | English |
| *Tholymis citrina*, *Tramea calverti* | Paulson (2001) | *International Journal of Odonatology* | English |
| *Pantala hymenaea*, *Erythemis vesiculosa*, *Pantala flavescens*, *Tramea onusta*, *Brachymesia herbida*, *Erythrodiplax funereal*, *Orthemis ferruginea*, *Miathyria marcella*, *M simplex* | Paulson (2002) | *Odonatologica* | English |
| *Tholymis citrina*, *Tramea lacerata*, *Rhionaeshna multicolor*, *Anax junius*, *Libellula quadrimaculata*, *Erythrodiplax umbrata*, *Sympetrum corruptum*, *Pantala flavescens*, *Pantala hymenaea* | Paulson (2009) | Book | English |
| *Libellula quadrimaculata*, *Libellula pulchella*, *Libellula semifasciata* | Paulson (2012) | Book | English |
| *Libellula pulchella*, *Libellula semifasciata*, *Libellula vibrans*, *Pachydiplax longipennis*, *Pantala hymenaea*, *Pantala flavescens*, *Sympetrum vicinum*, *Tramea calverti*, *Tramea carolina*, *Tramea lacerata*, *Tramea onusta*, *Epiaeschna heros*, *Gomphaeschna antelope*, *Erythemis vesiculosa*, *Erythrodiplax umbrata* | Paulson *et al.* (2016) | Report | English |
| *Brachymesia herbida* | Peck (1994) | *Annals of the Entomological Society of America* | English |
| *Aeshna isoceles* | Pickwell *et al.* (2012) | *Journal of the British Dragonfly Society* | English |
| *Anax tristis* | Pinhey (1951) | *Transvaal Museum Memoirs* | English |
| *Anax imperator* | Pinhey (1974) | *Bonner zoologische Beiträge* | English |
| *Hemicordulia asiatica*, *Agriocnemis exilis* | Pinhey (1978) | Book section | English |
| *Macrodiplax cora*, *Hemicordulia asiatica*, *Pantala flavescens*, *Tholymis tillarga*, *Tramea basilaris*, *Tramea limbata*, *Anax imperator*, *Anax speratus*, | Pinhey (1985) | *Journal of the Entomological Society of Southern Africa* | English |
| *Sympetrum danae*, *Sympetrum flaveolum*, *Sympetrum vulgatum* | Pittman (1996) | *Journal of the British Dragonfly Society* | English |
| *Trithemis kirbyi* | Polette *et al.* (2019) | *Martinia* | French |
| *Leucorrhinia rubicunda*, *Sympetrum danae* | Popova & Eremina (2016) | *Euroasian Entomological Journal* | Russian |
| *Sympetrum danae*, *Sympetrum vulgatum* | Popova & Haritonov (2014) | *Entomological Review* | English |
| *Orthemis ferruginea*, *Sympetrum corruptum* | Prather & Prather (2015) | Book | English |
| *Erythromma viridulum*, *Orthetrum albistylum* | Račkauskaitė & Gliwa (2015) | *New and rare for Lithuania insect species* | English |
| *Tramea onusta*, *Ischnura hastata* | Reece & Mcintyre (2009) | *Insect Conservation and Diversity* | English |
| *Diplacodes trivalis*, *Orthetrum sabina*, *Rhyothemis variegata* | Reels (2011) | *International Journal of Odonatology* | English |
| *Sympetrum fonscolombii*, *Trithemis pallidinervis* | Reels (2019) | *Journal of the International Dragonfly Fund* | English |
| *Rhionaeschna bonariensis* | Reichholf (1973) | *Odonatologica* | English |
| *Macrodiplax cora* | Reimer, Feulner & Hornby (2009) | *Tribulus* | English |
| *Aeshna juncea* | Rimantas Stonis *et al*. (2009) | *Naujos ir retos lietuvos vabdziu rusys* | Lithuanian |
| *Rhionaeschna confusa* | Ris (1904) | *Ergebnisse der Hamburger Magalhaenischen Sammelreise* | German |
| *Gomphaeschna antelope*, *Gomphaeschna furcillata* | Roble (2005) | *Argia* | English |
| *Erythemis simplicicollis*, *Sympetrum vicinum*, *S corruptum*, *Anax junitts*, *Aeshna constricta*, *Tramea lacerata*, *Tramea carolina*, *Pantala hymenaea*, *Pachydiplax longipennis*, *Libellula pulchella* | Root (1910) | *The Canadian Entomologist* | English |
| *Pantala flavescens*, *Tholymis tillarga*, *Diplacodes bipunctata* | Rosario *et al.* (2011) | *Journal of General Virology* | English |
| *Anax longipes* | Rothfels (2007) | *Ontario Odonata* | English |
| *Ischnura aurora*, *Ischnura elegans* | Rowe (1978) | *Odonatologica* | English |
| *Tramea transmarina* | Rowe (1980) | *New Zealand Entomologist* | English |
| *Ischnura senegalensis*, *Orthetrum sabina*, *Diplacodes trivialis*, *Trithemis pallidinervis*, *Tramea basilaris*, *Tramea limbata*, *Tholymis tillarga* | Ruddek (1998) | *Notulae odonatologicae* | English |
| *Rhionaeschna bonariensis* | Rudolph & Fisher (1993) | *The Wilson bulletin (Wilson Ornithological Society)* | English |
| *Sympecma paedisca* | Ruiter *et al.* (2007) | *Brachytron* | Dutch |
| *Ischnura ramburii*, *Anax amazili*, *A junius*, *Erythemis vesiculosa*, *Erythrodiplax umbrata*, *Pachydiplax longipennis*, *Pantala flavescens*, *Pantala hymenaea*, *Sympetrum corruptum*, *Tramea lacerata*, *Tramea carolina*, *Tramea onusta*, *Miathyria marcella* | Russell (2005) | Report | English |
| *Anax junius*, *Epiaeschna heros*, *Libellula pulchella*, *Libellula semifasciata*, *Pantala flavescens*, *Pantala hymenaea*, *Tramea carolina*, *Tramea lacerata*, *Sympetrum corruptum*, *Aeshna constricta*, *Aeshna eremita*, *Celithemis elisa*, *Erythrodiplax berenice*, *Libellula quadrimaculata*, *Sympetrum rubicundulum* | Russell *et al.* (1998) | *The American Midland Naturalist* | English |
| *Diplacodes bipunctata* | Sakagami (1961) | *Pacific Science* | English |
| *Lestes numidicus*, *Sympecma fusca* | Samraoui (2009) | *International journal of Odonatology* | English |
| *Sympecma fusca*, *Aeshna mixta*, *Lestes virens*, *Sympetrum meridionale*, *Sympetrum striolatum*, *Anax imperator*, *Anax parthenope* | Samraoui & Corbet (2000) | *International Journal of Odonatology* | English |
| *Aeshna mixta* | Samraoui *et al.* (1998) | *International Journal of Odonatology* | English |
| *Anax tristis*, *Macrodiplax cora*, *Tramea limbata*, *Urothemis luciana* | Samways (1999) | *Odonatologica* | English |
| *Zyxomma petiolatum*, *Rhyothemis semihyalina* | Samways (2003) | *Odonatologica* | English |
| *Rhyothemis semihyalina*, *Sympetrum fonscolombii*, *Diplacodes luminans*, *Urothemis edwardsii* | Samways (2010*a*) | *BioRisk* | English |
| *Zyxomma petiolatum* | Samways (2010*b*) | *Phelsuma* | English |
| *Hemicordulia asiatica*, *Hemicordula australiae*, *Hemicordulia tau* | Sánchez-Herrera & Ware (2012) | Book section | English |
| *Libellula quadrimaculata* | Schaub (1997) | *Libellula* | German |
| *Orthetrum chrysostigma*, *Trithemis kirbyi*, *Trithemis annulata*, *Crocothemis erythraea* | Schenk, Suhling & Martens (2004) | *Animal Behaviour* | English |
| *Lestes barbarus* | Schiel & Kunz (2005) | *Libellula* | German |
| *Aeshna canadensis* | Schilling Gaenzle *et al.* (2021) | *Ecological Entomology* | English |
| *Ophiogomphus cecilia* | Schnapauff *et al.* (1996) | *Libellula* | German |
| *Selysiothemis nigra*, *Lindenia tetraphylla* | Schneider (1981) | *Entomologische Zeitschrift* | German |
| *Ischnura evansi* | Schneider & Dumont (1997) | Book section | English |
| *Macrodiplax cora* | Schneider & Krupp (1993) | Book section | English |
| *Trithemis pallidinervis*, *Urothemis thomasi*, *Anax tristis*, *Tramea basilaris* | Schneider & Samraoui (2015) | Report section | English |
| *Trithemis aurora*, *Trithemis pallidinervis* | Schneider *et al.* (2018) | *Zootaxa* | English |
| *Calopteryx virgo* | Schorr (2012) | *International Dragonfly Fund - Report* | German |
| *Sympecma gobica*, *Sympecma paedisca*, *Sympecma fusca*, *Aeshna affinis*, *Sympetrum arenicolor*, *Sympetrum striolatum*, *Sympetrum meridionale*, *Sympetrum flaveolum* | Schröter (2010*b*) | *International Dragonfly Fund - Report* | English |
| *Sympetrum sinaiticum*, *Sympetrum arenicolor* | Schröter (2010*a*) | *Libellula* | English |
| *Aeshna affinis*, *Somatochlora arctica* | Schröter (2011) | *Libellula* | English |
| *Gomphus vulgatissimus*, *Lestes virens/ Lestes numidicus* | Schröter *et al.* (2015) | *Odonatologica* | English |
| *Sympetrum danae*, *Sympetrum flaveolum*, *Sympetrum sanguineum*, *vvulgatum* | Schröter *et al.* (2023) | *Odonatologica* | English |
| *Lestes macrostigma* | Schweighofer, Hochebner & Rotheneder (2010) | *Libellula* | German |
| *Trithemis annualata*, *Anax ephippiger* | Sciberras (2008) | *The Central Mediterranean Naturalist* | English |
| *Orthetrum nitidinerve* | Sciberras & Sammut (2013) | *Journal of the British Dragonfly Society* | English |
| *Orthetrum nitidinerve* | Sciberras *et al.* (2010) | *Libellula* | English |
| *Aeschna eremita*, *Epiaeschna heros*, *Libellula pulchella* | Shannon (1916) | *The Scientific Monthly* | English |
| *Aeshna affinis*, *Orthetrum albistylum*, *Orthetrum brunneum*, *Orthetrum coerulescens*, *Crocothemis erythraea*, *Sympetrum meridionale* | Shapoval & Buczyński (2012) | *Libellula* | English |
| *Sympetrum sanguineum*, *Sympetrum vulgatum*, *Somatochlora flavomaculata* | Shapoval & Shapoval (2017) | *Ukrainian Journal of Ecology* | English |
| *Anax parthenope*, *Crocothemis erythraea*, *Orthetrum albistylum*, *Sympetrum meridionale*, *Lestes barbarus* | Shapoval *et al.* (2022) | *Acta Biologica Sibirica* | English |
| *Libellula pulchella*, *Tramea onusta* | Shields (2016) | *Argia* | English |
| *Tholymis citrina*, *Tramea calverti* | Sibley (2012) | *Argia* | English |
| *Rhyothemis obsolescens* | Smallshire (2020) | Twitter | English |
| *Sympetrum danae*, *S flaveolum* | Smith (1998) | *Journal of the British Dragonfly Society* | English |
| *Adversaeschna brevistyla* | Smithers (1976) | *The Australian Entomologist* | English |
| *Pantala flavescens*, *Anax papuensis*, *Tramea loewii*, *Diplacodes bipunctata*, *Macrodiplax cora*, *Ischnura aurora* | Smithers & Butler (1985) | *The Australian Entomologist* | English |
| *Gynacantha nervosa*, *Brachymesia herbida*, *Erythemis vesiculosa*, *Tholymis citrina* | Smith-Patten & Hoagland (2015) | *Argia* | English |
| *Lestes macrostigma* | Soldán *et al.* (1996) | *Silva Gabreta* | English |
| *Epiaeschna heros*, *Libellula axilena*, *Libellula pulchella*, *Libellula semifasciata*, *Libellula vibrans*, *Pachydiplax longipennis* | Soltesz, Barber & Carpenter (1995) | *Argia* | English |
| *Gomphus volgantissimus*, *Sympetrum flaveolum*, *Aeshna cyanea*, *Aeshna isoceles*, *Anax imperator* | Southwood (1962) | *Biological reviews* | English |
| *Ischnura ramburii* | Sparks *et al.* (1986) | *Annals of the Entomological Society of America* | English |
| *Aeshna isoceles*, *Anax ephippiger*, *Pantala flavescens*, *Lestes barbarus* | Sparrow *et al.* (2020) | *Notulae odonatologicae* | English |
| *Tramea carolina*, *Tramea lacerata* | Sprandel (2001) | *The Florida Entomologist* | English |
| *Pantala hymenaea*, *Pantala flavescens* | Srygley (2003) | *Journal of Insect Behavior* | English |
| *Sympetrum vulgatum*, *S striolatum* | Staufer (2010) | *Libellula* | German |
| *Calopteryx virgo* | Stettmer (1996) | *European Journal of Entomology* | English |
| *Lindenia tetraphylla* | Stille, Stille & Schröter (2014) | *Libellula* | English |
| *Urothemis edwardsi* | Suárez-Tovar & Sarmiento (2016) | *Journal of Evolutionary Biology* | English |
| *Lestes malabaricus* | Subramanian (2005) | Book | English |
| *Pantala flavescens*, *Diplacodes lumians*, *Olpogastra lugubris*, *Orthetrum chrysostigma*, *Sympetrum fonscolombii*, *Tholymis tillarga*, *Crocothemis erythraea*, *Tramea basilaris*, *Trithemis kirbyi*, *Trithemus annulata*, *Zygonyx torridus*, *Africallagma glaucum*, *Pseudagrion sublacteum*, *Ischnura senegalensis*, *Agriocnemis exili* | Suhling & Martens (2007) | Book | English |
| *Sympetrum sinaiticum*, *Sympetrum fonscolombii*, *Lestes pallidus*, *Anax ephippiger*, *Anax imperator*, *Anax parthenope*, *Diplacodes lefebvrii*, *Lindenia tetraphylla*, *Pantala flavescens*, *Selysiothemis nigra*, *Zygonyx torridus* | Suhling, Jödicke & Schneider (2003) | *Cimbebasia* | English |
| *Olpogastra lugubris*, *Trithemis hecate*, *Urothemis edwardsii*, *Pantala flavescens*, *Tramea basilaris*, *Anax ephippiger*, *Sympetrum fonscolombii*, *Palpopleura lucia* | Suhling, Martens & Marais (2009) | *International Journal of Odonatology* | English |
| *Ischnura aurora*, *Olpogastra lugubris* | Suhling, Martens & Suhling (2017) | *Austral Ecology* | English |
| *Orthetrum nitidinerve*, *Lindenia tetraphylla* | Surdo (2017) | *Fragmenta Entomologica* | English |
| *Celithemis eponina*, *Erythemis simplicollis*, *Libellula vibrans*, *Pachydiplax longipennis*, *Perithemis tenera*, *Epiaeschna heros*, *Gomphaeschna furcillata* | Taber (2002) | *Banisteria* | English |
| *Selysiothemis nigra* | Taybi *et al.* (2019) | *Arxius de Miscellania Zoologica* | English |
| *Anax papuensis*, *Tramea loewii*, *Pantala flavescens*, *Hemicordulia australiae* | Theischinger & Hawking (2006) | Book | English |
| *Anax papuensis*, *Hemicordulia australiae* | Theischinger & Hawking (2021) | Book | English |
| *Tramea basilari* | Tennessen, Trapero-Quintana & De Assis Cruz Ferreira (2017) | *Zootaxa* | English |
| *Sympetrum meridionale* | Theuerkauf & Rouys (2001) | *Fragmenta Faunistica* | English |
| *Hemicordnlia tau*, *Libellula quadrimaculata*, *Pantala flavescens* | Tillyard (1917) | Book | English |
| *Adversaeschna brevistyla* | Tillyard (1940) | *Australian Zoologist* | English |
| *Pantala flavescens* | Troast *et al.* (2016) | *PLoS ONE* | English |
| *Leucorrhinia pectoralis* | Trockur (2012) | *Abhandlungen der Delattinia* | German |
| *Anax junius* | Trottier (1971) | *Canadian Entomologist* | English |
| *Zyxomma petiolatum*, *Aeshna mixta*, *Ischnura evansi* | Umar *et al.* (2012) | *International Dragonfly Fund* | English |
| *Lests barbarus* | Utzeri *et al.* (1984) | *Odonatologica* | English |
| *Miathyria simplex*, *Tramea calverti* | Vélez *et al.* (2009) | Report | English |
| *Ischnura senegalensis*, *Pantala flavescens*, *Anax ephippiger*, *Anax imperator*, *Anax parthenope* | Vieira & Cordero-Rivera (2015) | *Odonatologica* | English |
| *Rhionaeschna bonariensis*, *Rhionaeschna confusa* | Von Ellenrieder (2003) | *Tijdschrift voor Entomologie* |  |
| *Ischnura evansi* | Walker & Pittaway (1987) | Book | English |
| *Libellula quadrimaculata* | Walker (1953) | Book | English |
| *Aeshna verticalis* | Walker (1958) | Book | English |
| *Erythemis simplicicollis*, *Libellula pulchella*, *Libellula semifasciata*, *Libellula vibran*, *Pachydiplax longipennis*, *Sympetrum vicinum*, *Tramea carolina*, *Tramea lacerata*, *Anax longipes*, *Anax junius* | Walter (1996) | *Argia* | English |
| *Selysiothemis nigra*, *Trithemis annulata* | Walther *et al.* (2010) | Book | English |
| *Pantala flavescens* | Ware *et al.* (2022) | *International Journal of Odonatology* | English |
| *Sympetrum flaveolum* | Wasscher (1992) | *Natuurtijdschriften* | Dutch |
| *Anax speratus*, *Rhyothemis semihyalina*, *Selysiothemis nigra* | Waterston & Pittaway (1991) | *Journal of Oman studies* | English |
| *Erythromma viridulum* | Watts, Keat & Thompson (2010) | *Biological Invasions* | English |
| *Ischnura pumilio* | Weihrauch (2011) | *Journal of the British Dragonfly Society* | English |
| *Brachythemis leucostica* | Weihrauch & Weihrauch (2003) | *Opuscula Zoologica Fluminensia* | English |
| *Orthetrum albistylum* | Weihrauch *et al.* (2003) | *Libellula Supplement* | German |
| *Agriocnemis exilis*, *Anax rutherfordi*, *Anax tristis*, *Ischnura senegalensis* | Weihrauch *et al.* (2016) | *Boletín ROLA* | English |

## **Table S2.** List of the 18 odonate species (dragonflies/Anisoptera *N* = 13, damselflies/Zygoptera *N* = 5) designated as altitudinal migrants, and supporting citations. For 16 of the species, the cited literature describe altitudinal migrants as delaying maturation away from the breeding site, before returning to lowland breeding areas as sexually active. For two species (*Tholymis tillarga, Ceriagrion olivaceum*), delayed maturation is not mentioned in the cited reference but may still occur. Most studies were from the Palearctic (*N* = 15), followed by the Indo-Malay (*N* = 3). No altitudinal migration has been described for the Nearctic, Neotropical, Australasian or Afrotropical biogeographical regions. Note that some species listed here have distributions that include additional biogeographical regions than those noted in this table. Seven of the 18 species also engage in multi-generational migration.

| **Suborder, Family** | **Species** | **Reference** | **Biogeographical study region** | **Delayed maturation?** | **Migration type(s)** |
| --- | --- | --- | --- | --- | --- |
| Anisoptera, Libellulidae | *Potamarcha congener* | Kosterin (2012); Miller (1989) | Indo-Malay | Yes (Miller, 1989; Kosterin, 2012) | Altitudinal |
| Anisoptera, Libellulidae | *Sympetrum arenicolor* | Borisov (2006, 2009); Borisov *et al*. (2022); Schröter (2010*b*,*a*) | Palearctic | Yes (Borisov, 2006; Schröter, 2010*b*) | Altitudinal |
| Anisoptera, Libellulidae | *Sympetrum eroticum* | Ma *et al.* (2022) | Palearctic | Yes (Ma *et al.*, 2022) | Altitudinal |
| Anisoptera, Libellulidae | *Sympetrum frequens* | Asahina (1984); Corbet (2004); Sawabe *et al*. (2004) | Palearctic | Yes (Corbet, 2004) | Altitudinal |
| Anisoptera, Libellulidae | *Sympetrum meridionale* | Borisov (2006); Borisov *et al*. (2022); De Knijf & Demolder (2013); Samraoui *et al*. (1998); Schröter (2010*b*) | Palearctic | Yes (Borisov, 2006; Schröter, 2010*b*) | Altitudinal, multi-generational |
| Anisoptera, Libellulidae | *Sympetrum sanguineum* | Borisov (2006) | Palearctic | Yes (Borisov, 2006) | Altitudinal, multi-generational |
| Anisoptera, Libellulidae | *Sympetrum sinacticum* | Jödicke (1995); Jödicke *et al*. (2000) | Palearctic | Yes (Jödicke *et al*., 2000) | Altitudinal |
| Anisoptera, Libellulidae | *Sympetrum striolatum* | Borisov (2006); Pritchard (1992); Samraoui *et al*. (1998); Schröter (2010*b*) | Palearctic | Yes (Schröter, 2010*b*) | Altitudinal, multi-generational |
| Anisoptera, Libellulidae | *Tholymis tillarga* | Kosterin (2012) | Indo-Malay | Not mentioned (Kosterin, 2012) | Altitudinal, multi-generational |
| Anisoptera, Aeshnidae | *Aeshna mixta* | Samraoui *et al*. (1998); Schröter (2010*b*, 2011) | Palearctic | Yes (Schröter, 2010*b*, 2011) | Altitudinal, multi-generational |
| Anisoptera, Aeshnidae | *Anax imperator* | Samraoui & Corbet, (2000) | Palearctic | Yes (Samraoui & Corbet, 2000) | Altitudinal, multi-generational |
| Anisoptera, Aeshnidae | *Anax parthenope* | Samraoui & Corbet, (2000) | Palearctic | Yes (Samraoui & Corbet, 2000) | Altitudinal, multi-generational |
| Anisoptera, Gomphidae | *Ophiogomphus cecilia* | Schnapauff *et al.* (1996) | Palearctic | Yes (Schnapauff *et al.*, 1996) | Altitudinal |
| Zygoptera, Coenagrionidae | *Ceriagrion olivaceum* | Kosterin (2012) | Indo-Malay | Not mentioned (Kosterin, 2012) | Altitudinal |
| Zygoptera, Lestidae | *Lestes numidicus (formerly Lestes cf virens)* | Samraoui (2009); Samraoui & Corbet (2000) | Palearctic | Yes (Samraoui & Corbet, 2000) | Altitudinal |
| Zygoptera, Lestidae | *Sympecma gobica* | Borisov (2006); Borisov *et al*. (2022); Schröter (2010*b*, 2011) | Palearctic | Yes (Schröter, 2010*b*) | Altitudinal |
| Zygoptera, Lestidae | *Sympecma fusca* | Borisov *et al*. (2022); Schröter (2011) | Palearctic | Yes (Schröter, 2011) | Altitudinal |
| Zygoptera, Lestidae | *Sympecma paedisca* | Borisov *et al*. (2022); Schröter (2011) | Palearctic | Yes (Schröter, 2011) | Altitudinal |

## **References**

Abbott, J. (2007) *Dragonflies and Damselflies (Odonata) of Texas, Volym 2*. Odonata Survey of Texas.

Abbott, J. (2015) *Dragonfliles of Texas. A Field Guide*. University of Texas Press.

Acquah-Lamptey, D., Kyerematen, R. & Owusu, E.O. (2013) Dragonflies (Odonata: Anisoptera) as tools for habitat quality assessment and monitoring. *Journal of Agriculture and Biodiversity Research* **2**, 178–182.

Agüero-Pelegrin, M., Ferreras-Romero, M. & Corbet, P.S. (1999) The life cycle of *Lestes viridis* (Odonata: Lestidae) in two seasonal streams of the Sierra Morena Mountains (Southern Spain). *Aquatic Insects* **21**, 187–196.

Aistleitner, E., Barkemeyer, W., Lehmann, G. & Martens, A. (2008) A checklist of the Odonata of the Cape Verde Islands. *Mitteilungen des Internationalen Entomologischen Vereins* **33**, 45–57.

Alves, R.J.V., Costa, L.A.A., Soares, A., Silva, N.G. & Pinto, Â.P. (2019) Open ocean nocturnal insect migration in the Brazilian South Atlantic with comments on flight endurance. *PeerJ* **2019**.

Amari, H., Zebsa, R., Lazli, A., Bensouilah, S., Mellal, M.K., Mahdjoub, H., Houhamdi, M. & Khelifa, R. (2019) Differential elevational cline in the phenology and demography of two temporally isolated populations of a damselfly: Not two but one taxon? *Ecological Entomology* **44**, 93–104.

Anderson, R. (2009) Do dragonflies migrate across the western Indian Ocean? *Journal of Tropical Ecology* **25**, 347–358.

Anselin, A. (1984) First results of the new mapping-program of Odonara in Belgium. *Libellula* **3**, 78–88.

Armstrong, J.S. (1973) Odonata of the Kermadec islands. *New Zealand Entomologist* **5**, 277–283.

Asahina, S. (1984) Some biological puzzles regarding Aka-Tombo, *Sympetrum frequens*, (Anisoptera: Libellulidae) of Japan. *Advances in Odonatology* **2**, 1–11.

Asahina, S. & Turuoka, Y. (1970) Records of the insects visited a weather-ship located at the Ocean Weather Station ‘Tango’ on the Pacific, V. Insects captured during 1968. *Kontyû* **38**, 318–330.

Baccetti, N., Perrotti, E. & Utzeri, C. (1990) Dragonflies captured by ornithological ”mist nets” ( Anisoptera ). *Notulae odonatologicae* **3**, 65–80.

Bailey, R.G. & Litterick, M.R. (1993) The macroinvertebrate fauna of water hyacinth fringes in the Sudd swamps (River Nile, southern Sudan). *Hydrobiologia* **250**, 97–103.

Balinsky, B.I. (1967) On some intrinsic and environmental factors controlling the distribution of dragonflies (Odonata), with redescription and a new name for a little known species. *Journal of the Entomological Society of Southern Africa* **29**, 3–22.

Ball, L. (2014) An investigation of odonate communities within Wadi Sayq, Dhofar Province, Oman (Insecta: Odonata). *Check List* **10**, 857–863.

Barbotte, Q. & Ruffoni, A. (2018) Réflexion sur l’utilisation de l’autochtonie des 0donates à différentes échelles. *Revue scientifi que Bourgogne-Franche-Comté Nature* **27**, 277–290.

Bass, D. (2003) A Comparison of Freshwater Macroinvertebrate Communities on Small Caribbean Islands. *BioScience* **53**, 1094–1100.

Bermúdez, C. & Lopez-Victoria, M. (2009) Primeros registros de libélulas (Odonata: Anisoptera) en la Isla Malpelo, Colombia. *Revista colombiana de entomología* **35**, 127–128.

Bernard, R. (2005) First record of *Aeshna affinis* Vander Linden 1820 in Lituania (Anisoptera: Aeshnidae) and corrective notes on the Lithuanian Odonata checklist. *Notulae odonatologicae* **6**, 54–68.

Bernard, R. & Ivinskis, P. (2004) *Orthetrum brunneum* (fonscolombe, 1837), a new dragonfly species in Lithuania (Odonata: Libellulidae). *Acta Zoologica Lituanica* **14**, 31–36.

Bertram, G. & Haacks, M. (1999) Beobachtungen on windverdrifteten Libellen auf der Kurischen Nehrung im September 1998. *Libellula* **18**, 89–94.

Beukeboom, L.W. (1988) Two new records of *Somatochlora flavomaculata* from The Netherlands ( Odonata : Corduliidae ). *Entomologische berichten Amsterdam* **48**, 82–85.

Beutenmuller, W. (1890) Records of migrations of dragon flies, especially *Libellula* *quadrimaculata*, from 1868 to 1888. In *Dragon flies vs . mosquitoes . Can the mosquito pest be mitigated ? Studies in the life history of irritating insects , their natural enemies , and artificial checks by working entomologists* (ed R. Lamborn), p. D. Appleton and company, New York.

Bitzer, R. (2003) Odonates of the Middle East and Their Potential as Biological Indicators for Restoring the Mesopotamian Marshlands of Southern Iraq.

Boness, M. (2000) Massenflug der Schabrackenlibelle *Hemianax ephippiger* Burmeister an der Küste der Algarve (Portugal) (Insecto, Odonata). *Atalanta* **31**, 585.

Boomsma, T. (1993) Dragonflies and damselflies of the Shipstern Nature Reserve (Odonata). *Occasional Papers Of The Belize Natural History Society* **2**, 54–58.

Borisov, S.N. (2006) Adaptations of dragonflies (Odonata) under desert conditions. *Zoologicheskii Zhurnal* **85**, 820–829.

Borisov, S.N. (2009) Study of dragonfly (Odonata) migrations in the Western Tien Shan mountains using ornithological traps. *Entomological Review* **89**, 1025–1029.

Borisov, S.N. (2010) Geographical variation in the life cycle of *Sympecma padisc*a (Brauer 1877) (Odonata, Lestidae) in the middle region of Asia. *Eurasian Entomological Journal* **9**, 249–254.

Borisov, S.N. (2015*a*) Migrations of dragonflies (Odonata) in Central Asia: a review. Part 1. Latitudinal migrations. *Eurasian Entomological Journal* **14**, 241–256.

Borisov, S.N. (2015*b*) Dragonflies (Odonata) of thermal springs in Central Asia. *Entomological Review* **95**, 1203–1211.

Borisov, S.N., Iakovlev, I.K., Borisov, A.S., Zuev, A.G. & Tiunov, A. V. (2020) Isotope evidence for latitudinal migrations of the dragonfly *Sympetrum fonscolombii* (Odonata: Libellulidae) in Middle Asia. *Ecological Entomology* **45**, 1445–1456.

Borisov, S.N., Kazenas, V.L. & Borisov, A.S. (2022) Dragonflies (Odonata) of the Syrdarya Karatau and the Arys River valley (southern Kazakhstan) with notes on seasonal latitudinal and altitudinal migrations. *Euroasian Entomological Journal* **21**, 78–90.

Borror, D. (1953) A migratory flight of dragonflies. *Entomological news* **LXIV**, 204.

Boudot, J.-P. (2008) *Selysiothemis nigra* ( Vander Linden , 1825 ), nouveau pour le Maroc , et autres observations sur les Odonates du Maghreb nord-occidental ( Odonata : Anisoptera : Libellulidae ). *Martinia* **24**, 3–29.

Boudot, J.-P., Clausnitzer, V., Samraoui, B., Suhling, F. & Dijkstra, K-D.B. Schneider, W. (2016) *Rhyothemis semihyalin*a. The IUCN Red List of Threatened Species 2016: e.T60037A83869229.

Boudot, J.-P. & Kalkman, V.J. (2015) *Atlas of the European Dragonflies and Damselflies.* KNNNV Publishing, Netherlands.

Boudot, J.-P., Kalkman, V.J., Azpilicueta Amorin, M., Bogdanović, T., Cordero Rivera, A., Degabriele, G., Dommanget, J.-L., Ferreira, S., Garrigos, B., Jović, M., Kotarac, M., Lopau, W., Marinov, M., Mihoković, N., Riservato, E., et al. (2009) Atlas of the Odonata of the Mediterranean and North Africa. *Libellula Supplement* **9**, 1–256.

Brockhaus, T. (2012) Westpaläarktische Verbreitungsmuster von Libellen – Zeugnisse einer kaltzeitlichen Libellenfauna? Die Beispiele *Sympecma paedisca* und *Somatochlora* *metallica* (Odonata: Lestidae, Corduliidae). *Libellula Supplement* **12**, 211–226.

Brooks, S. & Jackson, K. (2001) The Odonata of Bioko, Republic of Equatorial Guinea, with the description of fan-shaped setae on early instar Libellulidae larvae. *Odonalalogica* **30**, 29–38.

Brunelle, P.-M. (1999) Distribution of Damselflies and Dragonflies (Odonata) of Maine, United States. *Northeastern Naturalist* **6**, 95–118.

Buczyński, P. (1999) Dragonflies (Odonata) of sandpits in south-eastern Poland. *Acta Hydrobiologica* **41**, 219–230.

Buczyński, P. (2006) General notes about the dragonfly (Odonata) fauna of the river Bug valley in the Lublin Region (SE Poland). In *Habitat Selection, Reproductive Behaviour and Conservation of Central European Dragonflies (Odonata)* (ed R. Buchwald), pp. 73–80. Aschenbeck & Isensee Universitätsverlag, Oldenburg.

Buczyński, P., Góral, N., Kuśnierz, A., Polak, M., Tarkowski, A. & Wróbel, A. (2019) Materiały do znajomości ważek (Odonata) Suwalszczyzny i Podlasia (Polska północno-wschodnia). *Odonatrix* **15**, 1–7.

Buczyński, P. & Moroz, M. (2004) *Aeshna* *affinis* (Vander Linden) and *Sympetrum* *depressiusculum* (Sélys) found in Belarus (Anisoptera: Aeshnidae, Libellulidae). *Notulae odonatologicae* **6**, 37–44.

Buczyński, P. & Pakulnicka, J. (2000) Odonate larvae of gravel and clay pits in the Mazurian lake district (NE Poland), with notes on extremely northern localities of some Mediterranean species. *Notulae odonatologicae* **5**, 69–84.

Buczyński, P., Shapoval, A.P. & Buczyńska, E. (2014) *Pantala flavescens* at the coast of the baltic sea (Odonata: Libellulidae). *Odonatologica* **43**, 3–11.

Buden, D.W. (2011) The Odonata of Fais Island and Ulithi and Woleai Atolls, Yap State, Western Caroline Islands, Federated States of Micronesia. *Micronesica* **41**, 215–222.

Buden, D.W. (2018) Dragonflies and Damselflies (Insecta: Odonata) of the Republic of the Marshall Islands. *Pacific Science* **72**, 373–387.

Burbach, K. & Winterholler, M. (1997) Die Invasion von *Hemianax ephippiger* (Burmeister) in Mittel- und Nordeuropa 1995/1996 (Anisoptera: Aeshnidae). *Libellula* **16**, 33–59.

Calvert, P. (1893) Catalogue of the Odonata (Dragonflies) of the Vicinity of Philadelphia, with an Introduction to the Study of This Group of Insects. *Transactions of the American Entomological Society* **20**, 152–272.

Cannings, R. (2016) *Libellula pulchella* (Twelve-spotted Skimmer): Range Expansion in Western Canada. *Argia* **28**, 27–31.

Carchini, G., Pacione, T., Tanzilli, C., Domenico, M. Di & Solimini, A. (2004) Temporalvariation of an Odonata species assemblage (Rome, Italy). *Odonalalogica* **33**, 157–168.

Carr, P. (2020) Odonata of the Chagos Archipelago, central Indian Ocean: an update. *Notulae Odonatologicae* **9**, 229–235.

Catling, P., Kostiuk, B., Kuja, S. & Kuja, A. (2017) Migrations and undirectional movements of dragonfleis in Northeastern North America. *Toronto Entomoloists’ Association, Occasional Publication*, 1–69.

Catling, P.M. (2005) Observations of possible migration of Common Baskettail, *Epitheca* *cynosura* (Say), in Ontario and New York. *Ontario Odonata* **6**, 12–13.

Catling, P.M. (2008) A New Northern Limit for Citrine Forktail (*Ischnura hastata*), Possibly Due to Climate Warming. *Argia* **20**, 12–17.

Catling, P.M. & Brownell, V.R. (1998) Migratory concentrations of dragonflies on the north shore of lake Ontario, and northward extension of migratory species. *Argia* **10**, 19–22.

Cedhagen, T. (1988) Migration of *Hemianax ephippiger* (Burmeister , 1839) (Odonata , Aeshnidae) observed in Eilath , Israel Notiser Migration hos trollsländan Hemianax ephippiger iakttagen i Israel. *Entomologisk Tidskrift* **109**, 46–48.

Cham, S. (1991) The Scarce Blue-tailed Damselfly, Ischnura pumilio (Charpentier): its habitat preferences in south-east England. *Journal of the British Dragonfly Society* **7**, 18–25.

Chari, L.D., Moyo, S. & Richoux, N.B. (2018) Trophic ecology of adult male Odonata. II. Dietary contributions of aquatic food sources. *Ecological Entomology* **43**, 15–27.

Childress, J.M. (2014) Dragonflies and Damselflies of Albemarle County, Virginia (Odonata). *Banisteria*, 28–39.

Clarke, D. (2005) *Cumbrian Dragonflies a distribution atlas*. Tullie House Museum, Carlisle.

Clausnitzer, V. (2003) Teinobasis alluaudi (Martin, 1896) from mainland Africa: Notes on ecology and biogeography (Zygoptera: Coenagrionidae). *Odonatologica* **32**, 321–334.

Clausnitzer, V. (2005) Observations on seasonality in coastal Afrotropical dragonflies. *Agrion* **9**, 18–19.

Corbet, P. (1962) *A biology of dragonflies*. H. F. & G. Witherby Ltd, London.

Corbet, P. (1984*a*) Orientation and reproductive condition of migrating dragonflies (Anisoptera). *Odonatologica* **13**, 81–88.

Corbet, P. (1984*b*) Transcript of discussion recorded during plenary session of the 7th International Symposium of Odonatology at Calgary, Alberta on 16 August 1983. *Societas Internationalis Odonatologica Rapid Communications (Supplements]* **2**.

Corbet, P. (2004) *Dragonflies. Behaviour and ecology of Odonata*. Comstock Publishing Associates, Cornell University Press, Ithaca (New York).

Corbet, P.S. (1981) Seasonal incidences of Anisoptera in light-traps in Trinidad, West Indies. *Odonalalogica* **10**, 179–187.

Corbet, P.S., Suhling, F. & Soendgerath, D. (2006) Voltinism of odonata: A review. *International Journal of Odonatology* **9**, 1–44.

Cordero Rivera, A., Carballa, M.O., Utzeri, C. & Vieira, V. (2005) Parthenogenetic Ischnura hastata (Say) widespread in the Azores (Zygoptera: Coenagrionidae). *Odonalologica* **34**, 1–9.

Corso, A., Janni, O., Pavesi, M., Sammut, M., Sciberras, A. & Viganò, M. (2012) Annotated checklist of the dragonflies (Insecta Odonata) of the islands of the Sicilian Channel, including the first re-cords of *Sympetrum sinaiticum* (Dumont, 1977) and *Pantala flavescens* (Fabricius, 1798) for Italy. *Biodiversity Journal* **3**, 459–478.

Corso, A., Janni, O., Pavesi, M. & Viganò, M. (2017) Update to the status of Pantala flavescens (Fabricius, 1798) and *Trithemis kirby*i Selys, 1891 for Italy and Central Mediterranean basin (Odonata Libellulidae). *Biodiversity Journal* **8**, 33–38.

Couteyen, S. & Papazian, M. (2011) Contribution à la connaissance des Odonates de l’île de la Réunion 10 . Zyxomma petiolatum Rambur, 1842, une espèce nouvelle pour l’île (Odonata Libellulidae). *L’Entomologiste* **67**, 21–23.

Cowan, E. & Cowan, P. (2017) The Odonata (insecta) of Northern and Central Oman. *Journal of Threatened Taxa* **9**, 10229–10232.

Craves, J.A. & O’Brien, D. (2011) *Tramea calverti* (Odonata: Libellulidae): New for Michigan with Notes on Other New Reports for the Great Lakes Region. *Great Lakes Entomologist* **44**, 78–82.

Crumrine, P.W., Switzer, P. V & Crowley, P.H. (2008) Structure and dynamics of odonate communities : accessing habitat , responding to risk , and enabling reproduction. In *Dragonflies and Damselflies: Model Organisms for Ecological and Evolutionary Research* (ed A. Córdoba-Aguilar), p. Oxford Academic Books.

D’Aguilar, J., Dommanget, J.-L. & Prechac, R. (1986) *A field guide to the dragonflies of Britain, Europe and North Africa*.

Damm, S. (2009) Conservation genetics , Speciation and Biogeography in African Dragonflies. Gottfried Wilhelm Leibniz Universität Hannover.

Damm, S. & Hadrys, H. (2012) A dragonfly in the desert: Genetic pathways of the widespread Trithemis arteriosa (Odonata: Libellulidae) suggest male-biased dispersal. *Organisms Diversity and Evolution* **12**, 267–279.

David, S. & Králiková, A. (2011) Problematika hodnocení ohroženosti – na příkladě vážek (Odonata) na Slovensku. *Životné prostredie* **45**, 264–268.

David, S. & Šácha, D. (2019) Komentový seznam vážek (Odonata) Slovenské Rebubliky. *Ochrana prírody, Banská Bystrica*, 49–78.

Dawn, P. (2014) Taxonomic study of Odonata [Insecta] in Kolkata and surroundings, West Bengal, India. *Journal of Entomology and Zoology Studies* **2**, 147–152.

De Knijf, G. & Anselin, A. (2010) When south goes north: Mediterranean dragonflies (Odonata) conquer Flanders (North-Belgium). *BioRisk* **5**, 141–153.

De Knijf, G., Anselin, A. & Goffart, P. (2002) The Belgium Odonata Atlas Project: changes in distribution. *Bulletin van het Koninklijk Belgisch Instituut voor Natuurwetenschappen. Biologie = Bulletin de l’Institut Royal des Sciences Naturelles de Belgique.* **72**, 111–112.

De Knijf, G. & Demolder, H. (2010) Odonata records from Alentejo and Algarve , southern Portugal. *Libellula* **1/2**, 61–90.

De Knijf, G. & Demolder, H. (2013) Early spring observations of Odonata from Cyprus. *Libellula* **32**, 59–74.

De Marmels, J. (1994) *Sympetrum chaconi* Spec. Nov. from Auyan-Tepui, Venezuela, with notes on a Panteouyan form of Tramea binotata (rambur) (Anisoptera; Libellulidae). *Odonalalogica* **23**, 405–412.

De Marmels, J., José, C.A. & Sharpe, C. (2008) Mass Migration of the Spot-winged Glider (*Pantala hymenaea*) in Venezuela. *Argia* **20**, 6.

De Marmels, J. & Rácenis, J. (1982) An analysis of the cophysa-group of *Tramea* Hagen, with descriptions of two new species (Anisoptera: Libellulidae). *Odonatologica* **11**, 109–128.

de Santos Loureio, N. (2013) Dragonflies and damselflies (Insecta: Odonata) collected during the Lindberg expedition to the Cape Verde Islands, 1953-54. *Zoologia Caboverdiana* **4**, 43–48.

de Santos Loureiro, N. & Martins, S. (2016) The Status of *Ischnura senegalensis* (Odonata: Coenagrionidae) in Cape Verde. *African Entomology* **24**, 448–452.

Deviche, P. & Jones, J. (2016) First Arizona and Northernmost Record of the Three-striped Dasher. *Argia* **28**, 26–27.

Devillers, P., Lafontaine, R., Pasau, B., Daems, V., Boeck, B. De, Boeckx, A., Boon, L. & Devillers-terschuren, J. (2019) La friche Josaphat à Bruxelles, Schaerbeek. Un site urbain enclavé d ’ une richesse odonatologique exceptionnelle. *Les Naturalistes belges* **100**, 1–22.

Dia, A. & Dumont, H.J. (2011) The odonata of Lebanon: (Insecta: Odonata). *Zoology in the Middle East* **52**, 63–70.

Diaz-Paniagua, C., Martín-Franquelo, R., de los Reyes, L., Fernández-Diaz, P. & Florent, P. (2014) The dragonflies of Doñana: 1959-2013 The dragonflies of Doñana : 1959-2013. *Boleín Rola* **4**.

Dijkstra, K.D.B. (2006) African Diplacodes: The status of the small species and the genus Philonomon (Odonata: Libellulidae). *International Journal of Odonatology* **9**, 119–132.

Dijkstra, K.D.B. (2007) Gone with the wind: Westward dispersal across the Indian Ocean and island speciation in Hemicordulia dragonflies (Odonata: Corduliidae). *Zootaxa* **48**, 27–48.

Dijkstra, K.D.B. & Kalkman, V. (2012) Phylogeny, classification and taxonomy of European dragonflies and damselflies (Odonata): A review. *Organisms Diversity and Evolution* **12**, 209–227.

Dijkstra, K.D.B. & Lewington, R. (2006) *Field guide to the dragonflies of Britain and Europe*. British Wildlife Publishing, Gillingham, Dorset.

Donnelly, N. (2005) Oceanic Islands in the News - or - Parthenogenesis Means Never Having to Remember Valentine’s Day. *Argia* **17**, 31–32.

DuBois, R. & Tennessen, K. (2021) The puzzling presence of *Lestes australis* (Odonata : Lestidae) in Wisconsin - does this species migrate? *The Great Lakes Entomologist* **54**, DOI: https://doi.org/10.22543/0090-0222.2376

DuBois, R. & Van den Broek, F. (2016) What is the Incomparable *Anax longipes* (Comet Darner) Doing in Wisconsin? *Argia* **28**, 16–18.

Dumont, H.J. (1967) A possible scheme of the migration *Crocothemis erythraea* (Brulle), Populations from the Camargue (Odonata: Libellulidae). *Biologisch Jaarboek* **35**, 222–227.

Dumont, H.J. (1973) The genus *Pseudagrion* (Sélys) in Israel and Egypt, with a key to the regional species (Insecta: Odonata). *Israel Journal of Zoology* **22**, 169–195.

Dumont, H.J. (1977) On migrations of *Hemianax ephippiger (*Burmeister) and *Tramea* *basilaris* (P. de Beauvois) in West and North-West Africa in the winter of 1975/1976 (Anisoptera: Aeshnidae, Libellulidae). *Odonatologica* **6**, 13–17.

Dumont, H.J. (2004) A note on dragonffies collected at light in a forest in the Ivory Coast ( West Africa ) **140**, 66–67.

Dunkle, S. (1988) Odonata of Bermuda. *Notulae Odonatologicae* **3**, 1–16.

Dunkle, S. (2000) *Dragonflies through Binoculars: A Field Guide to Dragonflies of North America*. Oxford University Press, New York.

Dunson, W.A. (1980) Adaptations of Nymphs of a Marine Dragonfly, *Erythrodiplax* *berenice*, to Wide Variations in Salinity. *Physiological Zoology* **53**, 445–452.

Dyatlova, E. & Kalkman, V. (2008) Massive migration of *Aeshna mixta* and *Sympetrum* *meridionale* in the Ukrainian Danube delta (Odonata-Anisoptera: Aeschnidae, Libellulidae). *Entomologische Berichten* **68**, 188–190.

Ebrahimi, A., Mohammadian, H. & Madjdzadeh, S.M. (2014) A note on libellulid dragonflies (Odonata: Libellulidae) of Khabr National Park (Kerman Province, South-East Iran). *Journal of the International Dragonfly Fund* **69**, 1–9.

Edelaar, P., Dijkstra, K.-D.B. & Dingemanse, N.J. (1996) *Hemianax ephippiger*: a new dragonfly for The Netherlands (Odonata: Aeshnidae). *Entomologische berichten Amsterdam* **56**, 192–195.

Eimer, T. (1882) Eine Dipteren- und Libellenwanderung beobachtet im September. *Jahreshefte des Vereins für Vaterländische Naturkunde in Württemberg* **38**, 105–113.

El Haissoufi, M., De Knijf, G., Van’T Bosch, J., Bennas, N. & Sánchez, A.M. (2015) Contribution to the knowledge of the Moroccan Odonata, with first records of *Orthetrum* *sabina*, and an overview of first and last dates for all species. *Odonatologica* **44**, 225–254.

Élouard, J.-M., Gibon, F.-M. & Gattolliat, J.-L. (2000) Les invertébrés aquatiques. In *Paysages naturels et biodiversité de Madagascar* (ed S.M. Goodman), pp. 213–226. Muséun national d’Histoire naturelle, Paris.

Farrow, R.A. (1984) Detection of transoceanic migration of insects to a remote island in the Coral Sea, Willis Island. *Australian Journal of Ecology* **9**, 253–272.

Federley, H. (1908) Einge libellulidenwanderungen über die zoologische station bei Tvärminne. *Acta Societatis pro Fauna et Flora Fennica* **31**, 1–38.

Feng, H.Q., Wu, K.M., Ni, Y.X., Cheng, D.F. & Guo, Y.Y. (2006) Nocturnal migration of dragonflies over the Bohai Sea in northern China. *Ecological Entomology* **31**, 511–520.

Ferreira, S., Grosso-Silva, J.M., Lohr, M., Weihrauch, F. & Jödicke, R. (2006) A critical checklist of the odonata of Portugal. *International Journal of Odonatology* **9**, 133–164.

Feulner, G.R., Reimer, R.W. & Hornby, R.J. (2007) An updated illustrated checlist of dragonflies and damselfliles of the UAE. *Tribulus* **17**, 37–62.

Flint, P. (2019) Observations of dragonflies (Odonata) from northern Cyprus. *Libellula* **38**, 1–28.

Fontana-Bria, L., Frago, E., Prieto-Lillo, E. & Selfa, J. (2017) Biogeographic evaluation of the dragonflies and damselflies in the Eastern Iberian Peninsula. *Arxius de Miscel·lània Zoològica,* **15**, 8–29.

Fox, A. (1989) Ischnura pumilo (Charpentier) (Odonata: Coenagriidae) - a wandering opportunitst? *The entomologist’s record and journal of variation.* **101**, 25–26.

Fraser, F.C. (1918) The influence of the monsoons on insect life in India. *The Journal of the Bombay Natural History Society* **25**, 511.

Fraser, F.C. (1924*a*) A survey of the odonate (dragonfly) fauna of western India with special remarks on the genera Macromia and Idionyx and descriptions of thirty new species. *Records of the Indian Museum* **26**, 423–522.

Fraser, F.C. (1924*b*) Report on a collection of dragonflies (Odonata) from the Andaman Islands. *Records of the Zoological Survey of India* **16**, 409–529.

Fraser, F.C. (1931) Additions to the survey of the odonate (dragonfly) fauna of Western India, with descriptions of nine new species. *Records of the Indian Museum* **33**, 443–474.

Fraser, F.C. (1933) *The Fauna of British India, including Ceylon and Burma vol 1*. Taylor & Francis, London.

Fraser, F.C. (1936) *The fauna of British India including Ceylon and Burma vol III*. Taylor & Francis, London.

Fraser, F.C. (1949) Notes on the Odonata of Mauritius. 1. The genus *Hemicordulia* Selys. *Proceedings of the Royal Entomological Society London (B)* **18**, 130–134.

Fraser, F.C. (1956) *Faune de Madagascar vol I. Insectes. Odonates Anisoptères*. L’institut de Recherche Scientifique Tananarive-Tsimbazaza, Tananarive.

Freeland, J.R., May, M., Lodge, R. & Conrad, K.F. (2003) Genetic diversity and widespread haplotypes in a migratory dragonfly, the common green darner *Anax junius.* *Ecological Entomology* **28**, 413–421.

Futahashi, R. (2017) Migration Records of *Anax guttat*us (Burmeister, 1839) (Odonata, Aeshnidae) in Tsukuba City, Ibaraki Prefecture, Japan. *Bulletin of Ibaraki Natural Museum* **20**, 37–38.

Gambles, R.M. (1960) Seasonal distribution and longevity in Nigerian dragonflies. *Journal of the West African Science Association* **6**, 18–26.

García, N., Harrison, I., Cox, N. & Tognelli, M.F. (2015) Chapter 3. The Status and Distribution of Freshwater Biodiversity in the Arabian Penninsula. In *The IUCN Red List of Threatened Species* p. Gland, Switzerland, Cambridge, UK and Arlington, USA: IUCN.

García-Díaz, J. (1938) An ecological survey of the fresh water insects of Puerto Rico. 1. The Odonata: with new life-histories. *The journal of agriculture of the University of Puerto Rico*, 43–97.

Gatter, W. (1975) Massenwanderung der Libellen *Sympetrum vulgatum* und *Sympetrum* *flaveolum* am Randecker Maar, Schwäbische Alb. *Atalanta* **6**, 193–200.

Gauci, C. (2014) A review of the Odonata of the Maltese Islands. *Journal of the British Dragonfly Society* **30**, 79–109.

Gauci, C. (2019) An update on the status of some of the less common odonate species in the Maltese Islands. *Journal of the British Dragonfly Society* **35**, 61–69.

Geijskes, D.C. (1967) Libellen gevonden op zee in het Caribisch gebied. *Entomologische Berichten* **27**, 221–223.

Gibson-Hill, C. (1950) Notes on the insects taken on Cocos-Keeling Islands. *The Bulletin of the Raffles Museum* **22**, 149–165.

Giles, G.B. (1998) An illustrated checklist of the damselflies & dragonflies of the UAE. *Tribulus* **8**, 9–15.

Glick, P.A. (1939) The distribution of insects, spiders and mites in the air. *Technical bulletin of the United States Department of Agriculture* **673**, 1–151.

Global Core Biodata Resource (GBIF) (2023) *Zyxomma petiolatum*. Https://www.gbif.org/species/1429428 [accessed 11 September 2023].

Goffart, P., Motte, G. & Vandevyvre, X. (2012) Un afflux exceptionnel de Leucorrhine à gros thorax (*Leucorrhinia pectoralis*) en Wallonie en 2012. *Les Naturalistes belges* **93**, 85–94.

Gonzáles-Soriano, E. (2008) Odonata from some west-coast Mexican islands. *Notulae odonatologicae* **7**, 13–24.

Góral, N. (2025) Are the dispersal capabilities of Zygoptera underestimated ? A critical review (Odonata). *Odonalalogica* **53**, 307–328.

Govindan, S., Ramanibai, R. & Balakrishnan, T. (2015) Distribution of dragonflies and damselflies from Pulicat lagoon, Tamilnadu. *International Journal of Multidisciplinary Research and Development* **2**, 13–16.

Goyaud, C. (2001) Atlas de répartition des Libellules (Odonata) de Vendée (1985-2000). *Revue annuelle de l’association Les Naturalistes Vendéens* **1**, 19–38.

Grainger, N., Harding, J., Drinan, T., Collier, K., Smith, B., Death, R., Makan, T. & Rollinson, E.J. (2018) Conservation status of New Zealand freshwater invertebrates , 2018. *New Zealand Threat Classification Series 28*, 29.

Grand, D. (2009) Les Libellules et le réchauffement climatique. *Revue Scientifique Bourgogne-Nature* **9**–**10**, 124–133.

Grand, D., Marinov, M., Jourdan, H., Cook, C., Rouys, S., Mille, C. & Theuerkauf, J. (2019) *Distribution, habitats, phenology and conservation of New Caledonian Odonata*. In *Zootaxa* p.

Hallworth, M.T., Marra, P.P., McFarland, K.P., Zahendra, S. & Studds, C.E. (2018) Tracking dragons: Stable isotopes reveal the annual cycle of a long-distance migratory insect. *Biology Letters* **14**, 20180741.

Hämäläinen, M., Huldén, L. & Karjalainen, S. (2004) Etelänukonkorennon (*Aeshna mixta* Latreille, 1805) vaellukset Suomeen 2002-2003 (Odonata , Aeshnidae). *Sahlbergia* **8**, 49–54.

Hančíková, B. (2011) Migration of dragonflies and damselflies (Odonata) Migrace vážek (Odonata) Migration. Charles University in Prague.

Harabiš, F., Dolný, A. & Šipoš, J. (2012) Enigmatic adult overwintering in damselflies: Coexistence as weaker intraguild competitors due to niche separation in time. *Population Ecology* **54**, 549–556.

Haring, E., Fischer, I., Sittenthaler, M., Wolf, P., Koblmüller, S., Sattmann, H. & Beqiraj, S. (2020) Intraspecific genetic diversity in selected widespread dragonfly species Intraspecific genetic diversity in selected widespread dragonfly species (Insecta : Odonata). *Acta ZooBot Austria* **157**, 239–256.

Haritonov, A. & Popova, O. (2011) Spatial displacement of Odonata in south-west Siberia. *International Journal of Odonatology* **14**, 1–10.

Hashimoto, H. & Asahina, S. (1969) Records of the insects visited a weather-ship located at the Ocean Weather Station ‘Tango’’ on the Pacific, IV. Observations on the dragonflies’. *Kontyû* **37**.

Hawking, J.H. & New, T.R. (2002) Interpreting dragonfly diversity to aid in conservation assessment: Lessons from the Odonata assemblage at Middle Creek, north-eastern Victoria, Australia. *Journal of Insect Conservation* **6**, 171–178.

Haynes, R.F. & Hillis, J.P. (1985) Report on Migrant Insects in Ireland for 1984. *Irish Naturalists’ Journal* **21**, 504–506.

Hedlund, J., Ehrnsten, E., Hayward, C., Lehmann, P., Hedlund, J., Ehrnsten, E., Hayward, C., Lehmann, P. & Hedlund, J. (2020) New records of the Paleotropical migrant *Hemianax ephippiger* in the Caribbean and a review of its status in the Neotropics. *International Journal of Odonatology* **23**, 315–325. Taylor & Francis.

Hedlund, J., Lv, H., Lehmann, P., Hu, G., Anderson, R.C. & Chapman, J.W. (2021) Unraveling the world’s longest non-stop migration: the Indian Ocean crossing of the globe skimmer dragonfly. *Frontiers in Ecology and Evolution* **9**, 1–17.

Hilfert-Rüppell, D. (2004) Optimierung des Fortpflanzungsverhaltens : Wichtige Einflußgrößen auf Territorialität und auf Paarungen von europäischen Prachtlibellenmännchen (Odonata: Zygoptera). Technical University Carola-Wilhelmina at Braunschweig.

Hobson, K.A., Anderson, R.C., Soto, D.X. & Wassenaar, L.I. (2012*a*) Isotopic Evidence That Dragonflies (*Pantala flavescens*) Migrating through the Maldives Come from the Northern Indian Subcontinent. *PLoS ONE* **7**, 9–12.

Hobson, K.A., Jinguji, H., Ichikawa, Y., Kusack, J.W. & Anderson, R.C. (2021) Long-Distance Migration of the Globe Skimmer Dragonfly to Japan Revealed Using Stable Hydrogen (δ2H) Isotopes. *Environmental Entomology* **50**, 247–255.

Hobson, K.A., Soto, D.X., Paulson, D.R., Wassenaar, L.I. & Matthews, J.H. (2012*b*) A dragonfly (δ2h) isoscape for North America: A new tool for determining natal origins of migratory aquatic emergent insects. *Methods in Ecology and Evolution* **3**, 766–772.

Hoffmann, J. (2010) Do climate changes influence dispersal and population dynamics of dragonflies in the western Peruvian Andes? *BioRisk* **5**, 47–72.

Holland, R.A., Wikelski, M. & Wilcove, D.S. (2006) How and why do insects migrate? *Science* **313**, 794–796.

Holuša, O. (2009) Notes to the first record of *Somatochlora meridionalis* (Odonata: Corduliidae) in the Czech Republic. *Acta Musei Beskidensis* **1**, 89–95.

Holuša, O. (2010) Výsledky faunistického průzkumu vážek (Insecta : Odonata) na Místecku. The results of the faunistic research of dragonflies (Insecta : Odonata) in the region of Místek town. *Acta Musei Beskidensis* **2**, 63–74.

Holuša, O. (2011) Observation of swarming behaviour in *Selysiothemis nigra* on the island of Évia, Greece (Odonata: Libellulidae). *Libellula* **30**, 233–236.

Hudson, W.H. (1895) *The naturalist in La Plata*. D. Appleton and company, New York.

Hupalo, K. & Tończyk, G. (2014) New data on the range extension of *Trithemis arteriosa* (Burmeister, 1839) (Odonata) in Turkey. *Acta Zoologica Bulgarica* **66**, 581–582.

Imms, A.D.. probable case of immigration in L. dubia (1900) A probable case of immigration in *Leucorrhinia dubia*. *The Entomologist’s Monthly Magazine* **36**, 189.

Jaramillo, A.P. (1993) Wintering Swainson’s hawks in Argentina: food and age segregation. *The Condor* **95**, 475–479.

Jödicke, R. (1995) Die larve von *Sympetrum sinaiticum tarraconensis* Jödicke (Anisoptera: Libellulidae). *Odonalalogica* **24**, 353–360.

Jödicke, R. (2000) Additions to the knoeldge of *Sympetrum sinaiticum*, Dumont (Odonata: Libellulidae). *International Journal of Odonatology* **3**, 131–140.

Johansson, F., Söderquist, M. & Bokma, F. (2009) Insect wing shape evolution: Independent effects of migratory and mate guarding flight on dragonfly wings. *Biological Journal of the Linnean Society* **97**, 362–372.

Johnson, C.G. (1969) *Migration and Dispersal of Insects by Flight*. Methuen & Co. Ldt, London.

Kalkman, V.J. (2014) *Sympetrum pedemontanum.* The IUCN Red List of Threatened Species 2014: e.T165464A19170001.

Kalniņš, M. (2009) Lesser Emperor *Anax parthenope* (SELYS , 1839) (Odonata : Aeshnidae) – a New Dragonfly Species in Latvia. *Latvijas Entomologs*, 16–20.

Katbeh-Bader, A., Amr, Z.S., Abu Baker, M. & Mahasneh, A. (2004) The dragonflies (Insecta: Odonata) of Jordan. *Denisia* **14**, 309–317.

Kathan, B. (2019) Die Libellenfauna (Insecta: Odonata) des Spitzbergs bei Tübingen unter besonderer Berücksichtigung der Arten *Sympecma fusca* (Lestidae) und Cordulegaster bidentata (Cordulegastridae). University of Tübingen.

Kharitonov, A.Y. & Popova, O.N. (2011) Migrations of dragonflies (Odonata) in the south of the West Siberian plain. *Entomological Review* **91**, 411–419.

Khelifa, R. & Zebsa, R. (2018) Rediscovery of the regionally critically endangered dragonfly *Lindenia tetraphylla* in Northeast Algeria after 170 years of apparent absence (Odonata: Gomphidae). *Notulae odonatologicae* **9**, 50–54.

Kielb, M.A. (1996) Occurrence of libellulid dragonflies (Odonata: Libellulidae) in southeastern Michigan and adjacent Essex County, Ontario. *Great Lakes Entomologist* **29**, 1–10.

Kielb, M.A. & O’Brien, M.F. (1996) Discovery of an isolated population of *Anax longipe*s in Michigan (Odonata: Aeshnidae). *Great Lakes Entomologist* **29**, 161–164.

King, S. (2011) Some Observations on the Migrant Sympetrum corruptum (Variegated Meadowhawk). *Argia* **23**, 7–8.

Knight, G.T. (2007) Large numbers of the Turnip Sawfly *Athalia rosae* (L.). *Sawfly Study Group Newsletter* **2**.

Knoblauch, A., Thoma, M. & Menz, M.H.M. (2021) Autumn southward migration of dragon fl ies along the Baltic coast and the in fl uence of weather on fl ight behaviour. *Animal Behaviour* **176**, 99–109. Elsevier Ltd.

Koch, K. & Suhling, F. (2005) Do behavioural and life-history traits vary with mate-guarding intensity in libellulid odonates? *Canadian Journal of Zoology* **83**, 1631–1637.

Kohli, M.K., Sahlén, G., Kuhn, W.R. & Ware, J.L. (2018) Extremely low genetic diversity in a circumpolar dragonfly species, *Somatochlora sahlbergi* (Insecta: Odonata: Anisoptera). *Scientific Reports* **8**, 1–10.

Kormondy, E.J. (1961) Territoriality and Dispersal in Dragonflies (Odonata). *Journal of the New York Entomological Society* **69**, 42–52.

Kosterin, O.E. (2012) A rapid survey of Odonata on Bokor Plateau, Preah Monivong National Park, Cambodia. *Cambodian Journal of Natural History*, 75–86.

Kosterin, O.E. & Borisov, S.N. (2010) Dragonflies (Odonata) of the Dzhungarskiy Alatau mountains, South-East Kazakhstan. *Eurasian Entomological Journal* **9**, 299–302.

Kosterin, O.E., Haritonov, A.Y. & Inoue, K. (2001) Dragonflies of the part of Novosibirsk Province east of the Ob’ River. *Sympetrum Hyogo* **7**–**8**, 24–49.

Kosterin, O.E. & Onishko, V. V (2021) Two newly recorded dragonfly species (Odonata: Libellulidae) for the Novosibirskaya Oblast of Russia. *Eurasian Entomological Journal* **20**, 221–228.

Kulijer, D. (2015) *Sympetrum flaveolum* in the Dinaric Alps (Odonata : Libellulidae). *Libellula* **34**, 91–101.

Kulkarni, P.P. & Prasad, M. (2002) Insecta : Odonata. In *Wetland Ecosystem Series 3: Fauna of Ujani (Maharashtra).* pp. 91–104. Zoological Survey of India, Kolkata.

Kumar, A. (1972) The phenology of dragonflies in the Dehra Dun valley, India. *Odonatologica* **1**, 199–207.

Kunz, B., Ober, S. & Jödicke, R. (2006) The distribution of *Zygonyx torridus* in the Palaearctic (Odonata: Libellulidae). *Libellula* **25**, 89–108.

Lack, D. & Lack, E. (1951) Migration of insects and birds through a Pyrenean pass. *Journal of Animal Ecology* **20**, 63–67.

Lambret, P. & Boudot, J. (2013) *Hemianax ephippiger* (Burmeister, 1839) (Odonata, Anisoptera: Aeshnidae): présentation générale. *Martinia* **Hors**-**série**, 13–28.

Lambret, P., Chelmick, D., De Knijf, G., Durand, É. & Judas, J. (2017) Odonata surveys 2010–2016 in the United Arab Emirates and the Sultanate of Oman, with emphasis on some regional heritage species. *Odonatologica* **46**, 153–205.

Landmann, A. (1983) Zum Vorkommen und Status der Feuerlibelle (*Crocothemis erythrae*a Bruellé, 1832) in Österreich (Insecta : Odonata, Libellulidae). *Berichte des naturwissenschaftlich-medizinischen Vereins in Innsbruck* **Band 70**, 105–110.

LeGrand, H. (2022) *Orthemis ferruginea* Roseate Skimmer. In *The Dragonflies and Damselflies of North Carolina* p. 120.

Lewis, J.H. (2020) Black Beach—not for the Birds: The Significance of Black Beach, New Brunswick, Canada, as a Feeding and Stopover Site for Migratory Dragonflies. *Northeastern Naturalist* **27**.

Lichtenstein, J. & Grassé, P.P. (1922) Une migration d’Odonates. *Bulletin de la Société entomologique de France* **27**, 160–163.

Lieftnick, A. (1954) Systematic list of the Odonata of Malaysia, with a survey of their known distribution. *Treubia* **22**.

Lohr, M. (2021) *Selysiothemis nigra* new for continental France (Odonata: Libellulidae). *Libellula Supplement* **16**, 177–188.

Longfield, C. (1948) A Vast Immigration of Dragonflies into the South Coast of Co. Cork. *The Irish Naturalists’ Journal* **9**, 133–141.

Lorenzo-Carballa, M., Cordero-Rivera, A. & Andrés, J.A. (2010) Islands and parthenogenesis: Genetical and ecological correlates of asexual reproduction in Ischnura Hastata (Insecta: Odonata, Coenagrionidae). In *Islands and Evolution* (eds V. Pérez-Mellado & C. Ramon), pp. 281–307. Institut Menorquí d’Estudis, Recerca.

Lorthiois, M., Cheyrezy, T., Gaudet, S., Lecomte, T. & Simon, A. (2012) Invasion de la Leucorrhine à gros thorax, *Leucorrhinia pectoralis* (Charpentier, 1825) (Odonata, Libellulidae) en Haute Normandie. *L’Entomologiste Haut-Normand* **2**, 2–10.

Lucas, Z. & Brunelle, P.-M. (2016) *Libellula pulchella* (Twelve-spotted Skimmer): A New Dragonfly Species Report for Sable Island, Nova Scotia. *Argia* **28**, 9–11.

Ma, C., Chen, S., Lou, C., Lee, I. & Hu, F. (2022) The Genus *Sympetrum* Newman, 1833 (Odonata : Libellulidae) in Taiwan: Distribution, Bionomics and a Report of a Newly Recorded Species. *Taiwanese Journal of Entomological Studies* **7**, 27–42.

Maiolini, B. & Carolli, M. (2009) Odonata in Trentino (NE-Italy): historical and recent data. *Studi Trentini in Scienze Naturali* **84**, 11–18.

Makepeace, H.S., Lewis, J.H., Sabine, D.L., Mcalpine, D.F. & Paul, M. (2017) First occurrences of the *Celithemis eponina* (Halloween Pennant) in Maritime Canada (Odonata: Libellulidae) and *Ischnura hastata* (Citrine Forktail) (Odonata: Coenagrionidae) in New Brunswick. *Journal of the Acadian Entomological Society* **13**, 46–48.

Malkmus, R. & Ruf, T. (2008) Herbstaktive Libellen in Südportugal (Odonata). *Libellula* **27**, 123–132.

Manger, R. & Dingemanse, N.J. (2009) Adult survival of *Sympecma paedisca* (Brauer) during hibernation (Zygoptera: Lestidae). *Odonatologica* **38**, 55–59.

Marinov, M. (2007) Odonata of the Western Rhodopes, with special reference to yhe wetlands north of the town of Smolyan, south Bulgaria. *Notulae odonatologicae* **6**, 97–108.

Marinov, M. (2010) Further knowledge of the colonisation of the South Island, New Zealand by *Hemicordulia australiae* (Odonata: Corduliidae). *The Weta* **39**, 17–28.

Marinov, M. (2015) The seven “oddities” of Pacific Odonata biogeography. *Journal of the International Dragonfly Fund*, 1–58.

Marshall, A.G. & Gambles, R.M. (1977) Odonata from the Guinea Savanna Zone in Ghana. *Journal of Zoology, London* **183**, 177–187.

Martens, A., de Santos Loureio, N. & Hazevoet, C.J. (2013) Dragonflies (Insecta, Odonata) collected in the Cape Verde Islands, 1960-1989, including records of two taxa new to the archipelago. *Zoologia Caboverdiana* **4**, 1–7.

May, M. (2013) A critical overview of progress in studies of migration of dragonflies (Odonata: Anisoptera), with emphasis on North America. *Journal of Insect Conservation* **17**, 1–15.

McGlone, M.S. (2006) Becoming New Zealanders: Immigration and the Formation of the Biota. In *Biological Invasions in New Zealand* (eds R.B. Allen & W.G. Lee), pp. 17–32. Springer Berlin Heidelberg, Berlin, Heidelberg.

Mediani, M., Boudot, J.P., Benazzouz, B. & El Bella, T. (2012) Two dragonfly species (Insecta: Odonata) migrating at Dakhla (region of Oued Ad-Dahab Lagouira, Morocco). *International Journal of Odonatology* **15**, 293–298.

Mendel, H. & Marsh, M.C. (1996) Invasion of dragonflies in 1995. *Transactions of the Suffolk Naturalists’ Society* **32**, 22–27.

Meurgey, F. (2008) Another Case of westward dispersal of African Odonates to the West Indies: *Trame basilaris* Palisot de Beauvois FOund in Martinique (FWI). *Argia* **20**, 20–21.

Meurgey, F. (2012) A new case of westward dispersal of an Afrotropical species to the West Indies: Keyhole Glider *Tramea basilaris* (Palisot, 1805) in Cuba (Greater Antilles). *Argia* **24**, 26–27.

Meurgey, F., Gwenaël, D., Picard, L., Poiron, C. & Questel, K. (2012) Liste Rouge provisoire des Odonates des Antilles françaises et liste des espèces à suivi prioritaire. Guadeloupe et Martinique.

Michiels, N. & Dhont, A. (1991) Characteristics of dispersal in sexually mature dragonflies. *Ecological Entomology* **16**, 449–459.

Mielewczyk, S. (1978) A new record of the mass occurence of *Aeshna* (*Hesperaeschna*) *confusa* (Rambur) on a shp in the mouth of the Rio de La Plata, Uruguay (Anisoptera: Aeshnidae). *Notulae odonatologicae* **I**, 17–36.

Miller, P.L. (1989) Communal roosting in *Potamarcha congener* (Rambur) and its possible functions (Anisoptera: Libellulidae). *Odonatologica* **18**, 179–194.

Minot, M. & Husté, A. (2022) Genetic Diversity and Structure of *Anax imperator* Leach, 1815 Populations (Odonata: Aeshnidae) in Ponds at Regional and European Scales. *Diversity* **14**.

Mitra, T.R. (1974) Another record of migratory flights of the dragonfly *Pantala flavescens* (Fabricius) (Odonata, Libellulidae) in Calcutta. *The Entomologist’s Record and Journal of Variation* **86**, 53–54.

Mitra, T.R. (1999) Biology and ecology of dragonflies (Insecta: Odonata) with notes on their adaptations in different ecosystems of India. *Records of the Zoological Survey of India* **97**, 173–188.

Miyakawa, K. (1994) Autumnal migation of mature *Sympetrum frequens* (Selys) in western Kanto Plain, Japan (Anisoptera: Libellulidae). *Odonatologica* **23**, 125–132.

Monnerat, C. (2002) Déplacements chez le genre *Sympetrum* à l’automne 1999 en Suisse occidentale (Odonata, Libellulidae). *Bulletin Romand d’Entemologie* **20**, 13–27.

Moskowitz, D., Moskowitz, J., Moskowitz, S. & Moskowitz, H. (2001) Notes on a Large Dragonfly and Butterfly Migration in New Jersey. *Northeastern Naturalist* **8**, 483–490.

Mundt, A.H. (1882) Migration of dragon-flies - *Aeshna heros* (Fabr.). *The Canadian Entomologist* **14**, 56–57.

Murdoch, D. (1998) The size of the 1995 influx of *Sympetrum flaveolum* (L.). *Journal of the British Dragonfly Society* **14**, 11–12.

Nair, M. V. (2011) Dragonflies and Damselflies of Orissa and Eastern India. Wildlife Organization, Forest and Environment Department, Government of Orissa.

Natural Heritage & Endangered Species Program (2015) Comet darnet *Anax longipes*.

Nelson, B., Ronayne, C. & Thompson, R. (2003) Colonization and changing status of four Odonata species, *Anax imperator, Anax parthenope, Aeshna mixta* and *Sympetrum fonscolombii*, in Ireland 2000-2002. *Irish Naturalists’ Journal* **27**, 266–272.

Nielsen, O.F. (1994) Anax imperator (Leach, 1815) - ny dansk guldsmed. (Odonara, Aeshnidae). *Entomologiske Meddelelser* **624**, 97–99.

Nikula, B.J., Loose, J.L. & Burne, M.R. (2003) *A Field Guide to the Dragonflies and Damselflies of Massachusetts*. Massachusetts Division of Fisheries & Wildlife, Natural Heritage & Endangered Species Program.

Nikula, B.J., Sones, J.L. & Trimble, J.R. (2001) New and Notable Records of Odonata from Massachusetts. *Northeastern Naturalist* **8**, 337–342.

Norman, H.H. (1957) *Cordulia aenea* L. och *Somatochlora arctica* Zett. på migration i Skärgårdshavet i SW-Finland (Odonata). *Notulae entomologicae* **37**, 30.

Olberg, S. & Lønnve, O.J. (2012) *Ischnura pumilio* (Charpentier, 1825) (Odonata, Coenagrionidae) in Norway. *Norwegian Journal of Entomology* **59**, 229–233.

Olsvik, H. & Hämäläinen, M. (1992) Dragonfly records from the Maldive Islands, Indian Ocean (Odonata). *Opuscula Zoologica Fluminensia* **89**, 1–7.

Onyango-Odiy, P.O. (1973) Some observations on butterfly and dragonfly migration in the Kenya highlands. *Entomologists monthly magazine* **109**, 141–147.

Ott, J. (1988) Beiträge zur Biologie und zum Status von Crocothemis erythraea (BRÜLLE , 1832). *Libellula* **7**, 1–25.

Ott, J. & Samways, M.J. (2010) Chapter 3. Effects of climatic changes on Odonata: are the impacts likely to be the same in the northern and southern hemispheres? In *Atlas of Biodiversity Risk* (eds L. Penev, T. Georgiev, R. Grabaum, V. Grobelnik, V. Hammen, S. Klotz, M. Kotarac & I. Kuhn), pp. 84–85. CIPRA International.

Palacino R., F. & Millán, C.A. (2010) First records of possible migratory dragonflies in Colombia. *Argia* **22**, 9–10.

Parr, A. (1996) Dragonfly movement and migration in Britain and Ireland. *Journal of the British Dragonfly Society* **12**, 33–47.

Parr, A. (1997) Migrant and dispersive dragonflies in Britain during 1996. *Journal of the British Dragonfly Society* **13**, 41–47.

Parr, A. (2000) Overzicht van recente veranderingen in de libellenfauna van Engeland & Ierland. *NVL-Nieuwsbrief* **3**, 13–14.

Parr, A. (2001) Migrant and dispersive dragonflies in Britain during 2000. *Journal of the British Dragonfly Society* **17**, 49–54.

Parr, A. (2010) Migrant Dragonflies in the UK: Distributions are flexible, especially in times of climate change. In *1st European Congress on Odonatology* p.

Parr, A. (2013) The Large White-faced Darter *Leucorrhinia pectoralis* (Charp.) in Britain during 2012. *Journal of the British Dragonfly Society* **29**, 40–45.

Parr, A. (2014) Migrant and dispersive dragonflies in Britain during 2012 and 2013. *Journal of the British Dragonfly Society* **30**, 110–124.

Paulson, D. (2009) *Dragonflies and damselflies of the West*. Princeton University Press, Princeton, New Jersey.

Paulson, D., Abbott, J., May, M., Jones, C., Blackburn, M. & Mazzacano, C. (2016) Field guide to migratory dragonflies.

Paulson, D.R. (1966) The dragonflies (Odonata: Anisoptera) of southern Florida. University of Miami.

Paulson, D.R. (2001) Recent odonata records from Southern Florida - effects of global warming? *International Journal of Odonatology* **4**, 57–69.

Paulson, D.R. (2002) Odonata records from Nayarit and Sinaloa, Mexico, with comments on natural history and biogeography. *Odonalalogica* **31**, 359–370.

Paulson, D.R. (2012) *Dragonflies and Damselflies of the East*. Princeton University Press. (2011), Princeton.

Peck, S.B. (1994) Aerial Dispersal of Insects Between and to Islands in the Galápagos Archipelago, Ecuador. *Annals of the Entomological Society of America* **87**, 218–224.

Pickwell, A., Gennard, D.E., Taylor, P. & Chadd, R. (2012) The Norfolk Hawker Aeshna isosceles (Müller): a step further from the brink? *Journal of the British Dragonfly Society* **28**, 44–57.

Pinhey, E. (1951) The dragonflies of Southern Africa: Chapter 7. Suborder Anisoptera Sélys. *Transvaal Museum Memoirs* **5**, 136–182.

Pinhey, E. (1974) Odonata of the Northwest Cameroons and particularly of the islands stretching southwards from the Guinea Gulf. *Bonner zoologische Beiträge* **1**–**3**, 180–212.

Pinhey, E. (1978) Odonata. In *Biogeography and Ecology of Southern Africa* (ed M. Werger), p. Dr W. Junk by Publishers The Hague.

Pinhey, E. (1985) A Survey of the Dragonflies (Odonata) of South Africa. Part 2 Anisoptera. *Journal of the Entomological Society of Southern Africa* **48**, 1–48.

Pittman, S. (1996) Migrant species of *Sympetrum* in Norfolk 1995. *Journal of the British Dragonfly Society* **12**, 1–2.

Polette, P.P., Abbott, C., Gouys, J., Jenard, P., Juliand, P., Darnaud, S. & Boudot, J. (2019) Premières mentions de Trithemis kirbyi (Odonata : Libellulidae) en France. *Martinia* **33**, 15–25.

Popova, O.N. & Eremina, E.E. (2016) *Sympetrum fonscolombii* (Selys, 1840) (Odonata, Libellulidae) in northernmost areal localities in Chelyabinskaya and Novosibirskaya Oblast’s of Russia. *Euroasian Entomological Journal* **15**, 45–59.

Popova, O.N. & Haritonov, A.Y. (2014) Mass reproductive wanderings of dragonflies of the genus *Sympetrum* (Odonata, Libellulidae). *Entomological Review* **94**, 21–28.

Prather, B. & Prather, I. (2015) The dragonflies and damselflies (Odonata) of Colorado: An updated annotated checklist. In *Insects of Western North America* p. 59.

Pritchard, G. (1992) Current topics in dragonfly biology Vol 5. *Societas Internationalis Odonatologica Rapid Communications (Supplements)*, 1–28.

Račkauskaitė, D. & Gliwa, B. (2015) First record of *Crocothemis erythraea* (Odonata: Libellulidae) in Lithuania. *New and rare for Lithuania insect species* **27**, 5–6.

Reece, B.A. & Mcintyre, N.E. (2009) Community assemblage patterns of odonates inhabiting a wetland complex influenced by anthropogenic disturbance. *Insect Conservation and Diversity* **2**, 73–80.

Reels, G.T. (2011) Emergence patterns and adult flight season of Anisoptera at a managed wetland site in Hong Kong, southern China. *International Journal of Odonatology* **14**, 33–48.

Reels, G.T. (2019) An annotated check list of Hong Kong dragonflies and assessment of their local conservation significanc. *Journal of the International Dragonfly Fund*, 1–49.

Reichholf, J. (1973) A migration of *Pantala flavescens* (Fabricius, 1798) along the shore of Santa Catarina, Brazil (Anisoptera: Libellulidae). *Odonatologica* **2**, 121–124.

Reimer, R.W., Feulner, G.R. & Hornby, R.J. (2009) Errata and addenda updated illustrated checklist of dragonflies of the UAE - including a third species of Ischnura damselfly. *Tribulus* **18**, 28–36.

Rimantas Stonis, J., Dapkus, D., Auksoriūte, A. & Vilkas, A. (2009) Žirgelių (insecta, odonata) taksonominis lietuviškų vardų sąrašas. *Naujos ir retos lietuvos vabdziu rusys* **21**, 155–165.

Ris, F. (1904) Odonaten. *Ergebnisse der Hamburger Magalhaenischen Sammelreise*, 7.

Roble, S.M. (2005) Observations on an Aggregation of Gomphaeschna furcillata in Southeastern Virginia. *Argia* **17**, 8–9.

Root, F.M. (1910) Dragon flies collected at Point Pelee and Pelee Island, Ontario, in the summers of 1910 and 1911. *The Canadian Entomologist* **44**, 208–209.

Rosario, K., Marinov, M., Stainton, D., Kraberger, S., Wiltshire, E.J., Collings, D.A., Walters, M., Martin, D.P., Breitbart, M. & Varsani, A. (2011) Dragonfly cyclovirus, a novel single-stranded DNA virus discovered in dragonflies (Odonata: Anisoptera). *Journal of General Virology* **92**, 1302–1308.

Rothfels, C.J. (2007) The Comet Darner (*Anax longipes*: Aeshnidae): Possibly breeding in Canada. *Ontario Odonata* **7**, 30–33.

Rowe, R.J. (1978) Ischnura aurora (Brauer), a dragonfly with unusual mating behaviour (Zygoptera: Coenagrionidae). *Odonalalogica* **7**, 375–383.

Rowe, R.J. (1980) Records of the dragonfly *Pantala flavescens* in New Zealand, with notes on *Tramea transmarina* a possible immigrant (Odonata: Libellulidae). *New Zealand Entomologist* **7**, 139–141.

Ruddek, J. (1998) Odonata over the Andaman Sea. *Notulae odonatologicae* **5**.

Rudolph, D. & Fisher, C. (1993) Swainson’s Hawk predation on dragonflies in Argentina. *The Wilson bulletin (Wilson Ornithological Society)* **105**, 365–366.

Ruiter, E.J., Uilhoorn, H.M.G., Manger, R., Ketelaar, R. & de Boer, E.P. (2007) Terugvangsten van Noordse winterjuffers (*Sympecma paedisca*) over grote afstand. *Brachytron* **11**, 34–41.

Russell, R.W. (2005) Interactions Between Migrating Birds and Offshore Oil and Gas Platforms in the Northern Gulf of Mexico. *Final Report*, 1–325.

Russell, R.W., May, M.L., Soltesz, K.L. & Fitzpatrick, J.W. (1998) Massive Swarm Migrations of Dragonflies (Odonata) in Eastern North America. *The American Midland Naturalist* **140**, 325–342.

Sakagami, S.F. (1961) An Ecological Perspective of Marcus Island, with Special Reference to Land Animals. *Pacific Science* **15**, 82–104.

Samraoui, B. (2009) Seasonal ecology of algerian Lestidae (Odonata). *International Journal of Odonatology* **12**, 383–394.

Samraoui, B., Bouzid, S., Boulahbal, R. & Corbet, P.S. (1998) Postponed reproductive maturation in upland refuges maintains life-cycle continuity during the hot, dry season in algerian dragonflies (anisoptera). *International Journal of Odonatology* **1**, 119–135.

Samraoui, B. & Corbet, P.S. (2000) The odonata of numidia, northeastern Algeria part II seasonal ecology. *International Journal of Odonatology* **3**, 27–39.

Samways, M.J. (1999) Diversity and conservation status of South African dragonflies (Odonata). *Odonatologica* **28**, 13–62.

Samways, M.J. (2003) Conservation of an endemic odonate fauna in the Seychelles archipelago. *Odonalalogica* **32**, 177–182.

Samways, M.J. (2010*a*) Impacts of extreme weather and climate change on South African dragonflies. *BioRisk* **5**, 73–84.

Samways, M.J. (2010*b*) The rare Ghost Duskdarter dragonfly *Zyxomma petiolatum* on Desroches Island , Seychelles. *Phelsuma* **18**, 98.

Sánchez Estrada, D.M. (2018) Odonatos (Insecta: Odonata) en dos localidades del Valle de Tulancingo- Acaxochitlán, Hidalgo, México.

Sánchez-Herrera, M. & Ware, J. (2012) Biogeography of Dragonflies and Damselflies: Highly Mobile Predators. In *Global Advances in Biogeography* (ed L. Stevens), pp. 291–306. InTech, Rijeka, Croatia.

Sawabe, K., Uéda, T., Higashi, K. & Lee, S.M. (2004) Genetic identity of Japanese *Sympetrum frequens* and Korean *S. depressiusculum* inferred from mitochondrial 16S rRNA sequences (Odonata: Libellulidae). *International Journal of Odonatology* **7**, 517–527.

Schaub, M. (1997) Ein Massenzug von Libellula quadrimaculata entlang der Kurischen Nehrung (Anisoptera: Libellulidae). *Libellula* **16**, 181–184.

Schenk, K., Suhling, F. & Martens, A. (2004) Egg distribution, mate-guarding intensity and offspring characteristics in dragonflies (Odonata). *Animal Behaviour* **68**, 599–606.

Schiel, F. & Kunz, B. (2005) Zur aktuellen Bestandsentwicklung von *Lestes barbarus*, Aeshna affinis und Sympetrum meridionale in zwei Regionen Baden-Württembergs (Odonata : Lestidae, Aeshnidae, Libellulidae). *Libellula* **24**, 163–190.

Schilling Gaenzle, E., Kardynal, K.J., Kundel, H., Crews-Erjaveck, Z., Zobitz, J.M. & Hobson, K.A. (2021) Phenological and isotopic evidence for migration as a life history strategy in Aeshna canadensis (family : Aeshnidae) dragonflies. *Ecological Entomology* **46**, 209–219.

Schnapauff, I., Schridde, P., Suhling, F. & Ullmann, K. (1996) Libellenbeobachtungen in Nordost-Griechenland. *Libellula* **15**, 169–183.

Schneider, T. & Dumont, H.J. (1997) The dragonflies and damselflies (Insecta: Odonata) of Oman. An updated and annotated checklist. In *Fauna of Saudi Arabia 16* (eds F. Krupp & V. Mahnert), pp. 89–110. Karger Libri.

Schneider, T., Ikemeyer, D., Müller, O.L.E. & Dumont, H.J. (2018) Checklist of the dragonflies (Odonata) of Iran with new records and notes on distribution and taxonomy. *Zootaxa* **4394**, 1–40.

Schneider, W. (1981) Eine Massenwanderung von *Selysiothemis nigra* (Vander Linden, 1825)(Odonata: Macrodiplactidae) und *Lindenia tetraphylla* (Vander Linden, 1825)(Odonata: Gomphidae) in Südjordanien. *Entomologische Zeitschrift* **91**, 97–102.

Schneider, W. & Krupp, F. (1993) Dragonfly records from Saudi Arabia, with an Annotated Checklist of the Species from the Arabian Peninsula (Insecta: Odonata). In *Fauna of Saudi Arabia 13* pp. 63–78.

Schneider, W. & Samraoui, B. (2015) The status and distribution of dragonflies and damselflies (Odonata) in the Arabian Peninsula. In *IUCN Red List: The status and distribution of freshwater biodiversity in the Arabian Peninsula* (eds N. García, I. Harrison, N. Cox & M.F. Tognelli), pp. 39–53. Gland, Switzerland, Cambridge, UK and Arlington, USA: IUCN.

Schorr, M. (2012) *Libellula virgo* Linnaeus, 1758 auf Grönland – Eine Neubewertung der Beobachtung von Fabricius (1780). *International Dragonfly Fund - Report* **52**, 1–44.

Schröter, A. (2010*a*) The Odonata of Kyrgyzstan, part I - Critical national checklist, annotated list of records and collected data of the half 2008 and 2009. *International Dragonfly Fund - Report* **28**, 1–72.

Schröter, A. (2010*b*) On a collection of dragonflies from eastern Georgia, with the first record of Sympetrum arenicolor (Odonata: Libellulidae). *Libellula* **29**, 209–222.

Schröter, A. (2011) A mass migration of *Aeshna affinis* in southern Kyrgyzstan: attempt to provide a spatial and temporal reconstruction (Odonata: Aeshnidae). *Libellula* **30**, 203–232.

Schröter, A., Borkenstein, A. & Jödicke, R. (2023) Why do mature dragonflies migrate ? A critical analysis of Corbet ’ s chapter “ Spatial displacement by flight ” with reference to *Sympetrum striolatum* (Odonata : Libellulidae). *Odonatologica* **52**, 61–78.

Schröter, A., Seehausen, M., Kunz, B., Günther, A., Schneider, T. & Jödicke, R. (2015) Update of the Odonata fauna of Georgia, southern Caucasus ecoregion. *Odonatologica* **44**, 279–342.

Schweighofer, W., Hochebner, T. & Rotheneder, G. (2010) *Lestes macrostigma* im westlichen Niederösterreich (Odonata: Lestidae) Einleitung Untersuchungsgebiet und Methode. *Libellula* **29**, 175–182.

Sciberras, A. (2008) A contribution to the knowledge of odonata in the Maltese islands. *The Central Mediterranean Naturalist* **4**, 275–288.

Sciberras, A. & Sammut, M. (2013) The occurrence of the Copper Emerald *Calopteryx haemorrhoidalis* (Vander Linden, 1825), records of rare species, changing population trends of some hitherto common species and recent colonisers in the Maltese Islands. *Journal of the British Dragonfly Society* **28**, 1–9.

Sciberras, A., Sciberras, J. & Kunz, B. (2010) Orthetrum nitidinerve new to the Maltese Islands (Odonata: Libellulidae). *Libellula* **29**, 55–60.

Shannon, H.J. (1916) Insect Migrations as Related to Those of Birds. *The Scientific Monthly* **3**, 227–240.

Shapoval, A. & Buczyński, P. (2012) Remarkable Odonata caught in ornithological traps on the Courish Spit, Kaliningrad Oblast, Russia. *Libellula* **31**, 97–109.

Shapoval, N.A. & Shapoval, A. (2017) Annotated checklist of the dragonflies (Insecta: Odonata) of the Kaliningrad region, north-western Russia. *Ukrainian Journal of Ecology* **7**, 22–29.

Shapoval, N.A., Shapoval, G.N. & Shapoval, A.P. (2022) New, rare and vagrant damselflies and dragonflies (Insecta : Odonata) in the Kaliningrad Oblast , north-western Russia. *Acta Biologica Sibirica* **8**, 261–279.

Shields, M.A. (2016) Prolonged Flight Seasons of Odonates in North Carolina Associated with Record-breaking Warmth. *Argia* **28**, 11–12.

Sibley, F.C. (2012) Having a Blast in Barbados. *Argia* **24**, 1–28.

Smallshire, D. (2020) *Rhyothemis obsolescens*. *X*. Https://x.com/smallshire/status/1259442630955581441 [accessed 14 January 2026].

Smith, P.H. (1998) Dispersion or migration of *Sympetrum danae* (Sulzer) in South Lancashire. *Journal of the British Dragonfly Society* **14**, 12–14.

Smith-Patten, B.D. & Hoagland, B.W. (2015) First Record of the Thornbush Dasher (Micrathyria hagenii) for Oklahoma. *Argia* **27**, 25–28.

Smithers, C.N. (1976) A record of ‘Aeshna brevistyla’ Rambur (Odonata: Aeshnidae) from Norfolk Island. *The Australian Entomologist* **3**.

Smithers, C.N. & Butler, W.H. (1985) Dragonflies and damselflies (Odonata) from Barrow and nearby islands off the coast of Western Australia. *The Australian Entomologist* **12**, 9–12.

Soldán, T., Papáček, M., Novak, K. & Zelený, J. (1996) The Šumava Mountains: a unique biocentre of aquatic insects (Ephemeroptera, Odonata, Plecoptera, Megaloptera, Trichoptera and Heteroptera-Nepomorpha). *Silva Gabreta* **1**, 179–186.

Soltesz, K., Barber, B. & Carpenter, G. (1995) A spring dragonfly migration in the northeast. *Argia* **7**.

Southwood, T.R.E. (1962) Migration of Terrestrial Arthropods in Relation To Habitat. *Biological Reviews* **37**, 171–211.

Sparks, A.N., Jackson, R.D., Carpenter, J.E. & Muller, R.A. (1986) Insects Captured in Light Traps in the Gulf of Mexico. *Annals of the Entomological Society of America* **79**, 132–139.

Sparrow, D.J., Makris, C., Sparrow, R., Michaelides, M., Konis, D. & Knijf, G. De (2020) First records of *Aeshna isoceles* and the rediscovery of *Lestes barbarus* on Cyprus (Odonata: Lestidae, Aeshnidae). *Notulae odonatologicae* **9**, 185–195. Osmylus Scientific Publishers.

Sprandel, G.L. (2001) Fall Dragonfly (Odonata) and Butterfly (Lepidoptera) Migration at St . Joseph Peninsula, Gulf County, Florida. *The Florida Entomologist* **84**, 234–238.

Srygley, R.B. (2003) Wind drift compensation in migrating dragonflies *Pantala* (Odonata: Libellulidae). *Journal of Insect Behavior* **16**, 217–232.

Staufer, M. (2010) Beobachtungen zur Mortalität wandernder *Sympetrum striolatum* und *S. vulgatum* an einem Autobahnzubringer im Nordburgenland (Odonata: Libellulidae). *Libellula* **29**, 183–196.

Stettmer, C. (1996) Colonisation and dispersal patterns of banded (Calopteryx splendens) and beuaitful demoiselles (C virgo) (Odonata: Calopterygidae) in sout-east German sites. *European Journal of Entomology* **93**, 579–593.

Stille, M., Stille, B. & Schröter, A. (2014) Lindenia tetraphylla – new for the island of Kérkira (Corfu), Greece (Odonata : Gomphidae). *Libellula* **8**, 77–116.

Suárez-Tovar, C.M. & Sarmiento, C.E. (2016) Beyond the wing planform: Morphological differentiation between migratory and nonmigratory dragonfly species. *Journal of Evolutionary Biology* **29**, 690–703.

Subramanian, K.A. (2005) *Dragonflies and Damselflies of Peninsular India - A field guide*. Indian Academy of Sciences, Bangalore.

Suhling, F., Jödicke, R. & Schneider, W. (2003) Odonata of African arid regions - are there desert species? *Cimbebasia* **18**, 207–224.

Suhling, F. & Martens, A. (2007) *Dragronflies and Damselflies of Namibia*. Gamsberg Macmillan Publishers, Windhoek, Namibia.

Suhling, F., Martens, A. & Marais, E. (2009) How to enter a desert—patterns of Odonata colonisation of arid Namibia. *International Journal of Odonatology* **12**, 287–308.

Suhling, F., Martens, A. & Suhling, I. (2017) Long-distance dispersal in Odonata: Examples from arid Namibia. *Austral Ecology* **42**, 544–552.

Surdo, S. (2017) First record of *Lindenia tetraphylla* (Vander Linden, 1825) and rediscovery of Orthetrum nitidinerve (Selys, 1841) in Sicily (Insecta: Odonata). *Fragmenta Entomologica* **49**, 185–189.

Taber, B. (2002) Spring dragonfly (Odonata) and Butterfly (Lepidoptera) fallout at the Chesapeake Bay Bridge-tunnel. *Banisteria* **19**, 26–27.

Taybi, A.F., Mabrouki, Y., Berrahou, A., Sbaa, M. & Brochard, C. (2019) New data on the dragonfly fauna (Odonata) of the moulouya river basin and the oriental region, morocco. *Arxius de Miscellania Zoologica* **17**, 85–108.

Tennessen, K.J., Trapero-Quintana, A. & De Assis Cruz Ferreira, F. (2017) Description of the Nymph of Tramea binotata (Rambur, 1842) (Odonata: Libellulidae). *Zootaxa* **4337**, 445–450.

Theischinger, G. & Hawking, J. (2006) *Complete Field Guide to Dragonflies of Australia*. In *Complete Field Guide to Dragonflies of Australia* p. CSIRO Publishing, Collingwood.

Theischinger, G. & Hawking, J. (2021) *Complete Field Guide to Dragonflies of Australia*Second edi. CSIRO Publishing, Melburne.

Theuerkauf, J. & Rouys, S. (2001) Habitats of Odonata in the Białowieża Forest and its surroundings (Poland). *Fragmenta Faunistica* **44**, 33–39.

Tillyard, R.J. (1917) *The Biology of dragonflies (Odonata or Paraneuroptera)*. Cambridge University Press, London.

Tillyard, R.J. (1940) A reclassification of the order Odonata. Based on some new interpretations of the venation of the dragonfly wing. *Australian Zoologist* **9**, 359–396.

Troast, D., Suhling, F., Jinguji, H., Sahlén, G. & Ware, J. (2016) A global population genetic study of Pantala flavescens. *PLoS ONE* **11**, 1–13.

Trockur, B. (2012) Erstnachweis der Großen Moosjungfer Leucorrhinia pectoralis (Charpentier , 1825) im Saarland (Odonata : Libellulidae). *Abhandlungen der Delattinia* **38**, 255–265.

Trottier, R. (1971) Effect of temperature on the life-cycle of anax junzus (Odonata: Aeshnidae) in Canada. *The Canadian Entomologist* **103**, 1671–1683.

Umar, D.M., Marinov, M.G., Schorr, M. & Chapman, H.M. (2012) Odonata attracted by light – a new topic for myth-busters. *International Dragonfly Fund* **43**, 1–52.

Utzeri, C., Carchini, G., Falchetti, E. & Belfiore, C. (1984) Philopatry, homing and dispersal in Lestes barbarus (Fabricius) (Zygoptera: Lesstidae). *Odonatologica* **13**, 573–584.

Vélez, Á.U., Posada, C.C., Mora, C.P., Forero, B.C., Rodríguez, C.L., Higgins, M. Lou, Naranjo, L.G. & Espinel, J.D.A. (2009) *Plan Nacional de las Especies Migratorias: Diagnóstico e identificación de acciones para la conservación y el manejo sostenible de las especies migratorias de la biodiversidad en Colombia*. Ministro De ambiente, vivienda y desarrollo territorial.

Vieira, V. & Cordero-Rivera, A. (2015) First record of Pantala flavescens from the Azores (Odonata: Libellulidae). *Odonatologica* **44**, 1–9.

Von Ellenrieder, N. (2003) A synopsis of the neotropical species of ‘Aeshna’ Fabricius: The Genus *Rhionaeschna* Förster (Odonata: Aeshnidae). *Tijdschrift voor Entomologie* **146**, 67–207.

Walker, D. & Pittaway, A. (1987) Insects of Eastern Arabia. Macmillan Publichers Ltd, London and Basingstoke.

Walker, E. (1953) *The Odonata of Canada and Alaska volume one*. In *The Odonata of Canada and Alaska* p. University of Toronto Press, Canada.

Walker, E. (1958) *The Odonata of Canada and Alaska volume two*. University of Toronto Press, Canada.

Walter, S. (1996) Fort Tilden Dragonfly Migration Watch , 1993-1995. *Fort Tilden dragonfly migration watch: results of a three- year study, 1993-1995.* **8**, 8–12.

Walther, G.-R., Nagy, L., Heikkinen, R., Penuelas, J., Ott, J., Pauli, H., Pöyry, J., Silje, B. & Hickler, T. (2010) Observed Climate-Biodiversity Relationships. In *Atlas of Biodiversity Risk* pp. 74–75.

Ware, J., Kohli, M.K., Mendoza, C.M., Troast, D., Jinguji, H., Hobson, K.A., Sahlén, G., Anderson, R.C. & Suhling, F. (2022) Evidence for widespread gene flow and migration in the Globe Skimmer dragonfly Pantala flavescens. *International Journal of Odonatology* **25**, 43–55.

Wasscher, M. (1992) Vragen omtrend het voorkomen van Symetrum flaveolum in Nederland. *Natuurtijdschriften* **21**, 13–17.

Waterston, A. & Pittaway, A. (1991) The Odonata or Dragonflies of Oman and neighbouring territories. *Journal of Oman Studies* **10**, 131–168.

Watts, P.C., Keat, S. & Thompson, D.J. (2010) Patterns of spatial genetic structure and diversity at the onset of a rapid range expansion: Colonisation of the UK by the small red-eyed damselfly *Erythromma viridulum*. *Biological Invasions* **12**, 3887–3903.

Weihrauch, F. (2011) A review of the distribution of Odonata in the Macaronesian Islands, with particular reference to the Ischnura puzzle. *Journal of the British Dragonfly Society* **27**, 28–46.

Weihrauch, F., Burbach, K., Hölken, U., Netz, H.J. & Stettmer, C. (2003) Neue Nachweise von Orthetrum albistylum aus Bayern (Odonata: Libellulidae). *Libellula Supplement* **4**, 59–70.

Weihrauch, F., Vieira, V., Cordero-Rivera, A. & de Santos Loureiro, N. (2016) Update on the zoogeography of Odonata in the Macaronesian Islands. *Boletín ROLA* **8**, 9–22.

Weihrauch, F. & Weihrauch, S. (2003) Spring Odonata records from Alentejo (Portugal), Andalusia and Extremadura (Spain). *Opuscula Zoologica Fluminensia* **207**, 1–18.
